# Exploring the barriers to prehabilitation integration in Austrian healthcare: a qualitative analysis

**DOI:** 10.1016/j.bjao.2026.100868

**Published:** 2026-10-06

**Authors:** Jan Markus Lutz, Mia Gisselbaek, Ana Beatriz Noronha, Joana Berger-Estilita

**Affiliations:** 1 Department of Anaesthesiology, Krankenhaus Oberndorf, Oberndorf bei Salzburg, Austria; 2 Department of Anesthesia, McGill University, Montreal, Canada; 3 Division of Anaesthesiology, Department of Anaesthesiology, Clinical Pharmacology, Intensive Care and Emergency Medicine, Geneva University Hospitals and Faculty of Medicine, Geneva, Switzerland; 4 Division of Pediatric Anaesthesiology, Montreal Children’s Hospital, McGill University Health Center, Montreal, Quebec, Canada; 5 Department of Anaesthesiology, Perioperative Medicine and General Intensive Care, Landeskrankenhaus, Uniklinikum Salzburg, Salzburg, Austria; 6 Institute for Medical Education, University of Bern, Switzerland; 7 Institute of Anaesthesiology and Intensive Care, Salemspital, Hirslanden Medical Group, Bern, Switzerland; 8 Rise-HEALTH, Centre for Health Technology and Services Research, Faculty of Medicine, University of Porto, Porto, Portugal; 9 Department of Anesthesiology, University Medical Centre Groningen, University of Groningen, Groningen, The Netherlands

**Keywords:** Austria, healthcare system readiness, implementation barriers, perioperative care, prehabilitation

## Abstract

**Background:**

Prehabilitation optimises patients’ physical, nutritional, and psychological readiness before major surgery, but remains largely absent from Austrian clinical practice. We aimed to identify stakeholder-perceived barriers to prehabilitation implementation and assess the readiness of the healthcare system.

**Methods:**

An exploratory qualitative study using semi-structured interviews was conducted with eight purposively sampled stakeholders representing anaesthesiology (*n*=2), surgery (*n*=2), outpatient cardiology (*n*=1), clinical psychology (*n*=1), physiotherapy (*n*=1), and health insurance administration (*n*=1). Interviews conducted between February and March 2024 were analysed using descriptive thematic content analysis and mapped to the Consolidated Framework for Implementation Research.

**Results:**

Six themes emerged: attitudes towards prehabilitation, work environment, healthcare system structure, economic factors, cultural factors, and politics, evidence, and guidelines. Despite unanimous recognition of clinical value, knowledge of prehabilitation varied widely. Critical barriers included the structural and financial separation between inpatient and outpatient sectors, the absence of reimbursement mechanisms, workforce shortages, organisational inertia, and limited advocacy by professional societies. Consolidated Framework for Implementation Research mapping identified barriers across all five domains, with outer setting barriers—particularly fragmented financing and the absence of national guidelines—being the most prominent.

**Conclusions:**

These exploratory findings reveal conceptual, systemic, and cultural barriers to prehabilitation in Austria. The simultaneous absence of integrated financing, professional society advocacy, and coordinated governance may explain Austria’s particularly limited uptake compared to that in the UK and the Netherlands. These findings are hypothesis-generating and require validation through larger-scale qualitative studies, quantitative surveys, and pilot implementation trials that include patient perspectives and economic modelling.

**Editor’s key points**

• Prehabilitation in Austria is valued but not routinely implemented. All interviewed stakeholders recognised its potential clinical benefits, yet knowledge and practical experience with prehabilitation varied substantially.
• Fragmented inpatient–outpatient structures are the central barrier: costs of prehabilitation generally arise in outpatient care, while many benefits—such as fewer complications and shorter hospital stays—accrue to hospitals, creating misaligned incentives.
• Stakeholders identified absent reimbursement mechanisms, workforce shortages, limited space and protected time, siloed working practices, and unclear institutional leadership as major obstacles.
• The study highlights limited advocacy by professional societies, no national guidelines, and insufficient coordinated governance. Future work should involve broader stakeholder groups, including patients, and test pilot programmes with economic evaluation.

Prehabilitation is a multimodal, interdisciplinary intervention designed to optimise a patient’s physiological and psychological condition before major surgery; typically through physical training, nutritional support, and psychological preparation.^1^, ^2^, ^3^, ^4^ Evidence from systematic reviews suggests potential clinical benefits, although certainty remains low to moderate for most outcomes.^5^ Implementation across healthcare systems is heterogeneous: dedicated programmes exist in the UK and the Netherlands, supported by professional bodies and integrated financing structures,^6^, ^7^, ^8^ whereas engagement with prehabilitation in Germany and Austria has been much more limited.

In Austria, there are no national guidelines, no advocacy from professional societies, and only isolated local prehabilitation initiatives for prehabilitation.^9^ Understanding these barriers during the current evidence-building phase is important for designing future pilot programmes and assessing healthcare system readiness. Therefore, this exploratory study aimed to identify stakeholder-perceived barriers to the adoption of prehabilitation as routine perioperative care in Austria and assess the perceived readiness of the healthcare system.

## Methods

### Design and ethics

We conducted an exploratory qualitative study using semi-structured stakeholder interviews and descriptive thematic content analysis, in line with the Standards for Reporting Qualitative Research.^10^ Ethical review requirements were assessed using the official checklist of the Salzburg Regional Ethics Committee (Supplementary Digital Content [SDC] 1); given that the study involved no patients, no vulnerable groups, and no identifiable personal data, a formal ethics submission was not required. The study adhered to the World Medical Association Declaration of Helsinki.^11^

### Sampling

Eight stakeholders were recruited using purposive sampling to maximise professional variation across disciplines essential to prehabilitation delivery: anaesthesiology (*n*=2), surgery (*n*=2), outpatient cardiology (*n*=1), clinical psychology (*n*=1), physiotherapy (*n*=1), and health insurance administration (*n*=1). Following Malterud and colleagues’^12^ information power framework, sampling prioritised breadth across key stakeholder groups over within-group depth, which was appropriate for this exploratory aim. Participant characteristics are presented in Table 1.

**Table 1:** Characteristics of the eight stakeholders interviewed. Interview codes correspond to participant identifiers used throughout the text.

Interview	Specialty	Professional grade	Location of activity	Type of institution	Previous prehabilitation involvement	Further information
A1	Anaesthesia	Head of department	Hospital	Mid-size teaching hospital	Local non-permanent trial	
A2	Anaesthesia	Senior consultant	Hospital	Large teaching hospital	None	Recently retired; previously head of preoperative anaesthetic services
C1	General surgery	Senior consultant	Hospital	Large teaching hospital	None	
C2	General surgery	Experienced trainee	Hospital	Mid-size teaching hospital	Local non-permanent trial	
K1	Cardiology	Specialist cardiologist	Independent practice	Own practice	None	Previously active in rehabilitation medicine
P1	Clinical Psychology	Psychologist	Hospital and independent practice	Small public hospital and own practice	None	
PT1	Physiotherapy	Physiotherapist	Hospital and independent practice	Small public hospital and own practice	None	
S1	Social insurance	Health economist	Social insurance provider	National head office	None	

### Data collection

Interviews conducted between February and March 2024 lasted 29–46 min and were conducted in person (*n*=5) or via video-conferencing (*n*=3) by a master’s thesis candidate (JML), supervised by a senior qualitative researcher (JBE). The semi-structured interview guide (SDC 2) covered four domains: stakeholder perceptions, implementation barriers, healthcare system infrastructure, and innovation readiness.

### Analysis

Audio recordings were transcribed verbatim using Notta.ai (Notta Co, ltd.,Tokyo, Japan) and manually verified by JML. Transcripts were analysed using the thematic content analysis framework of Miles and Huberman,^13^ which involves iterative data reduction, data display, and conclusion drawing, supported by MAXQDA software (VERBI Software, Berlin, Germany). Initial coding was inductive, staying close to participants’ language; pattern codes and themes were developed through constant comparison. Six main themes emerged from this inductive process. Code frequencies were used descriptively to indicate the relative prominence of themes across interviews. To enhance comparability with international implementation research, the emergent themes were subsequently mapped to the five domains of the Consolidated Framework for Implementation Research (CFIR).^14^^,^^15^

### Trustworthiness

Reflexivity was maintained throughout: JML, an Austrian anaesthesiologist, conducted the interviews and led the coding; JBE, a qualitative methodologist with no direct connection to the Austrian context, provided supervision and external critique. MG and ABN contributed to external verification and interpretation.

## Results

Eight semi-structured interviews were analysed. Five interviews were conducted face to face and three via videoconference. Participants represented seven clinically active healthcare professionals and one senior health economist at a national social insurance institution. A detailed description of the participants is presented in Table 1. Full quotes supporting each theme are provided in SDC 3.

### Theme 1: attitudes towards prehabilitation

Knowledge of prehabilitation varied considerably. Only one participant reported being well informed through departmental training and a local pilot. Most had limited or no previous exposure. Nonetheless, perceived clinical value was unanimous: all participants endorsed prehabilitation’s potential to improve surgical outcomes and reduce complications. Some noted specialty-specific scepticism, particularly among surgeons.

### Theme 2: work environment

Personal and physical resources were described as insufficient across settings. Staff shortages, high workloads, and a lack of physical space were commonly cited. Interdisciplinary collaboration and coordination were recognised as essential but were currently absent, with hospitals characterised by siloed working. Unclear leadership responsibility for prehabilitation within institutions was a recurrent concern.

### Theme 3: healthcare system structure

The structural and financial separation between the inpatient and outpatient sectors was identified by all eight participants as a critical barrier. The benefits of prehabilitation (such as reduced length of stay and fewer complications) accrue to hospitals, whereas outpatient providers bear the costs: ‘From the perspective of the cost bearer, hospitals say that these matters, which all arise before the operation, are not their concern’ (S1) Austria’s fragmented hospital governance (with more than 50 operators across nine federal states) further hinders uniform implementation.

### Theme 4: economic factors

The absence of billing codes and reimbursement mechanisms for integrated prehabilitation programmes was unanimously identified as a major structural barrier. Upfront costs and uncertain returns make prehabilitation economically unattractive. The split between inpatient and outpatient financing prevents unified investment and cost–benefit sharing.

### Theme 5: cultural factors

The innovation climate was described as conservative. As one participant summarised: ‘In Austria, we have done it this way for the last 150 yrs, so we will do it (the same way) the next 150 years as well’. (PT1) Patient and public attitudes were perceived by clinicians as variable, although these assessments represent professional perceptions rather than empirically validated patient views.

### Theme 6: politics, evidence, and guidelines

Political engagement was described as reactive and evidence contingent. Robust cost-effectiveness data and national guidelines were seen as prerequisites for political support. The limited advocacy by Austrian medical societies was viewed as a key obstacle: ‘In the UK, the anaesthetic society will have pushed this, and it was taken up by anaesthetists across the whole country. Something like this hasn’t happened here’. (A1)

### Profession-specific patterns

The health insurance representative (S1) focused almost exclusively on economic and reimbursement barriers, therapists (PT1, P1) emphasised cultural and patient-related factors, and hospital-based physicians (A1, A2, C1, C2) highlighted system-level and organisational barriers. Two factors were consistently raised by all eight participants: the inpatient/outpatient financing interface and national-level economic constraints. Coordination challenges and the lack of interdisciplinary collaboration were raised by seven of the eight participants. Detailed code frequency patterns by participant are illustrated in Supplementary Figure 1 (SDC 4).

Barriers were identified across all five CFIR domains (Table 2). Outer setting barriers; fragmented financing, absent reimbursement, lack of national guidelines, and inactive professional societies, were identified as the most prominent obstacles. The implementation process domain was notable for its complete absence in the Austrian context.

**Table 2:** Mapping of emergent themes to the five domains of the Consolidated Framework for Implementation Research (CFIR). Findings reflect barriers and, where relevant, facilitators identified across all eight interviews.

CFIR domain	Key findings
Innovation	Variable understanding of prehabilitation scope and components across disciplines; perceived evidence uncertainty
Outer setting	Inpatient/outpatient financing fragmentation; absence of reimbursement codes; lack of national guidelines; inactivity of medical societies; political demand for cost-effectiveness evidence
Inner setting	Workforce shortages; limited physical space; high workloads; absence of protected non-clinical time; organisational inertia; conservative institutional culture
Individuals	Positive attitudes but variable knowledge; motivation constrained by workload; perceived (but unvalidated) patient non-compliance
Implementation process	No formal implementation strategies in place; absence of interdisciplinary leadership; unclear role allocation across providers

## Discussion

This exploratory qualitative study identified several potential barriers to the adoption of prehabilitation in Austria, based on perspectives from eight stakeholders with diverse professional backgrounds. Despite unanimous recognition of its clinical value, all five CFIR domains contained barriers, suggesting that progress would require coordinated multilevel action rather than isolated interventions.

Structural fragmentation between the inpatient and outpatient sectors, which operate with minimal communication and misaligned financial incentives, was the most consistently identified barrier. This reflects established features of the Austrian healthcare system.^16^^,^^17^ The absence of reimbursement mechanisms for integrated preoperative programmes has been reported internationally as a critical implementation barrier,^18^^,^^19^ and without a clear reimbursement pathway, prehabilitation remains economically unattractive to stakeholders, potentially perpetuating a cycle of inaction. Whether establishing reimbursement structures would overcome this barrier requires testing through funded pilot programmes with embedded economic evaluation.

Comparison with countries that have made greater progress is instructive. In the UK, the integrated structure of the NHS enables cross-sectoral coordination without separate funding negotiations, and professional bodies—including the Royal College of Anaesthetists and the Centre for Perioperative Care—have actively promoted prehabilitation through national guidance and pilot funding.^6^^,^^7^ In the Netherlands, managed competition within a single insurance system has enabled bundled care pathways spanning multiple sectors.^8^ In Austria, the simultaneous absence of integrated financing, professional society advocacy, and coordinated governance, rather than any single barrier, may explain its particularly limited uptake. This hypothesis requires formal comparative research for empirical validation.

Workforce shortages and organisational inertia were also prominent. Resource constraints (including overburdened staff, limited space, and the absence of protected non-clinical time for programme development) are widely reported as implementation barriers across healthcare systems.^20^^,^^21^

Several limitations must be acknowledged. The small purposive sample (*n*=8) ensures professional diversity but limits representativeness within any single discipline and does not support statistical generalisation. Groups absent from the sample include general practitioners, dedicated policy makers, information technology specialists, and patients; groups likely to contribute additional perspectives, particularly on primary care interfaces and patient-centred barriers. Perceptions of patient motivation and compliance were reported exclusively from the clinician perspective and require validation with direct patient input. The data reflect a specific snapshot (February to March 2024) and may not represent all Austrian regions or healthcare operators.

These findings are hypothesis-generating. The convergence of themes across diverse stakeholder groups suggests structural patterns worth investigating, but validation through larger-scale qualitative studies, quantitative surveys of broader stakeholder populations, and pilot implementation trials with economic modelling is required before these findings can inform national implementation strategies.

## Conclusion

Despite unanimous stakeholder support for prehabilitation’s clinical value, this exploratory study identified the structural separation between inpatient and outpatient care, the absence of reimbursement mechanisms, and limited professional society advocacy as the most prominent barriers to implementation in Austria. Consolidated Framework for Implementation Research mapping confirms that barriers span all system levels. Future research should validate these findings with larger, more diverse samples, including patients, and incorporate pilot programmes with economic evaluation to move from barrier identification to evidence-based implementation strategies.

## Authors’ contributions

Conceived and designed the study: JML, JBE

Conducted the interviews, transcribed the data, and led the qualitative analysis under supervision: JML

Provided methodological oversight, contributed to development of the interview guide, supported data interpretation, and supervised the analysis: JBE

Contributed to the interpretation of findings and provided critical intellectual input: MG, ABN

Contributed to drafting and revision of the manuscript and approved the final version: all authors

## Declaration of generative AI and AI-assisted technologies in the writing process

During the preparation of this work, the authors used ChatGPT version 5.2 to improve readability and grammar. After using this tool, the authors reviewed and edited the content as needed and take full responsibility for the published article.

## Declaration of interests

JBE received speakers’ fees from Medtronic and Masimo. She is an associate editor-in-chief of *BJA Open*. The remaining authors declare that they have no conflicts of interest.
