# Supplementary material for: Exploring the barriers to prehabilitation integration in Austrian healthcare: a qualitative analysis
Source: BJA Open. 2026 Oct 6;20:100868. doi: 10.1016/j.bjao.2026.100868 (PMC13675892; doi:10.1016/j.bjao.2026.100868)
Supplement: Supplementary file 1 [file mmc1.docx]

Checkliste zur EK-Pflichtigkeit bei der Landes EK

Version 2 - März 2023

LAND SALZBURG

Ethikkommission

Diese Checkliste soll für alle Forschenden ein Hilfsmittel darstellen, um abzuschätzen zu können, ob ihr Forschungsprojekt bei der Ethikkommission des Bundeslandes Salzburg als gesetzlich legitimierte Ethikkommission für Krankenanstalten im Bundesland Salzburg vorzulegen ist.

1. Handelt es sich um eine reine Literaturarbeit?

   JA ☐ NEIN X

2. Handelt es sich um einen Tierversuch?

   JA ☐ NEIN X

3. Handelt es sich um eine Studie an Leichen?

   JA ☐ NEIN X

Wenn bei einer der Fragen 1-3 JA zutrifft dann entfallen die Fragen 4-14 und das Forschungsprojekt unterliegt nicht der Vorlagepflicht bei der Landesethikkommission. *Bitte beachten Sie jedoch, dass andere gesetzliche Vorlage- oder Genehmigungspflichten vorliegen können.*

4. Werden in dem Forschungsprojekt Arzneimittel am Menschen untersucht?

   JA ☐ NEIN X

5. Werden in dem Forschungsprojekt Medizinprodukte untersucht?

   JA ☐ NEIN X

6. Handelt es sich um eine nicht-interventionelle Studie?

   JA ☐ NEIN X

7. Werden in dem Forschungsprojekt Instrumente, Assays, Reagenzien, Apparate oder andere Materialien systematisch am Menschen zur Diagnose, Verhütung, Überwachung, Vorhersage, Behandlung oder Linderung von Krankheiten, Verletzungen oder physiologischen Zuständen untersucht?

   JA ☐ NEIN X

8. Werden in dem Forschungsprojekt medizinische Software oder Algorithmen für künstliche Intelligenz (KIs) getestet oder trainiert?

   JA ☐ NEIN X

Checkliste zur EK-Pflichtigkeit bei der Landes EK

Version 2 - März 2023

LAND SALZBURG

Ethikkommission

9. Werden in dem Forschungsprojekt Patienten einer Krankenanstalt im Bundesland Salzburg einbezogen?

   JA ☐ NEIN X

10. Werden in dem Forschungsprojekt Daten von Patienten einer Krankenanstalt im Bundesland Salzburg verarbeitet, welche direkt oder indirekt identifizierbar sind?

   JA ☐ NEIN X

11. Wird in dem Forschungsprojekt biologisches Material menschlichen Ursprungs verwendet, welches von Patienten einer Krankenanstalt im Bundesland kommt?

   JA ☐ NEIN X

12. Werden im Rahmen des Forschungsprojektes medizinische Interventionen (Behandlungen, Blutabnahmen, Fragebögen, Bildgebung, etc.) vorgenommen, welche ärztliche Mitwirkung oder Aufsicht erfordern?

   JA ☐ NEIN X

13. Werden nicht entscheidungsfähige Personen einbezogen und wird die körperliche Unversehrtheit oder die Persönlichkeit dieser Personen beeinträchtigt?

   JA ☐ NEIN X

14. Handelt es sich um die Einrichtung eines Forschungsregisters oder die Auswertung eines bestehenden Registers (Registerstudie) an einer Krankenanstalt im Bundesland Salzburg?

   JA ☐ NEIN X

Wurde eine der Fragen 4-14 mit JA beantwortet, liegt voraussichtlich eine Vorlagepflicht bei der Ethikkommission des Landes Salzburg vor.

Sollten eine der Fragen 9-14 für Patienten in anderen Bundesländern zutreffen, ist eine Vorlagepflicht bei den dortigen, örtlich zuständigen, gesetzlich legitimierten Ethikkommissionen zu klären.
