# Supplementary material for: Exploring the barriers to prehabilitation integration in Austrian healthcare: a qualitative analysis
Source: BJA Open. 2026 Oct 6;20:100868. doi: 10.1016/j.bjao.2026.100868 (PMC13675892; doi:10.1016/j.bjao.2026.100868)
Supplement: Supplementary file 2 [file mmc2.docx]

**Interview Guide: “Prehabilitation in Austria” (30–60 minutes)**

**Introduction**

- Welcome the interview participants, introduce the interviewer, and explain the purpose of the study
- Confirm consent to participate and to audio-record the interview
- Provide a brief overview of the interview structure

**Background Information**

- Could you describe your professional role and experience in the healthcare sector?
- How familiar are you with the concept of prehabilitation, particularly in the context of major surgical procedures?

**Perception and Use of Prehabilitation**

- How do you perceive the concept of prehabilitation in Austria, particularly regarding its relevance and benefits?
- Has prehabilitation been discussed or already implemented in your professional work environment? Did you consider this useful?
- Based on your experience, how is prehabilitation currently being used in Austria for patients undergoing major surgical interventions?

**Barriers to Implementation**

- If you were to establish a prehabilitation programme in your work environment, how feasible would this be?
- Which factors in your personal work environment hinder the implementation of prehabilitation (e.g. workload, personal resources)?
- Which factors hinder the implementation of prehabilitation at an institutional level (e.g. funding, interdisciplinary coordination)?
- What problems or barriers prevent the widespread implementation of prehabilitation at a national level in Austria?
- What would need to change to facilitate the implementation of prehabilitation?
- Can you compare these challenges with other countries where prehabilitation has been more widely adopted?

**Structure of the Healthcare System and Resources**

- In your opinion, how do the current structures and processes of the Austrian healthcare system support or hinder the implementation of prehabilitation programmes?
- Are there specific financial, organisational, or structural factors that influence the implementation of prehabilitation?

**Readiness for Innovation and Change**

- How would you assess the readiness for innovation and change within the Austrian healthcare system, particularly at the micro- and meso-levels?
- Which specific factors influence the healthcare system’s willingness to adopt innovative practices such as prehabilitation?

**Comparative Analysis and Future Perspectives**

- How innovative is the Austrian healthcare system compared with other countries?
- How does the Austrian approach to prehabilitation differ from that of other countries where it is already well established, taking into account cultural, organisational, and financial aspects?
- Based on your experience, what recommendations would you give to promote the implementation of prehabilitation within the Austrian healthcare system?

**Closing**

- Do you have any additional comments or thoughts you would like to share on this topic?
- Thank the interview participants for their time and participation
