# Supplementary material for: Exploring the barriers to prehabilitation integration in Austrian healthcare: a qualitative analysis
Source: BJA Open. 2026 Oct 6;20:100868. doi: 10.1016/j.bjao.2026.100868 (PMC13675892; doi:10.1016/j.bjao.2026.100868)
Supplement: Supplementary file 3 [file mmc3.docx]

15.07.2025

Table of contents

[1. Documents	1](#_Toc203505196)

[2. Organisation Arbeitsumfeld	1](#_Toc203505197)

[2.1. Persönliche Ressourcen	1](#_Toc203505198)

[2.2. Koordinierung	1](#_Toc203505199)

[2.3. Interdisziplinäre Zusammenarbeit	1](#_Toc203505200)

[2.4. Planbarkeit der Prähabilitation	1](#_Toc203505201)

[3. Struktur des Gesundheitswesens	1](#_Toc203505202)

[3.1. Vorhandensein ambulante Strukturen	1](#_Toc203505203)

[3.2. Schnittstelle Intra- / Extramural	1](#_Toc203505204)

[3.3. Unterschiedliche Träger	1](#_Toc203505205)

[4. Wirtschaftliche Faktoren	1](#_Toc203505206)

[4.1. Wirtschaftliche Faktoren Land / Bund / Kasse	1](#_Toc203505207)

[4.2. Wirtschaftliche Faktoren KH	1](#_Toc203505208)

[5. Kulturelle Faktoren	1](#_Toc203505209)

[5.1. Innovationsklima	1](#_Toc203505210)

[5.2. Politik	1](#_Toc203505211)

[5.3. Einstellung Bevölkerung / Patienten	1](#_Toc203505212)

[6. Einstellung zur Prähabilitation	1](#_Toc203505213)

[6.1. Sinnhaftigkeit der Prähabilitation	1](#_Toc203505214)

[6.2. Motivation	1](#_Toc203505215)

[6.3. Kenntnis der Prähabilitation	1](#_Toc203505216)

[7. Guidelines, Empfehlungen	1](#_Toc203505217)

[7.1. Einheitliches Konzept, Evidenz	1](#_Toc203505218)

[7.2. Fachgesellschaften, Organisationen	1](#_Toc203505219)

1. Documents

| No. | Document | Created by |
| --- | --- | --- |
| 1 | S1 080324 | Jan |
| 2 | PT1 140324 | Jan |
| 3 | P1 010324 | Jan |
| 4 | K1 280224 | jmlut |
| 5 | A1 210224 | jmlut |
| 6 | A2 220422 | jmlut |
| 7 | C1 200224 | jmlut |
| 8 | C2 180324 | Jan |

2. Organisation Arbeitsumfeld

2.1. Persönliche Ressourcen

1.

"Also wenn das jetzt regelkonform so wäre, dann sind ja auch viele Operationen,
große. Also ich denke mal, da muss man auf alle Fälle noch mindestens,
wahrscheinlich noch einmal eine Halbtagsstelle ausschreiben."

*“So if that were to be done in accordance with the rules, then there would also be many operations, large ones. So I think that in any case, you would have to advertise at least*

*another half-day position."*

Code: ● Persönliche Ressourcen	Weight score: 0

P1 010324, 111 - 113

Created: 20.05.2024 18:27 by Jan, Modified: 20.05.2024 18:27 by Jan

Area: 212	0.55%

2.

"Also auch das Platzproblem ist ein wesentliches. Das heißt, hier noch Platz mehr
zu finden für zusätzliche Leistungen ist oft ganz schwierig. Die Verwaltung ist
da im Prinzip dann sozusagen dahintergelegt, weil wenn sozusagen diese Teams,
die ich da angesprochen habe, sagen wir mal die Chirurgie und die Physiotherapie,
 die Diätologen, die sich finden, die auch sonst intensiv zusammenarbeiten."

*"So the space problem is also a significant one. That means it is often very difficult to find more space here for additional services. In principle, the administration is behind this, so to speak, because when the teams I mentioned, let's say surgery and physiotherapy,the dieticians, who work together intensively in other areas as well, get together."*

Code: ● Persönliche Ressourcen	Weight score: 0

A1 210224, 262 - 266

Created: 09.05.2024 17:25 by jmlut, Modified: 09.05.2024 17:25 by jmlut

Area: 399	1.00%

3.

"Und was jetzt auch noch dazu kommen wird und in den nächsten Jahren verstärkt,
ist Personalmangel und zwar in jeder Hinsicht, wo auch Menschen abgehen, gerade
in der Pflege, die sehr nah am Patienten sind und da viel machen können, aber
auch bei Ärzten und Physiotherapeuten."

*"And what will now be added to this and intensify in the coming years is a shortage of personnel in every respect, with people leaving, especially in nursing, where they are very close to patients and can do a lot, but also among doctors and physiotherapists."*

Code: ● Persönliche Ressourcen	Weight score: 0

A2 220422, 206 - 209

Created: 09.05.2024 19:40 by jmlut, Modified: 09.05.2024 19:40 by jmlut

Area: 278	0.78%

4.

"Ja, natürlich geht es ums Geld und es geht tatsächlich ja darum, dass man um
neue Strukturen oder neue Projekte zu implementieren, motivierte und engagierte
Mitarbeiter braucht. Und es ist sehr einfach, in der derzeitigen Situation ist
es einfach so angespannt, dass die Kolleginnen und Kollegen nicht mehr so bereit
sind, von ihrer Freizeit sehr viel in die Arbeit zu investieren, sagen wir es
einmal ganz vorsichtig. Nicht weil sie unendlich faul sind, sondern weil da irgendetwas fehlt an Anreiz."

*"Yes, of course it's about money, and it's actually about the fact that you need motivated and committed employees to implement new structures or new projects. And it's very easy, in the current situation, which is so tense that colleagues are no longer so willing to invest a lot of their free time in work, to put it very cautiously. Not because they are infinitely lazy, but because there is something missing in terms of incentive."*

Code: ● Persönliche Ressourcen	Weight score: 0

A2 220422, 571 - 578

Created: 09.05.2024 19:52 by jmlut, Modified: 09.05.2024 19:52 by jmlut

Area: 522	1.46%

5.

"Ja, das sehe ich schon so. Dass die dies erwarten, dass man alles, was jetzt
nicht in der gewohnten Versorgungsroutine drinnen ist oder in unsere Neue zu
projektieren ist, bitte schön nicht in der regulären Arbeitszeit machen. Und da, das ist sicher eine Riesenschere, weil die Kolleginnen und Kollegen und aus dem pflegenden Berufen, das ist alles miteinander, die sind nicht mehr bereit, da 300 Überstunden zu leisten im Monat."

"*Yes, I see it that way too. That they expect us not to do anything that is not part of the usual care routine or that is not included in our new plans during regular working hours. And there, that's definitely a huge gap, because our colleagues And that's certainly a huge gap, because our colleagues in the nursing professions, all of them, are no longer willing to work 300 hours of overtime per month."*

Code: ● Persönliche Ressourcen	Weight score: 0

A2 220422, 587 - 593

Created: 09.05.2024 19:53 by jmlut, Modified: 09.05.2024 19:53 by jmlut

Area: 451	1.26%

6.

"Teamsitzungen gehören unbedingt wieder eingeführt, verpflichtend, nicht freiwillig. Gehören eingeführt, einmal im Monat, dass man sie besprechen kann im Team mit allen Professionalitäten, die an dieser Abteilung arbeiten, das ist ja unterschiedlich, aber das gehört unbedingt wieder eingeführt. Und die Bereitschaft zu sagen, wer hat eine Idee, gibt es eine neue Struktur, die wir aufbauen sollen, gibt es etwas, was wir verbessern können."

*‘Team meetings must be reintroduced, mandatory, not voluntary. They should be introduced once a month so that they can be discussed within the team with all the professionals working in this department. It varies, but it must be reintroduced. And the willingness to say, who has an idea, is there a new structure we should build, is there something we can improve?’*

Code: ● Persönliche Ressourcen	Weight score: 0

A2 220422, 639 - 646

Created: 09.05.2024 19:54 by jmlut, Modified: 09.05.2024 19:54 by jmlut

Area: 462	1.30%

7.

"die Antwort, die ich dann bekomme - ja, wir sind für den stationären Bereich zuständig und können den Patienten ja dann nicht die ganze Zeit betreuen. Dafür fehlt uns die Manpower, dafür fehlt uns die Zeit und dafür haben wir nicht die Ressourcen. Das nimmt man dann zur Kenntnis, weil es ist ja nicht meine Abteilung."

*‘The answer I get is, “Yes, we are responsible for the inpatient area and cannot look after patients all the time. We don't have the manpower, we don't have the time, and we don't have the resources.” You accept that because it's not your department.’*

Code: ● Persönliche Ressourcen	Weight score: 0

C1 200224, 166 - 172

Created: 01.05.2024 16:21 by jmlut, Modified: 01.05.2024 16:21 by jmlut

Area: 340	0.77%

8.

"Da sind wir weit weg davon, dass die da irgendwie trainieren oder mit ihnen
körperlich das verbessern und so weiter. Und auch da auf Nachfragen, warum,
wieso geht das nicht: ja dafür haben wir nicht die Ressourcen, dafür sind wir
nicht da."

*‘We are far from training them or improving their physical abilities and so on. And when asked why this is not possible, we respond that we do not have the resources for this and that this is not our purpose.’*

Code: ● Persönliche Ressourcen	Weight score: 0

C1 200224, 185 - 188

Created: 01.05.2024 16:24 by jmlut, Modified: 01.05.2024 16:24 by jmlut

Area: 242	0.55%

9.

"Das Erkennen, dass der Patient Ernährung braucht, das wird gesehen, nur er hat
keine Person sozusagen, die sich dann kontinuierlich um ihn kümmert und sagt,
okay, die Eiseninfusion wird gemacht beim Hausarzt eben und ich schau darauf,
dass sie das bekommen haben und dann wird wieder kontrolliert und so weiter,
sondern - oder die Ernährung wird monitorisiert und wer dann Ansprechpartner
eben, wo das gemacht wird, dazu fehlt die Zeit in der normalen Ambulanz eben,
sondern wir können uns sagen, okay."

*"Recognising that the patient needs nutrition is obvious, but he doesn't have*

*anyone, so to speak, who continuously looks after him and says, 'OK, the iron infusion will be done by the family doctor and I'll make sure that you get it, and then it will be checked again and so on', but rather – or the nutrition is monitored and who is the contact person*

*where this is done, there is simply not enough time in the normal outpatient clinic,*

*but we can say to ourselves, okay."*

Code: ● Persönliche Ressourcen	Weight score: 0

C1 200224, 219 - 225

Created: 01.05.2024 16:30 by jmlut, Modified: 01.05.2024 16:30 by jmlut

Area: 508	1.16%

10.

"Das Problem ist das, du hast dann diesen einen Patienten und dann hast du diesen
einen Tag, von mir aus, wenn ich da eine Sprechstunde mache, dann hast du zehn
von denen gesehen. Dann siehst du zwar die Befunde, aber eigentlich müsstest du
dann jeden dieser Patienten nachverfolgen, wie geht's dem denn jetzt unter der
Chemotherapie, bis er wieder zu mir kommt, präoperativ, und macht jetzt seine
Übungen, hat der sein Eisen gekriegt, ist der Hb okay, ist der vom
Ernährungsstatus usw. in Ordnung. Das pack ich nicht während der normalen Arbeit, weil in dem Moment, wo die Ambulanz aus ist, geht das Ganze weiter eben und du operierst, dann machst du die anderen Tätigkeiten, Visite etc. und dann nächste Woche kommen die nächsten."

*"The problem is that you have this one patient, and then you have this*

*one day, let's say when I'm doing a consultation, and then you've seen ten*

*of them. Then you see the findings, but you actually have to follow up on each of these patients, how are they doing now under chemotherapy, until they come back to me, preoperatively, and are now doing their exercises, have they got their iron, is their Hb okay, is their nutritional status okay, etc. I can't manage that during normal work, because the moment the outpatient clinic is over, the whole thing continues and you operate, then you do the other activities, rounds, etc., and then next week the next ones come in."*

Code: ● Persönliche Ressourcen	Weight score: 0

C1 200224, 236 - 246

Created: 01.05.2024 16:31 by jmlut, Modified: 01.05.2024 16:31 by jmlut

Area: 757	1.72%

11.

"Also ich kenne mich aus im stationären Bereich. Das heißt, ich kann, wenn ich
der Meinung bin, dass der Physio und also präoperative Optimierung braucht, dann
kann ich nur hier drinnen zuweisen. Und die hier drinnern sagen mir ich hab
eigentlich auch nicht die Ressourcen für das."

*"So, I know my way around the inpatient sector. That means that if I*

*think that the patient needs physiotherapy and preoperative optimisation, then*

*I can only refer them here. And the people here tell me that*

*they don't really have the resources for that either."*

Code: ● Persönliche Ressourcen	Weight score: 0

C1 200224, 268 - 271

Created: 01.05.2024 16:33 by jmlut, Modified: 01.05.2024 16:33 by jmlut

Area: 283	0.64%

12.

"Aber dann ist es so, dass das dann genau an den Dingen, die ich vorher schon
gesagt habe, meistens scheitert eben. Du nimmst dann Kontakt auf mit all jenen
Stellen, die da eigentlich dazugehören würden, und du kriegst dann immer die
gleichen Antworten. Ja, wer soll das machen eben? Ich habe weder die Leute dafür. Wir sind jetzt schon am Anschlag, wir können nicht mehr. Und diese Betreuung können wir hier im
stationären Bereich nicht anbieten. Und ambulant machen wir nicht so irgendwie."

*"But then it's the case that it usually fails precisely because of the things I mentioned earlier.*

*You contact all the relevant agencies, and you always get the same answers. Yes, who is supposed to do that? I don't have the people for it. We're already at our limit, we can't do any more. And we can't offer this kind of support here in the inpatient ward. And we can't do it on an outpatient basis either.Yes, who is supposed to do that? I don't have the people for it. We are already at our limit, we can't do any more. And we can't offer this kind of care here in the inpatient ward. And we don't do it on an outpatient basis either."*

Code: ● Persönliche Ressourcen	Weight score: 0

C1 200224, 385 - 392

Created: 01.05.2024 16:38 by jmlut, Modified: 01.05.2024 16:38 by jmlut

Area: 513	1.17%

13.

"Ich habe keine Ahnung, ob du dann zum Beispiel als Diätberaterin eben - ist zum
Beispiel das Feedback, was ich von denen bekomme, ist, ich kann einmal sprechen
mit denen und aus. Aber die kann die jetzt nicht, quasi, während der drei Monate
von der Diagnosestellung bis zur OP begleiten, sondern die reden einmal mit
denen, das war die Zuweisung und aus."

*"I have no idea if you, as a dietitian, for example – the feedback I get from them is that I can talk to them once and that's it. But they can't accompany them during the three months*

*from diagnosis to surgery, they just talk to them once and that's it."*

Code: ● Persönliche Ressourcen	Weight score: 0

C1 200224, 571 - 575

Created: 01.05.2024 21:36 by jmlut, Modified: 01.05.2024 21:36 by jmlut

Area: 358	0.81%

14.

"Das ist zwar ein super Projekt oder ein super Ansatz. Aber wenn das eigentliche
Problem ist, dass man die Grundversorgung schon nicht hinkriegt, weil man mit
den handelnden Personen quasi nicht zu Rande kommt, weil es zu wenige sind, dann
wird es nicht funktionieren, dass man sich mit anderen Dingen beschäftigt.
Das kann vielleicht ein Grundproblem sein, wenn wir ja wissen, dass periphere
Häuser immer schwerer besetzbar sind und z .T. einfach das Personal für gewisse
Dinge nicht da ist."

*"It's a great project or a great approach. But if the real problem is that you can't even manage basic care because you can't cope with the people involved because there are too few of them, then it's not going to work to focus on other things.*

*This may be a fundamental problem, given that we know that peripheral facilities are becoming increasingly difficult to staff and that, in some cases, there is simply not enough personnel for certain tasks.*

Code: ● Persönliche Ressourcen	Weight score: 0

C2 180324, 492 - 499

Created: 20.05.2024 21:39 by Jan, Modified: 20.05.2024 21:39 by Jan

Area: 515	1.51%

2.2. Koordinierung

1.

"also irgendjemand muss halt den Fall führen aus meiner Sicht und dem
Patienten das Portfolio dann aufspannen und mit ihm das durchgehen. Und dann
werden da wahrscheinlich, also wenn man sich den Versorgungsprozess dann
überlegt, ist es ja so, dass in den Krankenhäusern selbst natürlich auch immer
Gesundheitsberufe da sind, DiätologInnen und PhysiotherapeutInnen und so."

*"So, in my opinion, someone has to take charge of the case and*

*then open up the portfolio for the patient and go through it with them. And then*

*there will probably be, well, if you think about the care process,*

*it's the case that in hospitals themselves, of course, there are always*

*healthcare professionals there, dieticians and physiotherapists and so on."*

Code: ● Koordinierung	Weight score: 0

S1 080324, 217 - 221

Created: 20.05.2024 17:06 by Jan, Modified: 20.05.2024 17:06 by Jan

Area: 375	1.44%

2.

"jeder hat so seine eigene Geschichte laufen und ja, also ich finde die
Idee extrem cool, aber es ist halt, wie gesagt, man muss halt irgendeine
Struktur schaffen, wo das alles zusammenläuft und dann glaube ich, wird das auch
auf fruchtbaren Boden treffen, aber sonst..."

*"Everyone has their own story going on, and yes, I think the*

*idea is extremely cool, but it's just, as I said, you have to create some kind of*

*structure where everything comes together, and then I think it will*

*fall on fertile ground, but otherwise..."*

Code: ● Koordinierung	Weight score: 0

PT1 140324, 282 - 285

Created: 20.05.2024 20:06 by Jan, Modified: 20.05.2024 20:06 by Jan

Area: 272	0.76%

3.

"Wo das dann alles zusammenläuft und wo du dann nachher alles beinander
hast, dann nützt es ja was. Die Situation ist total klasse, wenn ich weiß, ich
habe jetzt von dem Herrn So und So eine Zuweisung, der sagt mir dann, das ist
das Hauptproblem in seinen Augen, dann schauen wir das an und sagen, ja, ich
stimm dir zu, oder ich glaube, dass da vielleicht das sein kann."

*"When everything comes together and you have everything in one place, then it's useful. The situation is great when I know that I have a referral from Mr So-and-so, who tells me that this is the main problem in his eyes, then we look at it and say, yes, I agree with you, or I think that might be the case."*

Code: ● Koordinierung	Weight score: 0

PT1 140324, 289 - 293

Created: 20.05.2024 20:06 by Jan, Modified: 20.05.2024 20:06 by Jan

Area: 373	1.05%

4.

"ich glaube schon, dass das von der Ärztewelt ausgehen muss, das die das
initiieren,"

*‘I do believe that it has to come from the medical community, that they have to initiate it.’*

Code: ● Koordinierung	Weight score: 0

PT1 140324, 324 - 325

Created: 20.05.2024 20:08 by Jan, Modified: 20.05.2024 20:08 by Jan

Area: 84	0.24%

5.

"Ich habe gesehen, dass die Organisation und die Zeiteinteilung für den Patienten
vor der Operation auch schon etwas sehr, sehr Schwieriges ist. Überhaupt wenn er
psychisch jetzt so alteriert ist. Das heißt, man müsste ihm schon einen
vorgegebenen Plan zur Verfügung stellen und dieser Plan - dass man das
organisatorisch gut aufsetzt, da braucht man schon ein gewisses
Organisationstalent und auch die passende EDV dazu und das ist etwas, was glaube
ich in den ambulanten Rehabilitationszentren, wo man ja gewohnt ist,
verschiedene Berufsgruppen zu koordinieren, dass das dort einfach schon gut
aufgestellt ist und dass dort auch schon stattfindet."

*"I have seen that the organisation and timing for the patient before the operation is also something very, very difficult. Especially when he is now so mentally altered. That means you would have to provide him with a predetermined plan, and this plan – to set it up well in terms of organisation – requires a certain amount of organisational talent and the appropriate IT, and that is something that I believe organisational skills and the appropriate IT, and that is something that I believe is already well established in outpatient rehabilitation centres, where they are used to coordinating different professional groups, and that it is already happening there."*

Code: ● Koordinierung	Weight score: 0

K1 280224, 286 - 294

Created: 09.05.2024 20:30 by jmlut, Modified: 09.05.2024 20:30 by jmlut

Area: 656	1.77%

6.

"Was nicht funktioniert hat, kann ich Ihnen vielleicht auch noch aus meiner Erfahrung
sagen: die ambulante neurologische Rehabilitation wurde am Anfang so organisiert,
 dass eben die Patienten - das es zwar von der Versicherung bezahlt wurde, aber
sie mussten zu einem extra Psychologen, sie mussten ihre Termine bei der
Ergotherapeutin extra ausmachen, sie mussten den Termin beim Physiotherapeutin
extra ausmachen, sie mussten dann wieder beim Orthopägen oder beim Neurologen
den Termin extra ausmachen, auch im ambulanten Setting, da verliert.sich das
ganze, Sondern wenn, dann sollte das wirklich zentriert, die Organisation in
einer Hand sein und die Leute an eine Stelle kommen, also entweder, sei es ins
Krankenhaus, sei es in ein ambulantes Rehaszentrum, sei es in ein
Versorgungszentrum, sei es auch in Internisten oder Ordinationsgemeinschaften,
die das auch anbieten wollen, wie auch immer."

*"I can also tell you from my own experience what didn't work: outpatient neurological rehabilitation was initially organised in such a way that patients – although it was paid for by their insurance – had to see a psychologist separately, make separate appointments with the*

*occupational therapist, and make separate appointments with the physiotherapist, they had to make separate appointments with the orthopaedist or neurologist, also in an outpatient setting, the whole thing gets lost. Instead, it should really be centralised, with the organisation in one place and people coming to one location, whether it be the hospital, an outpatient rehabilitation centre, a care centre, or even internists or group practices*

*that also want to offer this, whatever the case may be."*

Code: ● Koordinierung	Weight score: 0

K1 280224, 608 - 620

Created: 09.05.2024 20:43 by jmlut, Modified: 09.05.2024 20:43 by jmlut

Area: 911	2.45%

7.

"Das heißt, die Slots in der Ambulanz dann freimachen, wo man diese Patienten
noch einmal zusätzlich sieht. Das ist in einem Krankenhaus, wo Platzmangel und
Terminenge schon sehr hoch ist, gar nicht so einfach. Das heißt, das muss man dann halt zwischen den Leitern, den motivierten Leitern und Mitarbeiten, also Leitern absprechen, die motivierte Mitarbeiter halt haben, dass man diese Patienten überhaupt unterbringt."

*"That means freeing up slots in the outpatient clinic, where these patients are seen*

*again. That's not so easy in a hospital where space is limited and*

*appointments are already very tight. That means you have to discuss it between the managers, the motivated managers and employees, i.e. managers who have motivated employees, so that these patients can be accommodated at all."*

Code: ● Koordinierung	Weight score: 0

A1 210224, 172 - 178

Created: 09.05.2024 17:17 by jmlut, Modified: 09.05.2024 17:17 by jmlut

Area: 440	1.11%

8.

"Und man muss ja auch sagen, es muss ja dann auch zum Haus dazu passen. Es muss
ja auch lokal umsetzbar sein."

*"And you also have to say that it has to suit the full house (hospital). It has to be locally feasible."*

Code: ● Koordinierung	Weight score: 0

A1 210224, 205 - 206

Created: 09.05.2024 17:21 by jmlut, Modified: 09.05.2024 17:21 by jmlut

Area: 109	0.27%

9.

"Dann ein ganz wichtiger Punkt, auch der Arbeitsplatz, also wirklich, welche
Ambulanzen können dafür freigestellt sein. Bei uns sind die Ambulanzen so dicht
getacktet in der Regel, dass es in der Regel gar keinen freien Raum gibt, wo man
noch zusätzlich etwas machen könnte."

*"Then there's another very important point, namely the workplace, i.e. which*

*ambulances can be exempted for this purpose. In our case, the ambulances are so tightly*

*scheduled that there is usually no free space where you could do anything extra."*

Code: ● Koordinierung	Weight score: 0

A1 210224, 257 - 260

Created: 09.05.2024 17:24 by jmlut, Modified: 09.05.2024 17:24 by jmlut

Area: 276	0.69%

10.

"Machbar glaube ich, ist es unbedingt, ja, aber ich glaube wirklich, da braucht
es eine Person, die sozusagen der Kopf dieses Ganzen ist und der nur für die
Aufgabe freigestellt ist. Er müsste wahrscheinlich, oder sie, sogar, nehmen wir
jetzt halt das Land [entfernt] her, die einzelnen Häuser vielleicht bereisen,
das Modell vorstellen und diskutieren, dort wieder so ein Head, einen Kollegen
eruieren, der sich mit dem auseinandersetzt, der am Haus, an dem er ja arbeitet
und dann, wenn das passiert ist, mit den Einzelstationen, dass man sich trifft
und sagt, wir können das zusammenführen."

*"I think it's definitely feasible, yes, but I really believe that it needs a person who is, so to speak, the head of the whole thing and who is only assigned to this task. He or she would probably have to, let's take the country [removed] as an example, visit the individual houses,*

*present the model and discuss it, find a colleague there who is familiar with the house he is working on and then, once that has happened, meet with the individual stations and say, we can bring this together."*

Code: ● Koordinierung	Weight score: 0

A2 220422, 162 - 169

Created: 09.05.2024 19:39 by jmlut, Modified: 09.05.2024 19:39 by jmlut

Area: 599	1.68%

11.

"Wahrscheinlich ist da Digitalisierung auch von Vorteil, weil man da möglich gute
Schnittstellen schaffen kann, wo man sich unkompliziert austauschen kann.
Möglich ja, aber mit einem großen Energie- und Kraftaufwand verbunden."

*"Digitalisation is probably also an advantage here, because it allows you to create good*

*interfaces where you can exchange information easily. It's possible, yes, but it requires a lot of energy and effort."*

Code: ● Koordinierung	Weight score: 0

A2 220422, 171 - 173

Created: 09.05.2024 19:39 by jmlut, Modified: 09.05.2024 19:39 by jmlut

Area: 227	0.64%

12.

"Ja, habe ich mal erlebt, sogar in dem Haus, wo ich gearbeitet habe, das war
sogar im Rahmen einer präoperativen Optimierung. Da wurde eine Stelle geschaffen,
 sozusagen als Koordinator, ja, ein einziges Mal in 30 Jahren."

*"Yes, I've seen it happen, even in the hospital where I worked, as part of a preoperative optimisation programme. A position was created, as a coordinator, so to speak, yes, just once in 30 years."*

Code: ● Koordinierung	Weight score: 0

A2 220422, 470 - 472

Created: 09.05.2024 19:49 by jmlut, Modified: 09.05.2024 19:49 by jmlut

Area: 221	0.62%

13.

"Wenn man alte Krankenhäuser sieht, dann sind die immer noch, und das sehen wir
auch hier, immer noch so aufgebaut, dass es ganz viele kleine Einheiten gibt, wo
jeder vor sich hinwerkelt. Und es ist extrem schwer, so ein Projekt aufzubauen,
wo ganz viele Leute aus verschiedensten Bereichen zusammenkommen."

*"When you look at old hospitals, they are still, as we see here, structured in such a way that there are lots of small units where everyone works on their own. And it is extremely difficult to set up a project like this, where lots of people from different areas come together."*

Code: ● Koordinierung	Weight score: 0

A2 220422, 611 - 614

Created: 09.05.2024 19:53 by jmlut, Modified: 09.05.2024 19:53 by jmlut

Area: 308	0.86%

14.

"Das Problem ist, die kriegen dann vielleicht in Rahmen dessen ein Gespräch...
...aber niemand monitort das. Also das ist den Onkologen vielleicht nicht so
wichtig, die schauen auf andere Dinge. Wir haben uns auf der Abteilung selbst
schon damit beschäftigt, wie der Patient zur Operation kommt, haben sogar jetzt
Studien gemacht darüber, wir haben jetzt gerade wieder eingereicht zum Kongress.
die sogenannte BIA-Messung, also das ist eine Messung, wo du die
Körperzusammensetzung messen kannst, oder wir haben auch gemeinsam mit den
Onkologen dann bei den Tumoren geschaut, wie die Körperzusammensetzung beim CT
ist, also Fett und Muskelmasse und ob das dann Auswirkungen hate auf das
operative Outcome."

*"The problem is that they might get a consultation within that framework...*

*...but no one is monitoring that. So that may not be so important to the oncologists ,*

*they look at other things. We have already looked into this ourselves in the department ,*

*how the patient comes to the operation, we have even done studies on it, we have just submitted them again to the congress.*

*The so-called BIA measurement, which is a measurement where you can measure body composition, or we also worked with the oncologists to look at tumours and see what the body composition is like on the CT scan , i.e. fat and muscle mass, and whether that then has an impact on the surgical outcome."*

Code: ● Koordinierung	Weight score: 0

C1 200224, 136 - 146

Created: 01.05.2024 16:18 by jmlut, Modified: 01.05.2024 16:18 by jmlut

Area: 729	1.66%

15.

"Nur das sind so Blitzlichter und die Umsetzung dessen, was uns eigentlich klar
ist, dass wir da was verbessern könnten, funktioniert nicht so wie ich mir das
persönlich vorstellen würde. Und egal mit wem du redest, ob das jetzt mit den
Kollegen der Narkoseambulanz ist, dann sieht das jeder, nur es funktioniert die
Umsetzung nicht sozusagen."

*“But these are just flashes of insight, and implementing what we actually know*

*could be improved doesn't work the way I personally would imagine it. And no matter who you talk to, whether it's colleagues in the anaesthesia clinic, everyone sees it, but the*

*implementation doesn't work, so to speak."*

Code: ● Koordinierung	Weight score: 0

C1 200224, 148 - 152

Created: 01.05.2024 16:18 by jmlut, Modified: 01.05.2024 16:18 by jmlut

Area: 346	0.79%

16.

"Und unter dem Strich jetzt nach Wochen des Durchgehens von diesen einzelnen
Operationen sind wir da gelandet, was eigentlich schon am Anfang klar war, wer
überprüft das Ganze und wer macht es. Und da haben wir gesagt, okay, wir hätten
gerne eine ERAS Nurse, also jemand, der das betreut und beobachtet.
Und dieser Prozess dauert jetzt schon eineinhalb Jahre. Und wo wir immer noch
darauf warten, ob wir mit diesem Projekt starten können, ob der Vorstand, das
Haus, bereit ist, bei Pflegekräften-Mangel etc., jemand extra abzustellen, der
eben, also das Erkennen der Anämie ist nicht das Problem, das sehe ich, das sind
die meisten von uns."

*"And the bottom line now, after weeks of going through these individual*

*operations, is that we have ended up where we knew we would be from the start: who*

*will review the whole thing and who will do it. And so we said, okay, we would*

*like an ERAS nurse, someone who will supervise and monitor the process.*

*And this process has been going on for a year and a half now. And we are still*

*waiting to see if we can start this project, if the board, the hospital, is prepared, given the shortage of nursing staff, etc., to assign someone extra to do this. Recognising anaemia is not the problem, I can see that, most of us can see that."*

Code: ● Koordinierung	Weight score: 0

C1 200224, 208 - 217

Created: 01.05.2024 16:29 by jmlut, Modified: 01.05.2024 16:29 by jmlut

Area: 664	1.51%

17.

"Und dann, was ich dann schon mache, ist ja, dass ich die zuweise zu einer
Ernährungsberatung, wo wie ich schon eingangs gesagt, das ist für die Katze, die
reden da auch mit damit, eben aber keiner schaut, ob er das macht und umsetzt zu
Hause."

*"And then what I do is refer them to a nutritionist, where, as I said at the beginning, it's for the cat, they talk to them about it, but no one checks to see if they're doing it and implementing it at home."*

Code: ● Koordinierung	Weight score: 0

C1 200224, 248 - 251

Created: 01.05.2024 16:32 by jmlut, Modified: 01.05.2024 16:32 by jmlut

Area: 245	0.56%

18.

"Und so beißt sich die Katze wieder in den Schwanz. Und da fehlt mir zum einen
eben die koordinative Stelle sozusagen. Das wäre mein persönlicher Wunsch"

*"And so we're back to square one. And that's where I feel the coordinating body is lacking, so to speak. That would be my personal wish."*

Code: ● Koordinierung	Weight score: 0

C1 200224, 273 - 274

Created: 01.05.2024 16:34 by jmlut, Modified: 01.05.2024 16:34 by jmlut

Area: 152	0.35%

19.

"Dass wir so eine Koordinationsstelle bekommen, dass das jemand ist, also das ist
jetzt eine Ordinationsassistentin oder eine Schwester, die speziell ausgebildet
ist, aber die gerade diese Patienten eben dann nachverfolgt und schaut, ob das
eh alles funktioniert."

*"That we get a coordination centre, that there is someone, that is now a doctor's assistant or a nurse who is specially trained but who then follows up on these patients and checks whether everything is working."*

Code: ● Koordinierung	Weight score: 0

C1 200224, 277 - 280

Created: 01.05.2024 16:34 by jmlut, Modified: 01.05.2024 16:34 by jmlut

Area: 265	0.60%

20.

"Das ist an sich, wer mir das wurscht, wo die angesiedelt ist. Hauptsache, ich
habe einen Ansprechpartner, wo ich sagen kann, ich habe jetzt da Patient XY,
bitte kümmern Sie sich um den sozusagen, weil dem steht eine sehr große
Operation bevor, hat im günstigsten Fall, also wenn es ein Krankheitsbild ist,
wo man präoperativ Chemo macht, dann hat man die Zeit, den wo möglich zu
optimieren, oder zumindest so zu betreuen, dass er nicht noch schlechter wird."

*"That's fine with me, I don't care where it's located. The main thing is that I*

*have a contact person to whom I can say, I now have patient XY, please take care of him, so to speak, because he is facing a very major operation ahead of him. In the best case scenario, i.e. if it is a condition where you do preoperative chemotherapy, then you have the time to optimise it where possible or at least to care for him in such a way that he does not get any worse."*

Code: ● Koordinierung	Weight score: 0

C1 200224, 285 - 290

Created: 01.05.2024 16:35 by jmlut, Modified: 01.05.2024 16:35 by jmlut

Area: 462	1.05%

21.

"Aber dann ist es so, dass das dann genau an den Dingen, die ich vorher schon
gesagt habe, meistens scheitert eben. Du nimmst dann Kontakt auf mit all jenen
Stellen, die da eigentlich dazugehören würden, und du kriegst dann immer die
gleichen Antworten.
Ja, wer soll das machen eben? Ich habe weder die Leute dafür. Wir sind jetzt
schon am Anschlag, wir können nicht mehr. Und diese Betreuung können wir hier im
stationären Bereich nicht anbieten. Und ambulant machen wir nicht so irgendwie."

*"But then it's the case that it usually fails precisely because of the things I mentioned earlier. You then contact all the agencies that would actually be involved, and you always get the*

*same answers.*

*Yes, who is supposed to do that? I don't have the people for it. We are already*

*at our limit, we can't do any more. And we can't offer this kind of care here in the*

*inpatient sector. And we don't do it on an outpatient basis either."*

Code: ● Koordinierung	Weight score: 0

C1 200224, 385 - 392

Created: 01.05.2024 16:38 by jmlut, Modified: 01.05.2024 16:38 by jmlut

Area: 513	1.17%

22.

"Also ich glaube, dass halt unser Problem ist und das wird wahrscheinlich auch so
bleiben, dass halt so lange man nicht extra dafür Stellen schafft, die diese
Koordination übernehmen sei das jetzt eine ERAS-Nurse oder Koordinationskraft,
die schaut, dass diese Zuweisungen, die man für die Prähabilitation braucht oder
für die Verbesserung des Patienten braucht, eben macht vielleicht war es früher
der Hausarzt, der jetzt auch mit anderen Dingen beschäftigt ist, der sich
vielleicht auch darum gekümmert hat."

*"So I think our problem is, and it will probably remain so, that as long as no special positions are created to take on this coordination, coordination, whether it's an ERAS nurse or a coordinator, who makes sure that the referrals needed for prehabilitation or*

*for the improvement of the patient are made. Perhaps it used to be the family doctor, who is now busy with other things, who might have taken care of this."*

Code: ● Koordinierung	Weight score: 0

C1 200224, 447 - 453

Created: 01.05.2024 21:29 by jmlut, Modified: 01.05.2024 21:29 by jmlut

Area: 514	1.17%

23.

"Und das sind dann halt nur- dann gibt es ein paar zaghafte Schritte, und das
funktioniert eine Zeit lang, und dann verläuft das wieder alles. Und ich meine,
jetzt haben wir schon vor, sag ich jetzt einmal so, also das Projekt läuft, also
wir wollen ERAS einführen auf der Chirurgie da, und nur wie du richtig sagst,
wenn man sich das in Österreich im Umfeld anschaut, dann gibt es ein
Nachbarspital eben, das hat das jetzt seit einem halben Jahr, Jahr - ERAS-Nurse,
aber das ist das erste ERAS-Zentrum in ganz Österreich, sonst gibt es da Nada,
und das ist eigentlich, sag ich jetzt einmal recht traurig, aber die - woran das
liegt, keine Ahnung, aber wahrscheinlich meistens eben daran, dass man halt
nichts hat eben, wie man das abrechnen kann."

*"And then there are just a few tentative steps, and that works for a while, and then everything fizzles out again. And I mean, now we already have plans, let's say, the project is underway, so we want to introduce ERAS in surgery there, and just as you rightly say,*

*if you look at the situation in Austria, there is only one neighbouring hospital that has had it for six months, a year – ERAS nurse, but that's the first ERAS centre in the whole of Austria, otherwise there's nothing, and that's actually, I'd say, quite sad, but the – why that is,*

*I have no idea, but probably mostly because there's just nothing to bill for it. "*

Code: ● Koordinierung	Weight score: 0

C1 200224, 555 - 564

Created: 01.05.2024 21:35 by jmlut, Modified: 01.05.2024 21:35 by jmlut

Area: 755	1.72%

24.

"dass wir da überhaupt kein Netzwerk haben in dem Sinn und da müsste es
irgendwie eine andere Koordinationsstelle geben, wo ich dann einen
Ansprechpartner hätte, wo ich weiß, okay, gut, das ist jetzt der Krebspatient
eben und bitte kümmert euch um den, denn der hat dann und dann seine OP und
dieses und jenes, sollte stattfinden.
Das hat vielleicht früher mit dem Hausarzt irgendwie funktioniert, aber auch da
wirst du eigentlich den Eindruck haben, dass der auch hilflos ist, weil er
eigentlich nicht die Möglichkeit hat, den irgendwo hinzuschicken und vermutlich
auch nur spekulativ, je weiter du am Land bist, desto schwieriger wird es dann."

*"We don't have any kind of network in that sense, so there would have to be*

*some kind of other coordination centre where I would have a contact person and I would know, okay, fine, this is the cancer patient and please take care of him, because he has his operation on such and such a date and this and that should take place.*

*That may have worked with the family doctor in the past, but even there you'll actually get the impression that he's helpless because he doesn't really have the option of sending him anywhere, and probably only speculatively, the further out in the country you are, the more difficult it becomes."*

Code: ● Koordinierung	Weight score: 0

C1 200224, 701 - 710

Created: 01.05.2024 21:42 by jmlut, Modified: 01.05.2024 21:42 by jmlut

Area: 669	1.52%

25.

"Und auch den organisatorischen Weg, den das Ganze benötigt, weil ich glaube, das ist so
die größte Hürde, dass man die gesamten Fachrichtungen, die dafür benötigt
werden, einfach alle an den Tisch bringt und dann ein Weg findet, wie das
möglichst effizient und durch den Alltag gebracht werden kann."

*"And also the organisational path that the whole thing requires, because I believe that*

*the biggest hurdle is simply bringing all the disciplines that are needed for this*

*to the table and then finding a way to implement it as efficiently as possible in everyday life."*

*Code: ● Koordinierung	Weight score: 0*

C2 180324, 109 - 113

Created: 20.05.2024 21:18 by Jan, Modified: 20.05.2024 21:18 by Jan

Area: 303	0.89%

26.

"Ich glaube, das Konzept als solches einfach den Weg in den klinischen Alltag
noch nicht so gefunden hat, weil es bedarf irgendwann einer Koordination. Wer
das übernimmt, glaube ich, ist egal. Aber es bedarf einer Koordination, weil es
einfach verschiedene Teilbreiche betrifft."

*"I believe that the concept as such has simply not yet found its way into everyday clinical practice because it requires coordination at some point. I don't think it matters who*

*takes on this task. But coordination is necessary because it simply affects different areas."*

Code: ● Koordinierung	Weight score: 0

C2 180324, 157 - 160

Created: 20.05.2024 21:18 by Jan, Modified: 20.05.2024 21:18 by Jan

Area: 280	0.82%

27.

"Also ich glaube, das Grundproblem bei diesem ganzen Konzept an sich ist die
Tatsache, dass es verschiedene Player braucht, die alle miteinander arbeiten
müssen."

*"So I think the fundamental problem with this whole concept is the fact that it requires different players who all have to work together."*

Code: ● Koordinierung	Weight score: 0

C2 180324, 261 - 263

Created: 20.05.2024 21:22 by Jan, Modified: 20.05.2024 21:22 by Jan

Area: 162	0.48%

28.

"Ich glaube, dass es wahrscheinlich, wenn man jetzt eben ein größeres Haus sich
anschaut, es kann vielleicht an räumlichen Dingen schwierig werden, wenn die
verschiedenen Fachrichtungen in unterschiedlichen Häusern oder in
unterschiedlichen Hausteilen untergebracht sind und damit die Wege für die
Patienten lang werden."

*"I think that if you look at a larger building now, it might be difficult in terms of space if the*

*different departments are housed in different buildings or different parts of the building, making the distances for patients long."*

Code: ● Koordinierung	Weight score: 0

C2 180324, 291 - 295

Created: 20.05.2024 21:24 by Jan, Modified: 20.05.2024 21:24 by Jan

Area: 323	0.95%

29.

"Es kann schwierig sein, wenn die verschiedenen Ambulanzzeiten sich entweder
überschneiden oder nicht überschneiden, dass es für den Patienten sehr aufwendig
wird und damit die Patientenmotivation leidet."

*"It can be difficult if the various outpatient clinic times either overlap or do not overlap, making it very time-consuming for the patient and thus reducing patient motivation."*

Code: ● Koordinierung	Weight score: 0

C2 180324, 297 - 299

Created: 20.05.2024 21:23 by Jan, Modified: 20.05.2024 21:23 by Jan

Area: 205	0.60%

30.

"Und ich glaube, dass man da, wenn man
so was aus der Taufe hebt, vielleicht in einem größeren Haus einfach gräumlich
und zeitlich möglicherweise, einfach weil es verschiedene Fachrichtungen
betrifft, an die Limits kommen kann."

*"And I think that if you start something like this at , you might reach the limits in terms of space and time in a larger hospital, simply because it involves different specialities ."*

Code: ● Koordinierung	Weight score: 0

C2 180324, 308 - 311

Created: 20.05.2024 21:24 by Jan, Modified: 20.05.2024 21:24 by Jan

Area: 229	0.67%

31.

"Ich glaube, es braucht jemanden, der das koordiniert mit viel Liebe
fürs Werk, vielleicht so auf die Art, aber dann auch viele Kollegen, die sich
davon anstecken lassen und dann damit machen und das entsprechend auch an den
Patienten übertragen können."

*"I think it needs someone who coordinates this with a lot of love for the work, perhaps in this way, but then also many colleagues who are infected by it and can then do it and transfer it accordingly to the patient."*

Code: ● Koordinierung	Weight score: 0

C2 180324, 370 - 373

Created: 20.05.2024 21:26 by Jan, Modified: 20.05.2024 21:26 by Jan

Area: 255	0.75%

32.

"Ich glaube, dass das Wesentliche ist, dass man sich personell
und strukturell überlegt, wie das effizient gestaltet werden kann, weil man dann,
 glaube ich, viele Patienten in das Programm bekommt, wenn man eine zeitlich und
räumlich effiziente Lösung basteln kann."

*"I believe that the essential thing is to think about how this can be designed efficiently in terms of personnel and structure, because then, I believe, you can get many patients into the programme if you can create an efficient solution in terms of time and space."*

Code: ● Koordinierung	Weight score: 0

C2 180324, 551 - 554

Created: 20.05.2024 21:41 by Jan, Modified: 20.05.2024 21:41 by Jan

Area: 267	0.78%

2.3. Interdisziplinäre Zusammenarbeit

1.

"also irgendjemand muss halt den Fall führen aus meiner Sicht und dem
Patienten das Portfolio dann aufspannen und mit ihm das durchgehen. Und dann
werden da wahrscheinlich, also wenn man sich den Versorgungsprozess dann
überlegt, ist es ja so, dass in den Krankenhäusern selbst natürlich auch immer
Gesundheitsberufe da sind, DiätologInnen und PhysiotherapeutInnen und so."

*"So someone has to manage the case from my point of view and then draw up the portfolio for the patient and go through it with them. And then there will probably be, well, if you think about the care process then, it is the case that in the hospitals themselves, of course, there are always health professions, dieticians and physiotherapists and so on."*

Code: ● Interdisziplinäre Zusammenarbeit	Weight score: 0

S1 080324, 217 - 221

Created: 20.05.2024 17:06 by Jan, Modified: 20.05.2024 17:06 by Jan

Area: 375	1.44%

2.

"dass das interdisziplinär
sein muss. Das heißt, du musst dich wieder mit den Internisten, mit den
Diätologinnen, mit den Psychologen, den Physiotherapeuten... dass das auch so
ein Konglomerat wird, dass sich interdisziplinär austauscht, das ist einfach
eher selten, finde ich.
Also, vielleicht noch Physio / Ergo oder halt mit den Ärzten, mit denen du oft
zu tun hast, aber sonst..."

*"That it has to be interdisciplinary. That means you have to work with internists, dieticians, psychologists, physiotherapists... It becomes such a conglomerate, with interdisciplinary exchanges, which I think is quite rare.*

*So, maybe physio/ergo or just with the doctors you often deal with, but otherwise..."*

Code: ● Interdisziplinäre Zusammenarbeit	Weight score: 0

PT1 140324, 271 - 278

Created: 20.05.2024 20:05 by Jan, Modified: 20.05.2024 20:05 by Jan

Area: 405	1.14%

3.

"Wo das dann alles zusammenläuft und wo du dann nachher alles beinander
hast, dann nützt es ja was. Die Situation ist total klasse, wenn ich weiß, ich
habe jetzt von dem Herrn So und So eine Zuweisung, der sagt mir dann, das ist
das Hauptproblem in seinen Augen, dann schauen wir das an und sagen, ja, ich
stimm dir zu, oder ich glaube, dass da vielleicht das sein kann."

*"When everything comes together and you have everything in one place, then it's useful. The situation is great when I know that I now have a referral from Mr So-and-so, who tells me that this is the main problem in his eyes, then we look at it and say, yes, I agree with you, or I think that might be the case."*

Code: ● Interdisziplinäre Zusammenarbeit	Weight score: 0

PT1 140324, 289 - 293

Created: 20.05.2024 20:06 by Jan, Modified: 20.05.2024 20:06 by Jan

Area: 373	1.05%

4.

"Darum der Wille und der Wunsch zusammenzuarbeiten in der Prähabilitation war
natürlich da, weil der Chirurg auch davon profitiert hätte, und der Anästhesist
vor allem, aber es ist wie immer ums Geld gegangen, dass das nicht bezahlt wurde."

*"So the will and desire to work together in prehabilitation was naturally there, because the surgeon would also have benefited from it, and the anaesthetist in particular, but as always, it was about money, that it wasn't paid for."*

Code: ● Interdisziplinäre Zusammenarbeit	Weight score: 0

K1 280224, 184 - 186

Created: 09.05.2024 20:28 by jmlut, Modified: 09.05.2024 20:28 by jmlut

Area: 240	0.65%

5.

"darum glaube ich, dass diese Vernetzung der
verschiedenen Medizin- oder der verschiedenen Gruppen, der verschiedenen
medizinischen Betreuungsgruppen und der verschiedenen Disziplinen einfach schon
vor Ort sein muss.
Natürlich sind die im Krankenhaus vor Ort alle, aber ob die auch mit
Rehabilitation und Prähabilitation, sich erstens dafür interessieren und
zweitens die Ressourcen haben, glaube ich nicht. Und da glaube ich, dass einfach
die Rehabilitationseinrichtungen dafür die idealen Voraussetzungen haben, weil
man dort auch eben diese einzelnen Dinge, die man eventuell ganz
patientenzentriert präferieren kann und muss, dass die einfach dort schon besser
vor Ort auch organisiert werden können."

*"That's why I believe that this networking between different medical or different groups, different medical care groups and different disciplines simply has to be in place locally.*

*Of course, they are all there in the hospital, but I don't think they are interested in rehabilitation and prehabilitation, firstly, and secondly, I don't think they have the resources. And I believe that rehabilitation facilities simply have the ideal conditions for this, because*

*there you can also organise these individual things, which you may prefer and need to be completely patient-centred, better on site."*

Code: ● Interdisziplinäre Zusammenarbeit	Weight score: 0

K1 280224, 317 - 328

Created: 09.05.2024 20:31 by jmlut, Modified: 09.05.2024 20:31 by jmlut

Area: 731	1.97%

6.

"Das heißt, die Slots in der Ambulanz dann freimachen, wo man diese Patienten
noch einmal zusätzlich sieht. Das ist in einem Krankenhaus, wo Platzmangel und
Terminenge schon sehr hoch ist, gar nicht so einfach.
Das heißt, das muss man dann halt zwischen den Leitern, den motivierten Leitern
und Mitarbeiten, also Leitern absprechen, die motivierte Mitarbeiter halt haben,
dass man diese Patienten überhaupt unterbringt."

*"That means freeing up slots in the outpatient clinic, where these patients*

*are seen again. That's not so easy in a hospital where space is limited and*

*appointments are already very tight.*

*That means you have to discuss it between the managers, the motivated managers*

*and employees, i.e. managers who have motivated employees, so that these patients can be accommodated at all."*

Code: ● Interdisziplinäre Zusammenarbeit	Weight score: 0

A1 210224, 172 - 178

Created: 09.05.2024 17:18 by jmlut, Modified: 09.05.2024 17:18 by jmlut

Area: 440	1.11%

7.

"Also auch das Platzproblem ist ein wesentliches. Das heißt, hier noch Platz mehr
zu finden für zusätzliche Leistungen ist oft ganz schwierig. Die Verwaltung ist
da im Prinzip dann sozusagen dahintergelegt, weil wenn sozusagen diese Teams,
die ich da angesprochen habe, sagen wir mal die Chirurgie und die Physiotherapie,
 die Diätologen, die sich finden, die auch sonst intensiv zusammenarbeiten."

*"So, the space problem is also a significant one. That means it is often very difficult to find more space here for additional services. The administration is then, in principle, behind it, so to speak, because when these teams, which I mentioned, let's say surgery and physiotherapy,*

*the dieticians, who are also working together intensively in other areas, come together."*

Code: ● Interdisziplinäre Zusammenarbeit	Weight score: 0

A1 210224, 262 - 266

Created: 09.05.2024 17:25 by jmlut, Modified: 09.05.2024 17:25 by jmlut

Area: 399	1.00%

8.

"In anderen Bereichen haben wir zum Beispiel die bariatrische Chirurgie
angesprochen, wo es ganz natürlich ist, dass man viel, sehr viel tut, auch mit
weiteren Berufsgruppen. Wenn die sich erst mal finden und da zustimmen, dann
läuft es.
*Dann sind hier die Wege ganz kurz und dann kann man das auch sehr schnell
umsetzen, wenn die einzelnen Teams sagen, das funktioniert gut."*

*"In other areas, we have addressed bariatric surgery, for example, where it is quite natural to do a lot, a great deal, even with other professional groups. Once they find each other and agree, then it works.*

*Then the paths are very short here and you can implement it very quickly if the individual teams say it works well."*

Code: ● Interdisziplinäre Zusammenarbeit	Weight score: 0

A1 210224, 268 - 274

Created: 09.05.2024 17:25 by jmlut, Modified: 09.05.2024 17:25 by jmlut

Area: 396	1.00%

9.

"Wir haben uns schon bemüht, das von der Anästhesieambulanz aus zu machen und
Menschen auch zu optimieren. Von chirurgischer Seite war es eher so, dass da die
Meinung vorgeherrscht hat, das sei Aufgabe der Anästhesie, wann wir uns das
schon wünschen würden.
Also interdisziplinäre Zusammenarbeit bis auf wenige Ausnahmen war da eher
sekundär. Nicht vorhanden."

*"We have already tried to do this from the anaesthesia outpatient clinic and*

*to optimise people as well. From the surgical side, the prevailing opinion was that*

*this was the anaesthesia department's job, whenever we wanted it to be.*

*So, with a few exceptions, interdisciplinary cooperation was rather secondary. Non-existent."*

Code: ● Interdisziplinäre Zusammenarbeit	Weight score: 0

A2 220422, 122 - 128

Created: 09.05.2024 19:37 by jmlut, Modified: 09.05.2024 19:37 by jmlut

Area: 380	1.07%

10.

"Also bei den Strukturen, in denen ich noch gearbeitet habe, hätte das ein
enormer Zeit- und Kraftaufwand bedurft, um da auch die nötige Motivation bei den
chirurgischen Partnern hervorzurufen."

*"In the structures I have worked in, it would have taken an enormous amount of time and effort to generate the necessary motivation among the surgical partners."*

Code: ● Interdisziplinäre Zusammenarbeit	Weight score: 0

A2 220422, 138 - 140

Created: 09.05.2024 19:38 by jmlut, Modified: 09.05.2024 19:38 by jmlut

Area: 194	0.54%

11.

"Vorstellen könnte ich es mir bei eher kleinen chirurgisch tätigen Abteilungen,
wie gesagt, im eigenen Krankenhaus kenne ich eine Abteilung, die sehr effizient
präoperativ arbeiten. Es hat sich auch auf einer größeren chirurgischen
Abteilung, hat sich die Sache schon Richtung positiven Denken entwickelt, dass
die sehr aufgeschlossen sind, vor allem was Patient-Blood-Management betrifft,
aber es erfordert, glaube ich, wirklich viel Energie und Zeit, also eigentlich
einen Kollegen, der sich für das sehr begeistert und interessiert, aber
freigestellt ist, um das Modell auf die Beine zu stellen."

*"I could imagine it working in smaller surgical departments. As I said, I know of a department in my own hospital that works very efficiently in the preoperative phase. In a larger surgical department, things have also developed in a positive direction, with people being very open-minded, especially when it comes to patient blood management, but I think it really requires a lot of energy and time, so basically a colleague who is very enthusiastic and interested in this, but who is free to get the model off the ground."*

Code: ● Interdisziplinäre Zusammenarbeit	Weight score: 0

A2 220422, 142 - 149

Created: 09.05.2024 19:38 by jmlut, Modified: 09.05.2024 19:38 by jmlut

Area: 604	1.69%

12.

"Wenn man alte Krankenhäuser sieht, dann sind die immer noch, und das sehen wir
auch hier, immer noch so aufgebaut, dass es ganz viele kleine Einheiten gibt, wo
jeder vor sich hinwerkelt. Und es ist extrem schwer, so ein Projekt aufzubauen,
wo ganz viele Leute aus verschiedensten Bereichen zusammenkommen."

*"When you look at old hospitals, they are still, and we see this here too, structured in such a way that there are lots of small units where everyone works away on their own. And it is extremely difficult to set up a project like this, where lots of people from different areas come together."*

Code: ● Interdisziplinäre Zusammenarbeit	Weight score: 0

A2 220422, 611 - 614

Created: 09.05.2024 19:53 by jmlut, Modified: 09.05.2024 19:53 by jmlut

Area: 308	0.86%

13.

"Ich habe leider das Gefühl, es ist schlechter geworden, also dass sich jeder
noch mehr zurückzieht auf seine Profession, auf seine Nische, in der er arbeitet
und das halt macht und dann den Ort des Geschehens so schnell wie möglich
verlässt."

*"Unfortunately, I have the feeling that it has gotten worse, that everyone is*

*withdrawing even more into their profession, into the niche in which they work*

*and do their job, and then leave the scene as quickly as possible ."*

Code: ● Interdisziplinäre Zusammenarbeit	Weight score: 0

A2 220422, 619 - 622

Created: 09.05.2024 19:53 by jmlut, Modified: 09.05.2024 19:53 by jmlut

Area: 244	0.68%

14.

"da sind dann immer so kurze Initiativen mit, ja wir machen bei jedem von diesen Patienten ein Diätkonsil, ein Ernährungskonsil. Wir machen auch Physiotherapiezuweisung, diese Dinge mache ich beim Ösophaguskarzinom, aber die verlaufen im Sand."

*"There are always such short initiatives with, yes, we do a diet consultation, a nutrition consultation, for each of these patients. We also do physiotherapy referrals, I do these things for oesophageal cancer, but they come to nothing."*

Code: ● Interdisziplinäre Zusammenarbeit	Weight score: 0

C1 200224, 127 - 132

Created: 01.05.2024 16:17 by jmlut, Modified: 01.05.2024 16:17 by jmlut

Area: 263	0.60%

15.

"Und wir haben dann so, dass wir dann eben,
wenn ich das gesehen habe, dass das nicht gut funktioniert und dann sprichst du
mit Diätberatern und sagst, okay, was habt ihr jetzt gemacht oder könnt ihr den
Patienten weiterbetreuen?
Ja, wir haben gemacht, gesagt, was er essen soll, wie er essen soll. Und das er
auf seine Kalorien kommen wird. Und das war es, das ist ein einmaliges Gespräch.
Und warum dann eine weitere Betreuung quasi nicht möglich ist, das scheitert
dann an - also das ist dann die Antwort, die ich dann bekomme - ja, wir sind für
den stationären Bereich zuständig und können den Patienten ja dann nicht die
ganze Zeit betreuen."

*"And then we have it so that when I see that it's not working well, you talk to dieticians and say, okay, what have you done now or can you continue to care for the patient?*

*Yes, we did, we told him what to eat and how to eat. And that he would get his calories. And that was it, that was a one-time conversation. And why further care is virtually impossible, that fails then because – well, that's the answer I get – yes, we are responsible for*

*the inpatient area and can't care for the patient all the time."*

Code: ● Interdisziplinäre Zusammenarbeit	Weight score: 0

C1 200224, 158 - 168

Created: 01.05.2024 16:19 by jmlut, Modified: 01.05.2024 16:19 by jmlut

Area: 671	1.53%

16.

"Du kannst uns eine Zuweisung machen. Und
was ich für viele Gespräche mit dem Chef der Physiotherapie im Haus persönlich...
 aber dann für diese eine Patientengruppe beim Ösophaguskarzinom haben wir es
dann zumindest durchgesetzt, dass ich, wenn ich eine fachärztliche Begutachung
mache - dass die die anschauen, was machen die, die zeigen denen Atemübungen,
und aus."

*"You can give us a referral. And what I personally discussed many times with the head of physiotherapy in the hospital... but then for this one group of patients with oesophageal cancer, we at least managed to get it through that when I do a specialist assessment*

*– that they look at what they're doing, they show them breathing exercises, and that's it."*

Code: ● Interdisziplinäre Zusammenarbeit	Weight score: 0

C1 200224, 178 - 183

Created: 01.05.2024 16:23 by jmlut, Modified: 01.05.2024 16:23 by jmlut

Area: 370	0.84%

17.

"Ich habe keine Ahnung, ob du dann zum Beispiel als Diätberaterin eben - ist zum
Beispiel das Feedback, was ich von denen bekomme, ist, ich kann einmal sprechen
mit denen und aus. Aber die kann die jetzt nicht, quasi, während der drei Monate
von der Diagnosestellung bis zur OP begleiten, sondern die reden einmal mit
denen, das war die Zuweisung und aus."

*"I have no idea whether you, as a dietitian, for example, would be able to give me feedback based on what I hear from them. I can talk to them once and that's it. But they can't accompany them during the three months from diagnosis to surgery, they just talk to them once and that's it."*

Code: ● Interdisziplinäre Zusammenarbeit	Weight score: 0

C1 200224, 571 - 575

Created: 01.05.2024 21:35 by jmlut, Modified: 01.05.2024 21:35 by jmlut

Area: 358	0.81%

18.

"Ich glaube, dass es hauptsächlich die Physiotherapeuten und die Diätologen
anbelangt, aber auch die Psychologen, denn wenn die das alle ernsthaft betreiben,
 sich wirklich dem einzelnen Patienten widmen und ich glaube, die
Prähabilitation hat extrem viel mit Motivation der Patienten zu tun. Weil wenn
die motiviert sind, dann werden sie mitmachen, wenn man sie nicht abholt und sie
nicht gescheit aufklärt."

*"I believe that it mainly concerns physiotherapists and dieticians, but also psychologists, because if they all take it seriously, really devote themselves to the individual patient, I believe that prehabilitation has a lot to do with patient motivation. Because if they are motivated, then they will participate, even if you don't pick them up and explain things to them properly."*

Code: ● Interdisziplinäre Zusammenarbeit	Weight score: 0

C2 180324, 179 - 184

Created: 20.05.2024 21:21 by Jan, Modified: 20.05.2024 21:21 by Jan

Area: 411	1.21%

19.

"Und dann, glaube ich, kann das was sein, was sehr gut wirkt, aber eben genau bei
besagten Berufsgruppen, glaube ich, braucht es viel Zeit."

*"And then, I think, it could be something that works very well, but precisely with*

*the professional groups in question, I think it will take a lot of time."*

Code: ● Interdisziplinäre Zusammenarbeit	Weight score: 0

C2 180324, 194 - 195

Created: 20.05.2024 21:21 by Jan, Modified: 20.05.2024 21:21 by Jan

Area: 139	0.41%

20.

"Je größer der Kreis wird, und bei uns betrifft es insbesondere die Pflege, desto
schwieriger ist es dann, die so mitzunehmen und denen zu erklären, warum ein
gewisser Mehraufwand notwendig ist um dieses Ziel zu erreichen, dass die
Komplikationsrate sinkt, wenn man es jetzt einmal ganz salopp formuliert."

*"The larger the circle becomes, and in our case this particularly affects nursing care, the*

*more difficult it is to get them on board and explain to them why a certain amount of extra effort is necessary to achieve this goal, that the complication rate will decrease, to put it bluntly."*

Code: ● Interdisziplinäre Zusammenarbeit	Weight score: 0

C2 180324, 266 - 269

Created: 20.05.2024 21:23 by Jan, Modified: 20.05.2024 21:23 by Jan

Area: 307	0.90%

21.

"Ich glaube, dass es wahrscheinlich, wenn man jetzt eben ein größeres Haus sich
anschaut, es kann vielleicht an räumlichen Dingen schwierig werden, wenn die
verschiedenen Fachrichtungen in unterschiedlichen Häusern oder in
unterschiedlichen Hausteilen untergebracht sind und damit die Wege für die
Patienten lang werden."

*"I believe that if you look at a larger building now, it may be difficult in terms of space if the*

*different departments are housed in different buildings or different parts of the building, making the distances for patients long."*

Code: ● Interdisziplinäre Zusammenarbeit	Weight score: 0

C2 180324, 291 - 295

Created: 20.05.2024 21:24 by Jan, Modified: 20.05.2024 21:24 by Jan

Area: 323	0.95%

2.4. Planbarkeit der Prähabilitation

1.

"Am ehesten scheint es zu laufen in manchen Kliniken, die einen orthopädischen
Schwerpunkt haben, weil man diese Patienten auch relativ gut im Vorfeld planen
kann. Erstens, und zweitens weil sie auch ein großes Fitnessdefizit haben, wo
man auch gut ansetzen kann.
Im Unterschied dazu ist es zum Beispiel in unserem Krankenhaus so, dass wir sehr
viele onkologische Patienten sehen, die natürlich nicht Monate vorher sozusagen
symptomatisch werden, die man dann Monate vorher prähabilitieren kann, sondern
da ist meistens dann eine Symptomatik aufgrund eines Malignoms und die Strecke
hin bis zur Operation ist dann in der Regel kurz, da sprechen wir von mehreren
Wochen."

*"It seems to work best in some clinics that specialise in orthopaedics because these patients can be planned for relatively well in advance. Firstly, and secondly, because they also have a significant fitness deficit, which can be addressed effectively.*

*In contrast, in our hospital, for example, we see a lot of oncology patients who, of course, do not become symptomatic months in advance, so to speak, and who can then be prehabilitated months in advance. Instead, there are usually symptoms due to a malignancy, and the time until surgery is then usually short, we are talking about several weeks."*

Code: ● Planbarkeit der Prähabilitation	Weight score: 0

A1 210224, 69 - 79

Created: 09.05.2024 17:11 by jmlut, Modified: 09.05.2024 17:11 by jmlut

Area: 694	1.75%

2.

"Beim Pankreaskarzinom und so weiter ist es ja so, dass das diagnostiziert wird,
dann hat er womöglich einen Ikterus, und da hast Du eh nicht die Zeit, dass du
irgendetwas optimierst."

*"With pancreatic cancer and so on, it's the case that once it's diagnosed, the patient may have jaundice, and then you don't have the time to optimise anything anyway."*

Code: ● Planbarkeit der Prähabilitation	Weight score: 0

C1 200224, 292 - 294

Created: 01.05.2024 16:35 by jmlut, Modified: 01.05.2024 16:35 by jmlut

Area: 184	0.42%

3.

"Beim Pankreas momentan bei sechs Wochen."

*‘For pancreatic cancer, currently six weeks.’*

Code: ● Planbarkeit der Prähabilitation	Weight score: 0

C1 200224, 302 - 302

Created: 01.05.2024 16:35 by jmlut, Modified: 01.05.2024 16:35 by jmlut

Area: 40	0.09%

4.

"ohne dass es da eine spezielle Art der Abrechnung gibt, ist es wahrscheinlich gar nicht so einfach umzusetzen, dass man sagt, okay gut, sie haben jetzt einen Operationstermin in fünf Wochen, wir brauchen innerhalb der nächsten 10 Tage einen Prähabilitationstermin für Sie,
das Sie da instruiert werden."

*"Without a specific billing method, it's probably not that easy to say, 'OK, fine, you have a surgery appointment in five weeks, we need a prehabilitation appointment for you within the next 10 days so that you can be instructed. "*

Code: ● Planbarkeit der Prähabilitation	Weight score: 0

C2 180324, 338 - 342

Created: 20.05.2024 21:25 by Jan, Modified: 20.05.2024 21:25 by Jan

Area: 306	0.90%

3. Struktur des Gesundheitswesens

3.1. Vorhandensein ambulante Strukturen

1.

"Das wäre wahrscheinlich schon was, was dann eher irgendwie im wohnortnahen
Setting sein sollte, zumal ja auch nicht alle Leute irgendwie in der
Nachbarschaft ein Krankenhaus stehen haben, um sich mit diesen Themen dazu
beschäftigen.
Ich denke, dass so was natürlich irgendwie im Rahmen von der Primärversorgung,
der in Österreich derzeit alles irgendwie aus meiner Sicht so ein bisschen rein
gebucht und verantwortet wird, aber wenn das wirklich gut funktionieren könnte
oder sollte, könnte ich mir schon vorstellen, dass das zum Beispiel was ist, was
auch die Pflege im Rahmen der Primärversorgung machen könnte."

*"That would probably be something that should be done in a local setting, especially since not everyone has a hospital in their neighbourhood where they can deal with these issues.*

*I think that something like this would naturally be part of primary care, which in Austria is currently somewhat booked up and responsible for everything, in my opinion, but if it could really work well, I think that something like this should naturally be part of primary care,*

*which in Austria is currently, in my view, somewhat overbooked and overburdened, but if it could or should work really well, I could imagine that it is something that could also be done by nurses within the framework of primary care."*

Code: ● Vorhandensein ambulante Strukturen	Weight score: 0

S1 080324, 228 - 237

Created: 20.05.2024 17:11 by Jan, Modified: 20.05.2024 17:11 by Jan

Area: 638	2.45%

2.

"Also ich denke immer, das könnte ja was sein, was wirklich die Pflege oder vielleicht auch irgendwie so wahnsinnig viele Berufsgruppen haben wir dann ja nicht, aber und Pflege haben wir glaube ich noch am ausreichendsten in der Fläche, dass sie das vielleicht übernimmt. Und dann ist es wahrscheinlich schon was, was in der Wohnortnähe passieren muss und nicht im Krankenhaus."

*‘Well, I always think that could be something, something that nursing care or perhaps other professions could do. We don't have that many professional groups, but I think we have enough nursing staff to take on this task. And then it's probably something that has to happen close to home and not in a hospital.’*

Code: ● Vorhandensein ambulante Strukturen	Weight score: 0

S1 080324, 255 - 259

Created: 20.05.2024 17:12 by Jan, Modified: 20.05.2024 17:12 by Jan

Area: 380	1.46%

3.

"Ärztekammer eventuell, oder halt Krankenhäuser an sich, wenn die das anbieten wollen als Institution. Das würde glaube ich funktionieren, und sonst halt auch niedergelassene Ärzte, wenn sie die Ressourcen oder halt die Kapazitäten haben, dass sie, das sie so ein Konglomerat anbieten können oder hat auch die Räumlichkeiten haben oder die Ressourcen am Markt, dann würde es schon gehen.
Aber wie gesagt, also das muss halt dann wirklich von Leuten ausgehen, die Räumlichkeiten und so haben. Sonst wird's schwierig."

*"Perhaps the medical association, or hospitals themselves, if they want to offer this as an institution. I think that would work, and otherwise also registered doctors, if they have the resources or the capacity to offer such a conglomerate, or if they have the premises or the resources on the market, then it would work.*

*But as I said, it really has to come from people who have the premises and so on. Otherwise, it will be difficult."*

Code: ● Vorhandensein ambulante Strukturen	Weight score: 0

PT1 140324, 427 - 435

Created: 20.05.2024 20:18 by Jan, Modified: 20.05.2024 20:18 by Jan

Area: 538	1.51%

4.

"Da würde ich immer glauben, dass es doch das gute Netz der ambulanten Rehabilitation gibt. Weil diese Patienten sind ja Großteils noch ambulant zu führen. Also ins Krankenhaus hinein würde ich die nicht nehmen wollen, sondern absolut in einem ambulanten Setting.
Und da ist ja der Bereich der ambulanten Rehabilitation jetzt österreichweit so gut ausgerollt, dass die sicher dahingehend, so wie die Stoffwechselrehabilitation, onkologische Rehabilitation, alle diese Bereiche ja jetzt auch abgedeckt werden, dass die präoperativ auch abgedeckt werden können."

*"I would always believe that there is a good network of outpatient rehabilitation. Because most of these patients can still be treated on an outpatient basis. So I wouldn't want to take them into hospital, but definitely into an outpatient setting.*

*And outpatient rehabilitation is now so well established throughout Austria that it certainly covers all these areas, such as metabolic rehabilitation and oncological rehabilitation, and can also cover preoperative care."*

Code: ● Vorhandensein ambulante Strukturen	Weight score: 0

K1 280224, 264 - 272

Created: 09.05.2024 20:29 by jmlut, Modified: 09.05.2024 20:29 by jmlut

Area: 582	1.57%

5.

"Ich habe gesehen, dass die Organisation und die Zeiteinteilung für den Patienten vor der Operation auch schon etwas sehr, sehr Schwieriges ist. Überhaupt wenn er psychisch jetzt so alteriert ist. Das heißt, man müsste ihm schon einen vorgegebenen Plan zur Verfügung stellen und dieser Plan - dass man das organisatorisch gut aufsetzt, da braucht man schon ein gewisses Organisationstalent und auch die passende EDV dazu und das ist etwas, was glaube
ich in den ambulanten Rehabilitationszentren, wo man ja gewohnt ist, verschiedene Berufsgruppen zu koordinieren, dass das dort einfach schon gut aufgestellt ist und dass dort auch schon stattfindet."

*"I have seen that the organisation and timing for the patient before the operation is also something very, very difficult. Especially when they are in such a state of mental distress. This means that you have to provide them with a set plan, and this plan – to set it up well in terms of organisation – requires a certain amount of organisational talent and the right IT, and that is something that I believe is already well established in outpatient rehabilitation centres, where they are used to coordinating different professional groups, and that it is already happening there."*

Code: ● Vorhandensein ambulante Strukturen	Weight score: 0

K1 280224, 286 - 294

Created: 09.05.2024 20:30 by jmlut, Modified: 09.05.2024 20:30 by jmlut

Area: 656	1.77%

6.

"Ich glaube nicht, dass man jetzt ein neues Tool schaffen müsste, sondern das ist, egal ob in stationären Rehabilitationszentren, da sehe ich wieder das Problem, dass die eh außerhalb der großen Ballungszentren liegen und dadurch für die Patienten, gerade so schwer kranke Patienten, wenn sie eine große Operation vor sich haben, nicht möglich ist, das umsetzen zu können, während wenn das eine ortsnahe Verbindung ist, wie ja die ambulanten Rehabilitationszentren in den großen Städten sind, ist da schon die Infrastruktur da, sind da schon die multidisziplinären Versorgungen da, und es ist ja so, dass es in der Prähabilitations, wie ich mir das vorstelle, ganz verschiedene Bedürfnisse gibt."

*"I don't think we need to create a new tool now, but rather, whether in inpatient rehabilitation centres, I see the problem again that they are located outside the major urban centres and therefore it is not possible for patients, especially those who are seriously ill and facing major surgery, to implement this. whereas if there is a local connection, as is the case with outpatient rehabilitation centres in large cities, the infrastructure is already in place, multidisciplinary care is already available, and it is the case that, as I see it, there are very different needs in prehabilitation."*

Code: ● Vorhandensein ambulante Strukturen	Weight score: 0

K1 280224, 296 - 304

Created: 09.05.2024 20:30 by jmlut, Modified: 09.05.2024 20:30 by jmlut

Area: 702	1.89%

7.

"darum glaube ich, dass diese Vernetzung der verschiedenen Medizin- oder der verschiedenen Gruppen, der verschiedenen medizinischen Betreuungsgruppen und der verschiedenen Disziplinen einfach schon vor Ort sein muss.
Natürlich sind die im Krankenhaus vor Ort alle, aber ob die auch mit Rehabilitation und Prähabilitation, sich erstens dafür interessieren und zweitens die Ressourcen haben, glaube ich nicht. Und da glaube ich, dass einfach die Rehabilitationseinrichtungen dafür die idealen Voraussetzungen haben, weil man dort auch eben diese einzelnen Dinge, die man eventuell ganz patientenzentriert präferieren kann und muss, dass die einfach dort schon besser vor Ort auch organisiert werden können."

*"That's why I believe that this networking between the various medical or different groups, the various medical care groups and the various disciplines simply has to be in place locally.*

*Of course, they are all there in the hospital, but I don't think they are interested in rehabilitation and prehabilitation, nor do they have the resources for it. And I believe that rehabilitation facilities simply have the ideal conditions for this, because there you can organise these individual things, which you may prefer and need to do in a patient-centred way, better on site."*

Code: ● Vorhandensein ambulante Strukturen	Weight score: 0

K1 280224, 317 - 328

Created: 09.05.2024 20:31 by jmlut, Modified: 09.05.2024 20:31 by jmlut

Area: 731	1.97%

8.

"Was nicht funktioniert hat, kann ich Ihnen vielleicht auch noch aus meiner Erfahrung sagen: die ambulante neurologische Rehabilitation wurde am Anfang so organisiert, dass eben die Patienten - das es zwar von der Versicherung bezahlt wurde, aber sie mussten zu einem extra Psychologen, sie mussten ihre Termine bei der Ergotherapeutin extra ausmachen, sie mussten den Termin beim Physiotherapeutin extra ausmachen, sie mussten dann wieder beim Orthopägen oder beim Neurologen den Termin extra ausmachen, auch im ambulanten Setting, da verliert.sich das ganze, Sondern wenn, dann sollte das wirklich zentriert, die Organisation in einer Hand sein und die Leute an eine Stelle kommen, also entweder, sei es ins Krankenhaus, sei es in ein ambulantes Rehaszentrum, sei es in ein Versorgungszentrum, sei es auch in Internisten oder Ordinationsgemeinschaften, die das auch anbieten wollen, wie auch immer."

*"I can also tell you from my own experience what didn't work: At the beginning, outpatient neurological rehabilitation was organised in such a way that patients – although it was paid for by insurance – had to see a separate psychologist, make separate appointments with the occupational therapist, make separate appointments with the physiotherapist, and then make separate appointments with the orthopaedist or neurologist, also in an outpatient setting. The whole thing should be centralised, with the organisation in one place and people coming to one location, whether it be a hospital, an outpatient rehabilitation centre, a care centre, or even internists or group practices that want to offer this service, whatever the case may be."*

Code: ● Vorhandensein ambulante Strukturen	Weight score: 0

K1 280224, 608 - 620

Created: 09.05.2024 20:43 by jmlut, Modified: 09.05.2024 20:43 by jmlut

Area: 911	2.45%

9.

"das war für mich sehr bezeichnend, dass wir eigentlich eines der wenigen Länder sind, wo es Kuren gibt und wo es diese Reha -Zentren gibt und andere Länder, die sich da an Kopf greifen eben, wie kann man jemanden drei Wochen lang da irgendwo stationär hinschicken und davon ausgehen, dass er nach drei Wochen dann als neuer Mensch wiederkommt und andere machen das ambulant eben, auch die Reha,"

*‘It was very telling to me that we are actually one of the few countries where there are health resorts and rehabilitation centres, while other countries are scratching their heads, wondering how you can send someone away for three weeks of inpatient treatment and expect them to come back a changed person after three weeks. Others do it on an outpatient basis, including rehabilitation.’*

Code: ● Vorhandensein ambulante Strukturen	Weight score: 0

C1 200224, 673 - 678

Created: 01.05.2024 21:40 by jmlut, Modified: 01.05.2024 21:40 by jmlut

Area: 399	0.91%

10.

"wahrscheinlich würden sie sich's vorstellen, damit ich gut in die OP reingehe, gehe ich drei Wochen vorher in ein Krankenhaus, damit ich dann im Krankenhaus fit bin, so ungefähr, also man geht quasi auf eine Reha, sonst noch auf Preha und da werde ich dann vorbereitet eben, weil es eben auch nach der Operation nicht die ambulanten Strukturen gibt und diese Behandlungen gibt, damit ich eben ambulant nach meiner Knieoperation oder nach meiner onkologischen Operation dort betreut werde im niedergelassenen Bereich, weil die werden dann alle eben irgendwo hingeschickt auf Reha sozusagen, wenn wir diese Strukturen nicht haben."

*"They would probably imagine that, in order for me to go into surgery in good shape, I would go to a hospital three weeks beforehand so that I would be fit when I got there, something like that. So you basically go to rehab, or maybe to a pre-rehabilitation facility, where I would be prepared, because after the operation there are no outpatient facilities or treatments available, so that I can be cared for on an outpatient basis after my knee operation or my oncological operation in a private practice, because otherwise everyone would be sent somewhere for rehabilitation, so to speak, if we didn't have these facilities."*

Code: ● Vorhandensein ambulante Strukturen	Weight score: 0

C1 200224, 686 - 694

Created: 01.05.2024 21:41 by jmlut, Modified: 01.05.2024 21:41 by jmlut

Area: 636	1.45%

11.

"Hier unter uns gibt es ein Zentrum für ambulante Reha, aber ich glaube, das funktioniert, ich weiß nicht wie gut, aber das ist die Ausnahme, also gibt es ambulante Reha, also wie gut die funktioniert, habe ich keine Ahnung, aber das ist eine große Ausnahme, wenn man das große Ganze betrachtet und ich glaube, so wie es nach der OP ist, so schlecht ist es auch vor der OP, dass dieses Außenrum, dass wir da überhaupt kein Netzwerk haben in dem Sinn und da müsste es irgendwie eine andere Koordinationsstelle geben, wo ich dann einen Ansprechpartner hätte, wo ich weiß, okay, gut, das ist jetzt der Krebspatient eben und bitte kümmert euch um den, denn der hat dann und dann seine OP und dieses und jenes, sollte stattfinden."

*"There is an outpatient rehabilitation centre here, but I think it works, I don't know how well, but that's the exception, so there is outpatient rehabilitation, but I have no idea how well it works, but that's a big exception when you look at the big picture, and I think that the situation after the operation it's just as bad as it was before the operation, that we have no network at all in that sense, and there should be some other coordination centre where I would have a contact person, where I would know, okay, well, this is the cancer patient, and please take care of him, because he has his operation on such and such a date, and this and that should take place."*

Code: ● Vorhandensein ambulante Strukturen	Weight score: 0

C1 200224, 696 - 705

Created: 01.05.2024 21:42 by jmlut, Modified: 01.05.2024 21:42 by jmlut

Area: 733	1.67%

3.2. Schnittstelle Intra- / Extramural

1.

"aus der Sicht des Kostenträgers dürfte es wahrscheinlich ein ähnlich gelagertes Problem sein, nämlich das Krankenhaus sagt, diese Sachen, die alle vor der Operation stehen, gerade bei elektiven Eingriffen gehen uns ja nichts an, sondern das ist ein Thema des niedergelassenen Bereichs und der niedergelassene Bereich sagt, na ja, das steht ja unmittelbar mit der bevorstehenden Operation, die im Bereich des Spitals stattfindet im Zusammenhang, deswegen geht es uns nichts an."

*" From the perspective of the cost bearer, it is likely to be a similar problem, namely that the hospital says that these matters, which all arise before the operation, especially in the case of elective procedures, are not our concern, but rather a matter for private practitioners, and the private practitioners say that, well, it is directly related to the upcoming operation, which takes place in the hospital, so it is not our concern. "*

Code: ● Schnittstelle Intra- / Extramural	Weight score: 0

S1 080324, 63 - 70

Created: 20.05.2024 16:57 by Jan, Modified: 20.05.2024 16:57 by Jan

Area: 483	1.85%

2.

"Das ist überall dort, wo halt die Brüche zwischen den Kostenträgern sind, ja in diesem Schnittstellenbereich zwischen intra- und extramuraler Versorgung. Da wird es schwierig, weil die Logik ist ja die, wenn die Prähabilitation im niedergelassenen Bereich stattfindet, spart sich der Krankenanstaltenträger was. Aber die Sozialversicherung kostet es was. Und da ist halt das Anreizsystem nicht so vorhanden, um solche Dinge aufzunehmen
in einen Leistungskatalog."

*"This is wherever there are gaps between cost bearers, i.e. in this interface area between inpatient and outpatient care. This is where it gets difficult, because the logic is that if prehabilitation takes place in private practice, the hospital operator saves money. But it costs the social security system money. And there isn't really an incentive system in place to include such things in a catalogue of services."*

Code: ● Schnittstelle Intra- / Extramural	Weight score: 0

S1 080324, 298 - 305

Created: 20.05.2024 17:14 by Jan, Modified: 20.05.2024 17:14 by Jan

Area: 486	1.87%

3.

"Ärztekammer eventuell, oder halt Krankenhäuser an sich, wenn die das anbieten wollen als Institution. Das würde glaube ich funktionieren, und sonst halt auch niedergelassene Ärzte, wenn sie die Ressourcen oder halt die Kapazitäten haben, dass sie, das sie so ein Konglomerat anbieten können oder hat auch die Räumlichkeiten haben oder die Ressourcen am Markt, dann würde es schon gehen.
Aber wie gesagt, also das muss halt dann wirklich von Leuten ausgehen, die Räumlichkeiten und so haben. Sonst wird's schwierig."

*"Perhaps the medical association, or hospitals themselves, if they want to offer this as an institution. I think that would work, and otherwise also registered doctors, if they have the resources or the capacity to offer such a conglomerate, or if they have the premises or the resources on the market, then it would work.*

*But as I said, it really has to come from people who have the premises and so on. Otherwise, it will be difficult."*

Code: ● Schnittstelle Intra- / Extramural	Weight score: 0

PT1 140324, 427 - 435

Created: 20.05.2024 20:18 by Jan, Modified: 20.05.2024 20:18 by Jan

Area: 538	1.51%

4.

"Ich fände es gut im Krankenhaus, weil genau da müssen sie her. Also spontan aus dem Bauch heraus, würde ich sagen, das, was mir Angst macht, da muss ich hin."

*‘I think it would be good in the hospital, because that's exactly where they need to be. So, spontaneously, from the gut, I would say that I have to go where I'm afraid to go.’*

Code: ● Schnittstelle Intra- / Extramural	Weight score: 0

P1 010324, 360 - 363

Created: 20.05.2024 18:53 by Jan, Modified: 20.05.2024 18:53 by Jan

Area: 177	0.46%

5.

"Denke ich mal, die Hausärzte müssen den Patienten sagen, sie haben eine OP, wissen sie, dass es da Prähabilitation gibt? So wie Geburtsvorbereitung, den machen sie den Kurs, das tut ihnen sicher gut, werden sie sehen."

*‘I think GPs need to tell patients who are about to have surgery that prehabilitation is available. It's like antenatal classes – they take the course and it's definitely good for them, as they will see.’*

Code: ● Schnittstelle Intra- / Extramural	Weight score: 0

P1 010324, 438 - 440

Created: 20.05.2024 18:55 by Jan, Modified: 20.05.2024 18:55 by Jan

Area: 219	0.57%

6.

"Darum der Wille und der Wunsch zusammenzuarbeiten in der Prähabilitation war natürlich da, weil der Chirurg auch davon profitiert hätte, und der Anästhesist vor allem, aber es ist wie immer ums Geld gegangen, dass das nicht bezahlt wurde."

*‘So there was naturally a willingness and desire to work together in prehabilitation, because the surgeon would also have benefited from it, and the anaesthetist in particular, but as always, it came down to money, because it wasn't paid for.’*

Code: ● Schnittstelle Intra- / Extramural	Weight score: 0

K1 280224, 184 - 186

Created: 09.05.2024 20:28 by jmlut, Modified: 09.05.2024 20:28 by jmlut

Area: 240	0.65%

7.

"darum glaube ich, dass diese Vernetzung der verschiedenen Medizin- oder der verschiedenen Gruppen, der verschiedenen medizinischen Betreuungsgruppen und der verschiedenen Disziplinen einfach schon vor Ort sein muss. Natürlich sind die im Krankenhaus vor Ort alle, aber ob die auch mit Rehabilitation und Prähabilitation, sich erstens dafür interessieren und zweitens die Ressourcen haben, glaube ich nicht. Und da glaube ich, dass einfach die Rehabilitationseinrichtungen dafür die idealen Voraussetzungen haben, weil man dort auch eben diese einzelnen Dinge, die man eventuell ganz patientenzentriert präferieren kann und muss, dass die einfach dort schon besser vor Ort auch organisiert werden können."

*"That's why I believe that this networking between the various medical or different groups, the various medical care groups and the various disciplines simply has to be in place locally. Of course, they are all there in the hospital, but I don't believe that they are interested in rehabilitation and prehabilitation, nor do they have the resources for it. And I believe that rehabilitation facilities simply have the ideal conditions for this, because there you can organise these individual things, which you may prefer and need to do in a patient-centred way, better on site."*

Code: ● Schnittstelle Intra- / Extramural	Weight score: 0

K1 280224, 317 - 328

Created: 09.05.2024 20:31 by jmlut, Modified: 09.05.2024 20:31 by jmlut

Area: 731	1.97%

8.

"Ich glaube auch, dass das Problem dieser Schnittstellen zwischen extramuralem / intramuralem Bereich ein Problem ist, dass man da zu wenig ökonomisch auch denkt und die Untersuchungen zu häufig wiederholt und dadurch sehr viele sowohl zeitliche Lehrläufe, also auch finanzielle Lehrläufe und zusätzliche Kosten entstehen, die nicht notwendig wären."

*‘I also believe that the problem of these interfaces between the extramural and intramural areas is that there is too little economic thinking involved and that examinations are repeated too often, resulting in a great deal of unnecessary time, financial resources and additional costs.’*

Code: ● Schnittstelle Intra- / Extramural	Weight score: 0

K1 280224, 399 - 403

Created: 09.05.2024 20:38 by jmlut, Modified: 09.05.2024 20:38 by jmlut

Area: 352	0.95%

9.

"Und jetzt müssen aus meiner Sicht, wie gesagt, noch die Strukturen geschaffen werden, um die Prähabilitation halt auch möglich zu machen."

*‘And now, in my opinion, as I said, the structures still need to be created to make prehabilitation possible.’*

Code: ● Schnittstelle Intra- / Extramural	Weight score: 0

A1 210224, 156 - 157

Created: 09.05.2024 17:16 by jmlut, Modified: 09.05.2024 17:16 by jmlut

Area: 138	0.35%

10.

"Wir haben auch einen sehr guten Zugriff zu diesen Mitarbeitern, die bei uns im Haus sind. Das heißt, die Standardisierung ist absolut gegeben. Wenn das Ganze dann passt, also wenn wir dann die Expertise haben, dann spricht auch nichts dagegen, dass man das nach außen mit gibt und mit ihnen kooperiert."

*‘We also have excellent access to these employees who work in-house. This means that standardisation is absolutely guaranteed. If everything fits together, i.e. if we have the expertise, then there is no reason not to share this externally and cooperate with them.’*

Code: ● Schnittstelle Intra- / Extramural	Weight score: 0

A1 210224, 377 - 380

Created: 09.05.2024 17:31 by jmlut, Modified: 09.05.2024 17:31 by jmlut

Area: 305	0.77%

11.

"Unsere Erfahrungen mit den Niedergelassenen sind grundsätzlich sehr gut. Das funktioniert grundsätzlich wirklich sehr gut. So weit sind wir aber noch nicht gekommen. Es ist schon aber so, dass aus derzeitiger Sicht der Aufwand, der finanzielle Aufwand pro Patient, wenn er jetzt praktisch prähospital ist, wenn jetzt prähospital ist, deutlich geringer ist."

*‘Our experience with registered doctors has been very positive overall. It generally functions very well. However, we have not yet reached that stage. It is true that, from the current perspective, the financial expenditure per patient is significantly lower when they are in the pre-hospital phase.’*

Code: ● Schnittstelle Intra- / Extramural	Weight score: 0

A1 210224, 382 - 386

Created: 09.05.2024 17:31 by jmlut, Modified: 09.05.2024 17:31 by jmlut

Area: 360	0.91%

12.

"Dann hat es einmal große Bestrebungen gegeben, dies nach außen zu verlagern in den niedergelassenen Bereich, wo es halt wieder schwierig ist: Patient kommt, man stellt irgendeinen Mangel fest, man stellt fest, es gehört optimiert, muss ihn nach draußen schicken, muss ihn zu den verschiedenen Fachrichtungen verweisen, wo es auch in Österreich draußen sehr schwierig ist, dann muss er eigentlich mit diesen neuen gesammelten Befunden nochmal wieder vorstellig werden.
Wenn man es anders macht und diese Untersuchungen nach außen ressourct und sagt, wiederkommen müssen sie nicht, aber wir müssen die Befunde sichten, ist ja trotzdem ein Mehraufwand. Im Prinzip macht man die Arbeit doppelt."

*"Then there was a major effort to outsource this to private practices, where it is again difficult: the patient comes in, you identify a deficiency, you determine that it needs to be optimised, you have to send them outside, you have to refer them to various specialists, which is also very difficult in Austria, and then they actually have to come back again with these new findings.*

*If you do it differently and outsource these examinations and say that they don't have to come back, but we have to review the findings, it's still extra work. In principle, you're doing the work twice."*

Code: ● Schnittstelle Intra- / Extramural	Weight score: 0

A2 220422, 110 - 120

Created: 09.05.2024 19:37 by jmlut, Modified: 09.05.2024 19:37 by jmlut

Area: 716	2.01%

13.

"Ich glaube, dass man den sehr gut einbinden könnte. Wahrscheinlich ist auch da ein digitales Verfahren von Vorteil. Aber da bedarf es einiger Hürden, die man noch aus dem Weg haben muss. Man muss natürlich den niedergelassenen Bereich ausweiten, damit die Kollegen das draußen überhaupt stemmen können. Dann muss man einen Katalog erstellen, was für sogenannte große oder mittelgroße Operationen überhaupt einmal wichtig ist und von Vorteil vielleicht für beide Parteien, chirurgisch oder eben Anästhesist oder Physiotherapeut.
Und man muss dafür auch finanzielle Mittel zur Verfügung stellen, weil es kann ja nicht sein, dass man das in den niedergelassenen Bereich auslagert, aber die Kollegen und Kolleginnen dafür auch nicht entlohnt werden."

*"I believe that it could be integrated very well. A digital process would probably also be advantageous here. But there are a few hurdles that need to be overcome first. Of course, the private practice sector needs to be expanded so that colleagues out there can actually manage it. Then you have to create a catalogue of what is important for so-called large or medium-sized operations and what might be advantageous for both parties, surgeons or anaesthetists or physiotherapists.*

*And you also have to provide financial resources for this, because you can't outsource it to private practices without remunerating colleagues for it."*

Code: ● Schnittstelle Intra- / Extramural	Weight score: 0

A2 220422, 341 - 352

Created: 09.05.2024 19:46 by jmlut, Modified: 09.05.2024 19:46 by jmlut

Area: 788	2.21%

14.

"Dann redet man mit den Physiotherapeuten: sagt einmal, könnt ihr nicht irgendwas machen, damit das besser geht?
Nein, also ambulant machen wir gar nichts. Wir sind nicht zuständig, also ich bin hier angestellt, also wir sind hier zuständig für den stationären Bereich"

*"Then you talk to the physiotherapists: say, can't you do something to make it better?*

*No, we don't do anything on an outpatient basis. We're not responsible for that. I'm employed here, so we're responsible for the inpatient area."*

Code: ● Schnittstelle Intra- / Extramural	Weight score: 0

C1 200224, 172 - 178

Created: 01.05.2024 16:22 by jmlut, Modified: 01.05.2024 16:22 by jmlut

Area: 304	0.69%

15.

"Extramural ist gar nichts, also zumindest nicht das ich wüsste. Also ich wüsste ganz ehrlich nicht, wenn der Patient vor mir sitzt, und ich sage - vielleicht ist das auch Ignoranz von mir, dass ich das dem Hausarzt das nicht zutraue, aber es ist so, dass ich halt als Spitalsarzt die Strukturen hier drinnen kenne. Ich hab aber keine Ahnung - ob ich dem Patienten jetzt dabei nett sage jetzt gehen Sie zum Hausarzt, und der soll sich jetzt bitte um das alles kümmern,
Ernährungsstatus, Physiotherapie und Dingsbums."

*"There's nothing extramural, at least not that I know of. To be honest, I wouldn't know if the patient were sitting in front of me and I said – maybe it's ignorance on my part that I don't trust the general practitioner with this, but the fact is that as a hospital doctor, I know the structures here. But I have no idea – whether I should now kindly tell the patient to go to their GP and ask them to take care of everything, nutritional status, physiotherapy and whatnot."*

Code: ● Schnittstelle Intra- / Extramural	Weight score: 0

C1 200224, 255 - 262

Created: 01.05.2024 16:32 by jmlut, Modified: 01.05.2024 16:32 by jmlut

Area: 537	1.22%

16.

"Und ich hab auch keine Ahnung, muss ich ganz ehrlich sagen, ob der das überhaupt umsetzen könnte, ob es da überhaupt von der Vergütung gibt oder was auch immer.

Also das muss ich gestehen, muss ich passen sozusagen."

*"And I have no idea, to be honest, whether he could even do it, whether there's any remuneration for it or whatever.*

*So I have to admit, I have to pass, so to speak."*

Code: ● Schnittstelle Intra- / Extramural	Weight score: 0

C1 200224, 264 - 266

Created: 01.05.2024 16:33 by jmlut, Modified: 01.05.2024 16:33 by jmlut

Area: 217	0.49%

17.

"Also ich kenne mich aus im stationären Bereich. Das heißt, ich kann, wenn ich der Meinung bin, dass der Physio und also präoperative Optimierung braucht, dann kann ich nur hier drinnen zuweisen. Und die hier drinnern sagen mir ich hab eigentlich auch nicht die Ressourcen für das."

*‘Well, I know my way around the inpatient sector. That means that if I think that the patient needs physiotherapy and preoperative optimisation, I can only refer them here. And the people here tell me that they don't really have the resources for that either.’*

Code: ● Schnittstelle Intra- / Extramural	Weight score: 0

C1 200224, 268 - 271

Created: 01.05.2024 16:33 by jmlut, Modified: 01.05.2024 16:33 by jmlut

Area: 283	0.64%

18.

"Aber dann ist es so, dass das dann genau an den Dingen, die ich vorher schon gesagt habe, meistens scheitert eben. Du nimmst dann Kontakt auf mit all jenen Stellen, die da eigentlich dazugehören würden, und du kriegst dann immer die gleichen Antworten.
Ja, wer soll das machen eben? Ich habe weder die Leute dafür. Wir sind jetzt schon am Anschlag, wir können nicht mehr. Und diese Betreuung können wir hier im stationären Bereich nicht anbieten. Und ambulant machen wir nicht so irgendwie."

*"But then it's usually the things I mentioned earlier that cause it to fail. You contact all the relevant authorities, and you always get the same answers.*

*Yes, who is supposed to do that? I don't have the people for it. We're already at our limit, we can't do any more. And we can't offer this kind of care here in the inpatient sector. And we don't do it on an outpatient basis either."*

Code: ● Schnittstelle Intra- / Extramural	Weight score: 0

C1 200224, 385 - 392

Created: 01.05.2024 16:38 by jmlut, Modified: 01.05.2024 16:38 by jmlut

Area: 513	1.17%

19.

"Und das ist halt das Problem eben, dass da diese, denke ich mir jetzt so, wenn ich darüber nachdenke, dass wir es immer noch nicht schaffen, diese Schnittstelle in den extramuralen Bereich zu verbessern. Weil der für uns, weil wir so in unseren Köpfen in diesem Krankenhaus festsitzen, glaube ich, und dass wir halt einfach gar nicht daran denken, eben da den
niedergelassenen Bereich (unter Anführungszeichen) miteinzubeziehen.
Und ich weiß es auch ehrlich gesagt gar nicht. Ich wüsste jetzt gar nicht -außer dass ich dem Patienten sage gehen sie dort hin, aber ob das dann..."

*"And that's precisely the problem, that, as I think about it now, we still haven't managed to improve this interface with the extramural sector. Because for us, because we're so stuck in our minds in this hospital, I think, and we just don't think about involving the*

*private practice sector (in quotation marks).*

*And to be honest, I don't really know. I wouldn't know – except to tell the patient to go there, but whether that would then..."*

Code: ● Schnittstelle Intra- / Extramural	Weight score: 0

C1 200224, 394 - 403

Created: 01.05.2024 16:39 by jmlut, Modified: 01.05.2024 16:39 by jmlut

Area: 620	1.41%

20.

"Genau, das ist das Problem, dass ich dann auch nicht weiß, wenn ich den dann hinschicke und sage, machen sie Physiotherapie, machen sie dieses oder jenes eben, sie brauchen Verbesserung, ob das dann auch so passiert, wie ich mir das vorstellen würde und so."

*‘Exactly, that's the problem, that I don't know if I send them there and say, do physiotherapy, do this or that, they need to improve, whether that will happen the way I imagine it would and so on.’*

Code: ● Schnittstelle Intra- / Extramural	Weight score: 0

C1 200224, 408 - 411

Created: 01.05.2024 16:39 by jmlut, Modified: 01.05.2024 16:39 by jmlut

Area: 260	0.59%

21.

"Dann habe ich das aufgeschrieben, also im Brief hineingeschrieben, jetzt ist das Chefarzt bewilligt, hat mir dann nach Monaten einer gesagt sozusagen, dass er das nicht bekommt, hat mich aber kein einziger Hausarzt jemals angerufen. Das heißt, die Frage lesen die den Brief überhaupt, machen die da irgendwas, interessiert es irgendjemandem, was wir da eigentlich gerne hätten und das ist das, was man so ein bisschen abgeht"

*‘Then I wrote it down, I mean, I put it in the letter, and now the chief physician has approved it, but after months, someone told me that he wouldn't get it, but not a single GP ever called me. That means, do they even read the letter, do they do anything about it, does anyone care what we actually want, and that's what's a bit frustrating.’*

Code: ● Schnittstelle Intra- / Extramural	Weight score: 0

C1 200224, 483 - 489

Created: 01.05.2024 21:31 by jmlut, Modified: 01.05.2024 21:32 by jmlut

Area: 446	1.01%

22.

"das hat glaube ich schon ein bisschen was damit zu tun eben, dass so in den Köpfen, wenn das Spital das will, das soll der Spital das auch bezahlen, sozusagen und sich darum kümmern und diese Vernetzung glaube ich ist in England wahrscheinlich, vermutlich besser, weil das
keine getrennten Töpfe sind, sondern wenn es halt das staatliche Gesundheitssystem ist, wo heute der niedergelassene Bereich und der Spitalsbereich ein Dach hat und bei uns geht das Match immer so hin und her, wer übernimmt das jetzt und wenn ich jetzt sage ich hätte gern Physiotherapie eben für den Patienten, dass der fit wird, dann sagt der Spital ja immer in dem Moment, wo er nicht im Spital ist, muss das der Niedergelassene bei uns machen und der Niedergelassene, vermute ich jetzt, das ist vielleicht eben, das warum es nicht so gut funktioniert, sagt dann halt, na ja, ich meine, ich übernehme jetzt nicht die Kosten für eine Physiotherapie, damit der dann im Spital fitter ist, also da soll doch das Spital schauen, dass die Strukturen passen, das ist jetzt so salopp, vielleicht eine Erklärungen dafür, aber diese Schnittstelle ist einfach furchtbar mühsam."

*"I think that has something to do with the fact that people think that if the hospital wants it, then the hospital should pay for it, so to speak, and take care of it, and I think this kind of networking is probably better in England because there are no separate funds, but rather it's the state health system, where today the private practice sector and the hospital sector are under one roof, whereas here the match always goes back and forth, who is responsible for it, and if I say I would like physiotherapy for the patient so that they can get fit, then the hospital always says that if they are not in hospital, the private practitioner has to do it, and the private practitioner, I suppose, that's perhaps why it doesn't work so well, then says, well, I mean, I'm not going to cover the costs of physiotherapy so that he can get fitter in hospital, so the hospital should make sure that the structures are right. That's a bit blunt, perhaps, but it explains why this interface is so terribly tedious."*

Code: ● Schnittstelle Intra- / Extramural	Weight score: 0

C1 200224, 489 - 504

Created: 01.05.2024 21:33 by jmlut, Modified: 01.05.2024 21:33 by jmlut

Area: 1157	2.63%

23.

"Und deswegen habe ich jetzt nicht das Gefühl, ohne es jemals ausprobiert zu haben, dass wenn ich jetzt zum Patienten sage und ihm einen Brief mitgebe, er sollte jetzt bis zur Operation, diese und jene Maßnahmen ergreifen, gehen Sie bitte zum Hausarzt, der soll sich darum kümmern. Und vielleicht hat er dann einen super Hausarzt, und ich weiß aber ehrlich
gesagt nicht, ob der die Möglichkeit hat, es überhaupt umzusetzen, ob das irgendjemand bezahlt."

*"And that's why I don't feel, without ever having tried it, that if I now tell the patient and give him a letter saying that he should take these and those measures until the operation, please go to your family doctor, who will take care of it. And maybe he has a great GP, but I honestly*

*don't know if he has the means to implement it at all, if anyone will pay for it."*

Code: ● Schnittstelle Intra- / Extramural	Weight score: 0

C1 200224, 526 - 533

Created: 01.05.2024 21:33 by jmlut, Modified: 01.05.2024 21:33 by jmlut

Area: 475	1.08%

24.

"Vielleicht geht das, da kenne ich mich zu wenig aus, muss ich ganz ehrlich sagen. Aber das wäre natürlich eigentlich das Wünschenswerte. Weil hier im Spital funktioniert es offensichtlich nicht, weil die spielen den Ball immer weiter, wir sind dafür nicht zuständig.
Aber ob die Möglichkeit überhaupt, ob das abgebildet ist im System, das kann ich dir ehrlich gesagt gar nicht sagen. Also, das..."

*"Maybe that's possible, but I have to be honest and say that I don't know enough about it. But that would of course be desirable. Because here in the hospital it obviously doesn't work, because they keep passing the buck and saying it's not their responsibility.*

*But whether that's even possible, whether it's reflected in the system, I honestly can't tell you. So, that..."*

Code: ● Schnittstelle Intra- / Extramural	Weight score: 0

C1 200224, 540 - 546

Created: 01.05.2024 21:34 by jmlut, Modified: 01.05.2024 21:34 by jmlut

Area: 418	0.95%

25.

"Das Ding, das wollen wir lange schauen sozusagen, aber das ist irgendwie immer wieder ein neuer Ansatz, immer wieder ein Gespräch, dann machst du Zuweisungen zu den Kollegen, und es passiert aber nichts."

*‘We want to look at this thing for a long time, so to speak, but it's always a new approach, always a new conversation, then you make assignments to your colleagues, and nothing happens.’*

Code: ● Schnittstelle Intra- / Extramural	Weight score: 0

C1 200224, 551 - 553

Created: 01.05.2024 21:34 by jmlut, Modified: 01.05.2024 21:34 by jmlut

Area: 205	0.47%

26.

"bei uns der Spitalsbereich der ist ,sozusagen, der soll sich kümmern, so mehr oder weniger und das ambulante oder das niedergelassene, da für mich persönlich zu wenig Rolle spielt und diese Vernetzung zu wenig stattfindet."

*‘In our hospital, it is, so to speak, the responsibility of the hospital to take care of this, more or less, and outpatient or private practice plays too small a role for me personally, and there is too little networking.’*

Code: ● Schnittstelle Intra- / Extramural	Weight score: 0

C1 200224, 667 - 670

Created: 01.05.2024 21:40 by jmlut, Modified: 01.05.2024 21:40 by jmlut

Area: 225	0.51%

27.

"wahrscheinlich würden sie sich's vorstellen, damit ich gut in die OP reingehe, gehe ich drei Wochen vorher in ein Krankenhaus, damit ich dann im Krankenhaus fit bin, so ungefähr, also man geht quasi auf eine Reha, sonst noch auf Preha und da werde ich dann vorbereitet eben, weil es eben auch nach der Operation nicht die ambulanten Strukturen gibt und diese
Behandlungen gibt, damit ich eben ambulant nach meiner Knieoperation oder nach meiner onkologischen Operation dort betreut werde im niedergelassenen Bereich, weil die werden dann alle eben irgendwo hingeschickt auf Reha sozusagen, wenn wir diese Strukturen nicht haben."

*"You can probably imagine that, in order to be in good shape for the operation, I go to a hospital three weeks beforehand so that I'm fit when I get there, sort of like going to rehab, or prehab, where I'm prepared for the operation because there aren't any outpatient facilities or treatments available after the operation*

*treatments available for me to be cared for on an outpatient basis after my knee surgery or my oncological surgery in the private practice sector, because they will all be sent somewhere for rehab, so to speak, if we don't have these structures."*

Code: ● Schnittstelle Intra- / Extramural	Weight score: 0

C1 200224, 686 - 694

Created: 01.05.2024 21:41 by jmlut, Modified: 01.05.2024 21:41 by jmlut

Area: 636	1.45%

28.

"dass wir da überhaupt kein Netzwerk haben in dem Sinn und da müsste es irgendwie eine andere Koordinationsstelle geben, wo ich dann einen Ansprechpartner hätte, wo ich weiß, okay, gut, das ist jetzt der Krebspatient eben und bitte kümmert euch um den, denn der hat dann und dann seine OP und dieses und jenes, sollte stattfinden.
Das hat vielleicht früher mit dem Hausarzt irgendwie funktioniert, aber auch da wirst du eigentlich den Eindruck haben, dass der auch hilflos ist, weil er eigentlich nicht die Möglichkeit hat, den irgendwo hinzuschicken und vermutlich auch nur spekulativ, je weiter du am Land bist, desto schwieriger wird es dann."

*"We don't have any kind of network in that sense, and there needs to be some kind of coordination centre where I would have a contact person and know, okay, this is the cancer patient, please take care of him, because he has his operation on such and such a date and this and that needs to happen.*

*That may have worked with the family doctor in the past, but even there you get the impression that he is helpless because he doesn't really have the option of sending him anywhere, and presumably the further out in the countryside you are, the more difficult it becomes."*

Code: ● Schnittstelle Intra- / Extramural	Weight score: 0

C1 200224, 701 - 710

Created: 01.05.2024 21:42 by jmlut, Modified: 01.05.2024 21:42 by jmlut

Area: 669	1.52%

29.

"Und dann, glaube ich, wird es auch ganz relevant sein, wie das bezahlt wird und wer das bezahlt. Weil dann je extramuraler das Ganze wird, desto weniger kann es quasi eine Umwegsrentabilität erlangen über eine kürzere Liegedauer oder wenige Komplikationen. Desto mehr wird derjenige der die Leistung erbringt, fragen, was er dafür bekommt und dann glaube ich, wird man sich da auch Finanzierungsmodelle überlegen müssen."

*" And then, I think it will also be very relevant how it is paid for and who pays for it. Because the more extramural the whole thing becomes, the less it can achieve indirect profitability through shorter hospital stays or fewer complications. The more the person providing the service will ask what they will get in return, and then I think financing models will also have to be considered."*

Code: ● Schnittstelle Intra- / Extramural	Weight score: 0

C2 180324, 323 - 328

Created: 20.05.2024 21:25 by Jan, Modified: 20.05.2024 21:25 by Jan

Area: 425	1.25%

30.

"ohne dass es da eine spezielle Art der Abrechnung gibt, ist es wahrscheinlich gar nicht so einfach umzusetzen, dass man sagt, okay gut, sie haben jetzt einen Operationstermin in fünf Wochen, wir brauchen innerhalb der nächsten 10 Tage einen Prähabilitationstermin für Sie,
das Sie da instruiert werden."

*"Without a specific billing method, it's probably not that easy to say, 'OK, fine, you have a surgery appointment in five weeks, we need a prehabilitation appointment for you within the next 10 days so that you can be instructed.'"*

Code: ● Schnittstelle Intra- / Extramural	Weight score: 0

C2 180324, 338 - 342

Created: 20.05.2024 21:25 by Jan, Modified: 20.05.2024 21:25 by Jan

Area: 306	0.90%

3.3. Unterschiedliche Träger

1.

"Also ich glaube, dass es immer noch daran scheitert, dass es eben keine Finanzierung aus einer Hand gibt. Dass sozusagen vom einen in der nächsten Topf immer das Geld weitergeschoben wird.
Diese Finanzierung aus einer Hand... Nur dann würde man nämlich auch wirklich nachweisen können, dass das ganze System und dass sowohl die Rehabilitation als auch die Prähabilitation wirklich kosteneffektiv ist. Sonst kann jeder statistisch rein und raus rechnen, kommt mir vor, wie er es gerade will."

*"Well, I think it still fails because there is no single source of funding. The money is always being passed from one pot to the next, so to speak.*

*This single source of funding... Only then would it be possible to really prove that the whole system, and that both rehabilitation and prehabilitation, is truly cost-effective. Otherwise, everyone can calculate their way in and out statistically, it seems to me, however they want."*

Code: ● Unterschiedliche Träger	Weight score: 0

K1 280224, 382 - 389

Created: 09.05.2024 20:37 by jmlut, Modified: 09.05.2024 20:37 by jmlut

Area: 513	1.38%

2.

"Das hat sich ja mit dem sehr sozusagen kleinen strukturierten Gesundheitssystem, viele verschiedene Träger in Österreich, aber auch Deutschland zu tun, während zum Beispiel in Skandinavien und auch UK das Gesundheitssystem sozusagen in nationaler Hand ist, beziehungsweise in großer öffentlicher Hand ist und von großen Trägern geleitet wird."

*‘This has to do with the very small, structured healthcare system, with many different providers in Austria, but also in Germany, whereas in Scandinavia and the UK, for example, the healthcare system is, so to speak, in national hands, or rather in large public hands, and is managed by large providers.’*

Code: ● Unterschiedliche Träger	Weight score: 0

A1 210224, 413 - 417

Created: 09.05.2024 17:33 by jmlut, Modified: 09.05.2024 17:33 by jmlut

Area: 346	0.87%

3.

"Das heißt nicht, dass hier schlechte Medizin gemacht wird, das heißt einfach, dass nachdem alles sozusagen deutlich klein strukturierter ist und viele Häuser sozusagen in kleinen Betrieben sozusagen arbeiten oder überhaupt als Einzelbetrieb arbeiten, wenn man zum Beispiel die Universitätskliniker hernimmt, die stehen als ein allein Silo, die Ordenshäuser, die sind auch nicht eine Gruppe, sondern gibt es auch verschiedene Träger, die halt intern irgendwelche Richtlinien machen."

*" That doesn't mean that the medicine practised here is poor; it simply means that everything is much more small-scale, so to speak, and many institutions operate as small businesses, or even as individual businesses. If you take university hospitals, for example, they stand alone as silos, and religious institutions are not a group either, but rather there are various sponsors who simply set internal guidelines."*

Code: ● Unterschiedliche Träger	Weight score: 0

A1 210224, 440 - 446

Created: 09.05.2024 17:35 by jmlut, Modified: 09.05.2024 17:35 by jmlut

Area: 487	1.23%

4.

"Da ist einfach diese, die Dauer sozusagen gemeinsam etwas auf die Beine zu bringen, ist einfach nicht gegeben, weil es einfach so klein strukturiert ist. Weil es nicht einen Ansprechpartner gibt, sondern wahrscheinlich mehr als 50 verschiedene Träger gibt, in ganz Österreich die Krankenhäuser führen. Und die dann intern irgendwelche eigenen Guidelines haben, aber sozusagen nicht an einer... sozusagen die alle zusammenzubringen, für eine nationale Guideline zusammenzukriegen, das ist ganz schwierig. Als Beispiel kann man sagen, gibt es ja auch neun verschiedene, für die neun Bundesländer, neun Ethikkommissionen.
Wenn man eine Studie für neun Millionen Einwohner machen will, das gibt schon ein bisschen einen Eindruck, wie schwierig es dann ist, sozusagen für diese neun Millionen Einwohner ein Konzept für alle auszurollen, weil welcher Träger soll dann auch stärker sein, also dominanter sein, die anderen 50, sagen wir mal, die 55 plus dominieren, um sein ERAS oder halt Prähabilitätskonzept auszurollen."

*"There is simply no way to get something off the ground together, so to speak, because it is structured on such a small scale. Because there is no single point of contact, but probably more than 50 different institutions that run hospitals throughout Austria. And they all have their own internal guidelines, but it's very difficult to bring them all together to create a national guideline. As an example, there are nine different ethics committees for the nine federal states.*

*If you want to conduct a study for nine million inhabitants, that gives you some idea of how difficult it is to roll out a concept for all of these nine million inhabitants, because which institution should then be stronger, i.e. more dominant, than the other 50, let's say the 55 plus, in order to roll out its ERAS or prehabilitation concept."*

Code: ● Unterschiedliche Träger	Weight score: 0

A1 210224, 448 - 461

Created: 09.05.2024 17:57 by jmlut, Modified: 09.05.2024 17:57 by jmlut

Area: 1043	2.63%

5.

"Das sehe ich, also habe ich in den letzten Jahren, und gerade auch in Covid halt gesehen, ist nach meiner Wahrnehmung ein sehr großes Problem, also diese kleinparzellige, siloartige Zerstückelung in der österreichischen Krankenhauslandschaft."

*‘I see that, so I have seen in recent years, and especially during Covid, that in my opinion there is a very big problem, namely this small-scale, silo-like fragmentation in the Austrian hospital landscape.’*

Code: ● Unterschiedliche Träger	Weight score: 0

A1 210224, 463 - 466

Created: 09.05.2024 17:58 by jmlut, Modified: 09.05.2024 17:58 by jmlut

Area: 245	0.62%

6.

"Dann gibt es natürlich zahlreiche kommunale Träger nach wie vor und Landeshäuser, Organisationen, Träger, also jedes Bundesland hat einen eigenen Krankenhausträger, also im Landesbesitz. Die haben, die sind irgendwie so eine Mischung zwischen ein bisschen Forschung, ein bisschen Innovation, die tun sich zum Teil ganz schwer und da hängt es dann häufig an einzelnen Köpfen in jeweiligen Kliniken, ob da jetzt noch was speziell gepusht wird oder nicht, aber ich glaube, damit hast du schon ein bisschen Eindruck bekommen, dass das eine sehr zerklüftete Krankenhauslandschaft ist, wo man nicht ein Konzept für alle ausholen kann."

*"Then, of course, there are still numerous municipal institutions and state houses, organisations, institutions, so each federal state has its own hospital institution, i.e. state-owned. They are a mixture of a little research, a little innovation, and some of them are struggling, so it often depends on individual minds in the respective clinics whether something specific is being pushed or not, but I think that gives you a bit of an impression that it's a very fragmented hospital landscape where you can't come up with one concept for everyone."*

Code: ● Unterschiedliche Träger	Weight score: 0

A1 210224, 486 - 494

Created: 09.05.2024 17:59 by jmlut, Modified: 09.05.2024 17:59 by jmlut

Area: 636	1.60%

7.

"Auch hier ist aber die Frage dann wieder, was sind die low-hanging Foods? Weil low-hanging ist sicher, man macht es in der eigenen Abteilung oder im eigenen Konzern und die Frage ist dann wirklich, was bringt es, wenn ich es jetzt auf andere Konzerne ausrolle?
Was bringt es mir? Also werde ich da überhaupt freigestellt. Was sagt mein Arbeitgeber dazu? Wenn ich beim Arbeitgeber A oder Konzern A versuche, die Konzerne B bis Z zu überreden, das sie das machen. Will das überhaupt der Arbeitgeber A? Dass seine Konkurrenten da besser werden. Da sind sicher andere Systeme, wo es nur einen Arbeitgeber A gibt, zum Beispiel NHS oder das dänische oder norwegische System sind da sicher einfacher gestrickt, weil da macht man es in Krankenhaus A, bis auch ins Krankenhaus Z dann gleich,
und es ist dann immer der Arbeitgeber der gleiche, soweit ich das System halt kenne, also im Großen und Ganzen, die konkurrieren in keinster Weise."

*"But here too, the question arises again: what are the low-hanging foods? Because low-hanging is safe, you do it in your own department or in your own company, and the question is really, what is the point of rolling it out to other companies?*

*What's in it for me? So am I even allowed to do that? What does my employer say about it? If I try to persuade companies B to Z to do it while working for employer A or group A. Does employer A even want that? For its competitors to get better at it. There are certainly other systems where there is only one employer A. For example, the NHS or the Danish or Norwegian systems are certainly simpler because you do it in hospital A, then in hospital Z,*

*and it's always the same employer, as far as I know the system, so on the whole, they don't compete in any way."*

Code: ● Unterschiedliche Träger	Weight score: 0

A1 210224, 654 - 668

Created: 09.05.2024 18:05 by jmlut, Modified: 09.05.2024 18:05 by jmlut

Area: 976	2.46%

8.

"Selbstverständlich um Wirtschaftlichkeit, das ist schon ein Thema, aber nicht nur. Und Patienten optimal durch große Operationen durchzuführen mit einem sehr guten postoperativen Outcome, kann nur im Interesse der Politik liegen, beziehungsweise des Krankenhausbetreibers, wo es ja in Österreich sehr gesplittet ist.
Wir haben ja viele verschiedene Betreiber, ja, öffentlich, Bund, Land, Stadt, ganz privat, je nachdem. Aber es liegt sicher im Sinne des Betreibers zu sagen, hört einmal, dass ist ein ganz wichtiger Aspekt, der wird, unsere Kosten, unseren Personalbedarf, den Pflegebedarf des Patienten reduzieren, wenn man vorher besser drauf schaut."

*"Of course, economic efficiency is an issue, but it's not the only one. And performing major operations on patients with optimal results and very good postoperative outcomes can only be in the interest of politicians and hospital operators, which are very divided in Austria.*

*We have many different operators, yes, public, federal, state, city, completely private, depending on the situation. But it is certainly in the operator's interest to say, listen, this is a very important aspect, which will reduce our costs, our staffing requirements and the patient's care needs if we take a closer look at it beforehand."*

Code: ● Unterschiedliche Träger	Weight score: 0

A2 220422, 239 - 249

Created: 09.05.2024 19:42 by jmlut, Modified: 09.05.2024 19:42 by jmlut

Area: 678	1.90%

4. Wirtschaftliche Faktoren

4.1. Wirtschaftliche Faktoren Land / Bund / Kasse

1.

"aus der Sicht des Kostenträgers dürfte es wahrscheinlich ein ähnlich gelagertes Problem sein, nämlich das Krankenhaus sagt, diese Sachen, die alle vor der Operation stehen, gerade bei elektiven Eingriffen gehen uns ja nichts an, sondern das ist ein Thema des niedergelassenen Bereichs und der niedergelassene Bereich sagt, na ja, das steht ja unmittelbar mit der bevorstehenden Operation, die im Bereich des Spitals stattfindet im Zusammenhang, deswegen geht es uns nichts an."

*" From the perspective of the cost bearer, it is likely to be a similar problem, namely that the hospital says that these matters, which all arise before the operation, particularly in the case of elective procedures, are not our concern, but rather a matter for private practitioners, and the private practitioners say that, well, it is directly related to the upcoming operation, which takes place in the hospital, so it is not our concern. "*

Code: ● Wirtschaftliche Faktoren Land / Bund / Kasse	Weight score: 0

S1 080324, 63 - 70

Created: 20.05.2024 16:57 by Jan, Modified: 20.05.2024 16:57 by Jan

Area: 483	1.85%

2.

"Und dazu braucht es natürlich Geld. Wir als Sozialversicherung haben keinen Auftrag, und können aus gesetzlichen Gründen heraus auch nicht, in Feldforschung oder in klinische Forschung, in dem Fall, investieren. Für so eine Studie müsste man wahrscheinlich wirklich irgendwo im Forschungsressort, im Wissenschaftsressort schauen, was es da für Gelder gibt.
Es gibt ja genügend oder reichlich Töpfe im Wissenschaftsbereich, wo wahrscheinlich solche Mittel sind."

*"And of course, that requires money. As a social insurance provider, we have no mandate to invest in field research or clinical research in this case, nor are we legally permitted to do so. For a study like this, you would probably have to look somewhere in the research department, in the science department, to see what funds are available.*

*There are plenty of pots of money in the science sector where such funds are likely to be found."*

Code: ● Wirtschaftliche Faktoren Land / Bund / Kasse	Weight score: 0

S1 080324, 148 - 155

Created: 20.05.2024 17:01 by Jan, Modified: 20.05.2024 17:01 by Jan

Area: 483	1.85%

3.

"der Spitalsträger bräuchte aus meiner Sicht eine Abrechnungsposition dafür. Also, die sie kodieren, wenn sie die Patienten sehen, müssten sie eine Abrechnungsposition kodieren. Und dann bräuchte es aus meiner Sicht auch noch, aber das ist Gegenstand von so einem Standard oder von einer Richtlinie, irgendeinen patientenführenden Arzt auch für das Ganze."

*‘In my opinion, the hospital operator would need a billing item for this. So, when they see the patients, they would have to code a billing item. And then, in my opinion, it would also be necessary, but that is the subject of a standard or guideline, to have a doctor who is responsible for the patient for the whole thing.’*

Code: ● Wirtschaftliche Faktoren Land / Bund / Kasse	Weight score: 0

S1 080324, 202 - 206

Created: 20.05.2024 17:04 by Jan, Modified: 20.05.2024 17:04 by Jan

Area: 358	1.37%

4.

"Wir haben in Österreich, Gott sei Dank, eine sehr flächendeckende Sachleistungsversorgung,
die nach dem Prinzip der Bedarfsgerechtigkeit funktioniert. Und wenn man jetzt in andere Länder schaut, kann es natürlich sein, dass, weiß ich nicht, sowas bei den USA bei der Kaiser Permanente zum Beispiel umgesetzt wird. Die haben zwar auch eine achleistungsversorgung, die Kaiser Permanente, aber das ist ein Single Payer Model.
Und die haben wahrscheinlich auch ihre eigenen Krankenhäuser, die haben sich das durchgerechnet und kommen dann wahrscheinlich drauf, das ist irgendwie was, was sich für sie rechnet und gleichzeitig vielleicht auch was ist, was sie irgendwo als Marketing bei den Patienten gut einsetzen können. Und diese Dinge haben wir halt nicht."

*"Thankfully, in Austria we have a very comprehensive system of benefits in kind,*

*which operates on the principle of needs-based provision. And if you look at other countries, it may well be that something similar is being implemented in the USA at Kaiser Permanente, for example. Kaiser Permanente also has a benefits in kind system, but it is a single payer model.*

*And they probably also have their own hospitals, they've done the math and probably come to the conclusion that it's something that pays off for them and at the same time is something they can use as marketing to patients. And we just don't have those things."*

Code: ● Wirtschaftliche Faktoren Land / Bund / Kasse	Weight score: 0

S1 080324, 271 - 285

Created: 20.05.2024 17:13 by Jan, Modified: 20.05.2024 17:13 by Jan

Area: 818	3.14%

5.

"Das ist überall dort, wo halt die Brüche zwischen den Kostenträgern sind, ja in diesem Schnittstellenbereich zwischen intra- und extramuraler Versorgung. Da wird es schwierig, weil die Logik ist ja die, wenn die Prähabilitation im niedergelassenen Bereich stattfindet, spart sich der Krankenanstaltenträger was. Aber die Sozialversicherung kostet es was. Und da ist halt das Anreizsystem nicht so vorhanden, um solche Dinge aufzunehmen in einen Leistungskatalog."

*"This is wherever there are gaps between cost bearers, i.e. in this interface area between inpatient and outpatient care. This is where it gets difficult, because the logic is that if prehabilitation takes place in private practice, the hospital operator saves money. But it costs the social security system money. And there is no incentive system in place to include such things in a catalogue of services."*

Code: ● Wirtschaftliche Faktoren Land / Bund / Kasse	Weight score: 0

S1 080324, 298 - 305

Created: 20.05.2024 17:14 by Jan, Modified: 20.05.2024 17:14 by Jan

Area: 486	1.87%

6.

"Die Töpfe - aus dem Gesundheitssystem heraus, haben wir eigentlich gar keine wirklichen Töpfe, wo es zur klinischen Forschung oder sowas Mittel gibt, ja, das sind wirklich FWF -Mittel, also vom Wissenschaftsförderungsfonds - ich würde das eher irgendwo in diesem Bereich sehen"

*‘The funds – outside of the healthcare system, we don't really have any funds for clinical research or anything like that. Yes, there are FWF funds, i.e. from the Science Promotion Fund – I would see that more as something in this area.’*

Code: ● Wirtschaftliche Faktoren Land / Bund / Kasse	Weight score: 0

S1 080324, 354 - 357

Created: 20.05.2024 17:19 by Jan, Modified: 20.05.2024 17:19 by Jan

Area: 279	1.07%

7.

"leistungsrechtlich sind nicht alle Dinge, die jetzt dort in dem kleinen Konzept das sie mitgeschickt haben, in Österreich, in der Sachleistungsversorgung enthalten, wie zum Beispiel Fitnessstudios. Bei uns, wir bezahlen ja Krankenbehandlung und das ist irgendwie, wenn man es denn gesundheitsmäßig fassen möchte, wäre das allenfalls Gesundheitsförderung, ja, aber das ist nicht wirklich eine Leistung im österreichischen Gesundheitswesen, auch die ErnährungsberaterInnen - bei uns dürfen ja, soweit ich informiert bin, nur DiätologInnen an Patienten arbeiten, ja, und eben auch diese sind ja dann
nur irgendwo in der Sachleistungsversorgung tätig. Also die ErnährungswissenschaftlerInnen sind bei uns... können sich um gesunde Menschen kümmern, aber nicht um Kranke."

*"In terms of benefits law, not everything included in the brief outline you sent us is covered by benefits in kind in Austria, such as gym membership. Here, we pay for medical treatment, and if you want to look at it from a health perspective, that would be health promotion, yes, but it's not really a service in the Austrian healthcare system. The same goes for nutritionists – as far as I know, only dieticians are allowed to work with patients here, and even they are*

*only involved in benefits in kind. So nutritionists here... can look after healthy people, but not sick people."*

Code: ● Wirtschaftliche Faktoren Land / Bund / Kasse	Weight score: 0

S1 080324, 401 - 412

Created: 20.05.2024 17:20 by Jan, Modified: 20.05.2024 17:20 by Jan

Area: 794	3.05%

8.

"Mir ging es um die Gesundheitsberufe, dass die Ernährungswissenschaftlerin, das ist ja ein Universitätsstudium, glaube ich, das ist kein Gesundheitsberuf, der bei uns in der Sachleistungsversorgung tätig sind, das sind die Diätologinnen, fragen Sie mich nicht, wo die Unterschiede da sind. Ich habe das mal so gelernt, die Diätologie ist etwas anderes als die
Ernährungswissenschaft. Und die Sportmedizin haben wir auch nirgends drin, sondern bei uns, wir zahlen nur für die Physiotherapie."

*"I was referring to the health professions, that nutritionists, which is a university degree, I believe, are not health professionals who work in our benefits in kind system; those are dieticians. Don't ask me what the differences are. That's how I learned it: dietetics is different from nutrition science. And we don't have sports medicine anywhere either; we only pay for physiotherapy."*

Code: ● Wirtschaftliche Faktoren Land / Bund / Kasse	Weight score: 0

S1 080324, 421 - 428

Created: 20.05.2024 17:21 by Jan, Modified: 20.05.2024 17:21 by Jan

Area: 514	1.97%

9.

"Die müssen ein gewisses Programm haben, sonst kriegen ja gar nicht die Zertifizierung dafür."

*‘They must have a certain programme, otherwise they won't get certified for it.’*

Code: ● Wirtschaftliche Faktoren Land / Bund / Kasse	Weight score: 0

PT1 140324, 336 - 337

Created: 20.05.2024 20:08 by Jan, Modified: 20.05.2024 20:08 by Jan

Area: 93	0.26%

10.

"Und wenn man da vorrechnen kann, das bringt so viel, weil du nachher einen weniger langen Aufenthalt in der Intensiv hast, also das müsste man nachher anders aufschlüsseln und so in der Schiene erklären, weil sonst interessiert das keinen Menschen."

*‘And if you can calculate that it brings so much, because you end up spending less time in intensive care, then you would have to break it down differently and explain it in that way, because otherwise no one will be interested.’*

Code: ● Wirtschaftliche Faktoren Land / Bund / Kasse	Weight score: 0

PT1 140324, 392 - 395

Created: 20.05.2024 20:10 by Jan, Modified: 20.05.2024 20:10 by Jan

Area: 251	0.70%

11.

"Naja, weil so lange es nicht um Geld geht, ist es uninteressant, glaube ich."

*‘Well, as long as it's not about money, I don't think it's interesting.’*

Code: ● Wirtschaftliche Faktoren Land / Bund / Kasse	Weight score: 0

PT1 140324, 399 - 399

Created: 20.05.2024 20:10 by Jan, Modified: 20.05.2024 20:10 by Jan

Area: 76	0.21%

12.

"das du halt sowohl die Politik als auch halt diese Sozialversicherungsträger auf deine Seite bringst, so dass das nachher bezahlt wird oder dass die Leute das lukrieren können, weil sonst glaube ich selbst zahlen, wie gesagt, ist in Österreich ganz mau, finde ich."

*‘That you get both politicians and social security providers on your side, so that it gets paid for afterwards or that people can profit from it, because otherwise I think paying for it yourself, as I said, is pretty poor in Austria, in my opinion.’*

Code: ● Wirtschaftliche Faktoren Land / Bund / Kasse	Weight score: 0

PT1 140324, 411 - 414

Created: 20.05.2024 20:11 by Jan, Modified: 20.05.2024 20:11 by Jan

Area: 267	0.75%

13.

"Wahrscheinlich ist das auch ein Grund, wer finanziert sowas. Natürlich, es gibt kein Konzept dafür, das ist sicher auch der Grund, also wahrscheinlich muss man da mal mit den Kassen reden."

*‘That's probably also a reason why no one is financing it. Of course, there's no concept for it, which is certainly also the reason, so you probably have to talk to the health insurance funds about it.’*

Code: ● Wirtschaftliche Faktoren Land / Bund / Kasse	Weight score: 0

P1 010324, 222 - 224

Created: 20.05.2024 18:29 by Jan, Modified: 20.05.2024 18:29 by Jan

Area: 190	0.50%

14.

"Genau, also wenn mir jemand vor der Operation wen zuweist, kann ich mich mit dem
zusammensetzen. Aber wenn der noch gar nicht stationär ist, dann nicht. Dann müsste man ein Konzept überlegen, dass der rein darf, dass der ambulant rein darf."

*"Exactly, so if someone assigns me someone before the operation, I can sit down with them.*

*But if they are not yet hospitalised, then no. In that case, we would have to come up with a plan to allow them to come in as an outpatient."*

Code: ● Wirtschaftliche Faktoren Land / Bund / Kasse	Weight score: 0

P1 010324, 229 - 232

Created: 20.05.2024 18:46 by Jan, Modified: 20.05.2024 18:46 by Jan

Area: 243	0.63%

15.

"Bürokratische Hürden vielleicht, finanzielle Hürden. Bei uns jetzt eben Bewusstsein. Ein klarer Auftrag, wirtschaftliche Hürden. Also die Frage ist, ob das wirtschaftlich ist. Das musst du der [entfernt] schon erstmal gut verkaufen.
Das muss gezahlt werden, das muss Punkte bringen. Das wird der Kasse - ich weiß nicht, ob das Punkte bringt. Wir behandeln, wir operieren, aber nicht vorher schon. Das ist eben genau das. Wie viel steckt man Geld in vorher, in Gesundheitserhaltung, in Prävention?"

*"Bureaucratic hurdles perhaps, financial hurdles. For us right now, it's awareness. A clear mandate, economic hurdles. So the question is whether it's economical. You have to sell that to the [removed] first.*

*It has to be paid for, it has to earn points. That will cost money – I don't know if it will earn points. We treat, we operate, but not beforehand. That's exactly it. How much money do you put into prevention, into maintaining health?"*

Code: ● Wirtschaftliche Faktoren Land / Bund / Kasse	Weight score: 0

P1 010324, 569 - 576

Created: 20.05.2024 18:56 by Jan, Modified: 20.05.2024 18:56 by Jan

Area: 521	1.36%

16.

"Bisher natürlich noch wenig, weil es nicht bezahlt wurde, wir haben natürlich im Rahmen der ambulanten Rehabilitation immer wieder auch davon gesprochen, dass das ein guter Ansatz wäre um die Patienten vorzeitig auch schon fit für operative Eingriffe, bei uns vor allem kardiologische Eingriffe, die ja doch immer intensiver, größer oft einmal mit Herzlungenmaschine und Nierenproblematik verbunden sind, Anämieproblematik verbunden sind, oft multimorbide Patienten. Das wurde besprochen, aber meistens noch nicht in der Realität umgesetzt, sondern ich selbst, wir haben keine Präehabilitation gemacht, sondern *vor allem Rehabilitation."*

*"So far, of course, very little, because it wasn't paid for. We have, of course, repeatedly discussed in the context of outpatient rehabilitation that this would be a good approach to getting patients fit for surgery ahead of time, in our case mainly cardiological procedures, which are always more intensive, often larger, and often involve heart-lung machines and kidney problems, anaemia problems, and often multimorbid patients. This has been discussed, but in most cases it has not yet been implemented in reality. Instead, we have not done any prehabilitation, but mainly rehabilitation."*

Code: ● Wirtschaftliche Faktoren Land / Bund / Kasse	Weight score: 0

K1 280224, 132 - 140

Created: 09.05.2024 20:27 by jmlut, Modified: 09.05.2024 20:27 by jmlut

Area: 644	1.74%

17.

"Darum der Wille und der Wunsch zusammenzuarbeiten in der Prähabilitation war natürlich da, weil der Chirurg auch davon profitiert hätte, und der Anästhesist vor allem, aber es ist wie immer ums Geld gegangen, dass das nicht bezahlt wurde."

*‘That's why the will and desire to work together in prehabilitation was naturally there, because the surgeon would also have benefited from it, and the anaesthetist in particular, but as always, it was about money, that it wasn't paid for.’*

Code: ● Wirtschaftliche Faktoren Land / Bund / Kasse	Weight score: 0

K1 280224, 184 - 186

Created: 09.05.2024 20:28 by jmlut, Modified: 09.05.2024 20:28 by jmlut

Area: 240	0.65%

18.

"Und ja, es ist ein fürchterlicher Kampf. Damals schon, und ich mir vorstellen kann, dass das jetzt auch nicht besser geworden ist, aber wenn man mit Fakten argumentieren kann, dass es international eventuell etwas gibt, dass das Ganze billiger wird, wenn man die Leute gut vorbereitet. Das ist glaube ich das einzige Argument. Leider geht es immer ums Geld und das,
was man hineinsteckt, muss für das Gesundheitssystem irgendwie wieder wenigstens
1 zu 1 zurückkommen oder sogar günstiger sein."

*"And yes, it's a terrible struggle. It was back then, and I can imagine that it hasn't gotten any better now, but if you can argue with facts that there may be something internationally that makes the whole thing cheaper if you prepare people well. I think that's the only argument. Unfortunately, it's always about money, and what you put in has to somehow come back at least 1 to 1 for the healthcare system, or even be cheaper."*

Code: ● Wirtschaftliche Faktoren Land / Bund / Kasse	Weight score: 0

K1 280224, 357 - 364

Created: 09.05.2024 20:36 by jmlut, Modified: 09.05.2024 20:36 by jmlut

Area: 516	1.39%

19.

"Also ich glaube, dass es immer noch daran scheitert, dass es eben keine Finanzierung aus einer Hand gibt. Dass sozusagen vom einen in der nächsten Topf immer das Geld weitergeschoben wird. Diese Finanzierung aus einer Hand... Nur dann würde man nämlich auch wirklich nachweisen können, dass das ganze System und dass sowohl die Rehabilitation als auch die Prähabilitation wirklich kosteneffektiv ist. Sonst kann jeder statistisch rein und raus rechnen, kommt mir vor, wie er es gerade will."

*"Well, I think it still fails because there is no single source of funding. The money is always being passed from one pot to the next, so to speak. This single source of funding... Only then would it be possible to really prove that the whole system and that both rehabilitation and prehabilitation are truly cost-effective. Otherwise, everyone can calculate statistically as they please, it seems to me."*

Code: ● Wirtschaftliche Faktoren Land / Bund / Kasse	Weight score: 0

K1 280224, 382 - 389

Created: 09.05.2024 20:37 by jmlut, Modified: 09.05.2024 20:37 by jmlut

Area: 513	1.38%

20.

"Also diese Finanzierung aus einer Hand, die ist in Österreich eben überhaupt nicht. Diese vielen verschiedenen Versicherungsträger, wo jeder sein Klientel hat, das ein bisschen andere Ansprüche hat und andere Selbstbehalte und und und, also auch die verschiedenen Versicherungssysteme, die da abzudecken sind und verschiedene Begehrlichkeiten und auch natürlich verschiedene politische Einflussnahmen, weil ja die Versicherungen auch eben je nachdem politisch besetzt sind, das ist auch ein Problem."

*‘So this single-source financing is simply not available in Austria. There are so many different insurance providers, each with their own clientele, who have slightly different requirements and different deductibles, and so on. Then there are the different insurance systems that need to be covered, different desires and, of course, different political influences, because insurance companies are also politically involved, depending on the circumstances. That's also a problem.’*

Code: ● Wirtschaftliche Faktoren Land / Bund / Kasse	Weight score: 0

K1 280224, 391 - 397

Created: 09.05.2024 20:37 by jmlut, Modified: 09.05.2024 20:37 by jmlut

Area: 505	1.36%

21.

"Ich glaube auch, dass das Problem dieser Schnittstellen zwischen extramuralem / intramuralem Bereich ein Problem ist, dass man da zu wenig ökonomisch auch denkt und die Untersuchungen zu häufig wiederholt und dadurch sehr viele sowohl zeitliche Lehrläufe, also auch finanzielle Lehrläufe und zusätzliche Kosten entstehen, die nicht notwendig wären."

*‘I also believe that the problem of these interfaces between the extramural and intramural sectors is that there is too little economic thinking and that examinations are repeated too often, resulting in a great deal of unnecessary time, financial resources and additional costs.’*

Code: ● Wirtschaftliche Faktoren Land / Bund / Kasse	Weight score: 0

K1 280224, 399 - 403

Created: 09.05.2024 20:38 by jmlut, Modified: 09.05.2024 20:38 by jmlut

Area: 352	0.95%

22.

"Genau, also diese einheitliche Trägerschaft, und wie immer wir es jetzt nennen, sozusagen Finanzierung aus einer Hand, dass man einen Topf hat und man auch gegenrechnen kann, welche Dinge, wieviel fließt jetzt genau in den niedergelassenen Bereich, wieviel fließt in die Krankenhäuser. Das ist dieses Mischsystem, das wir hier haben, das sagt ja auch jeder Gesundheitsökonom, dass das einfach sehr viele Leerläufe und sehr viele finanzielle Fehlflüsse auch zulässt. Und dann diese große Abtrennung des privatärztlichen, der zusatzversicherten Patienten."

*"Exactly, so this uniform sponsorship, and whatever we call it now, financing from a single source, so to speak, means that you have one pot and you can also calculate exactly how much goes to the private practice sector and how much goes to the hospitals. That's the mixed system we have here, and every health economist says that it simply allows for a lot of idle capacity and a lot of financial misallocation. And then there's this big separation between private patients and those with supplementary insurance."*

Code: ● Wirtschaftliche Faktoren Land / Bund / Kasse	Weight score: 0

K1 280224, 421 - 429

Created: 09.05.2024 20:38 by jmlut, Modified: 09.05.2024 20:38 by jmlut

Area: 577	1.55%

23.

"Soweit meine Wissen sind wir jetzt in den österreichischen Gesundheitssystem noch nicht. Das ist eine wie die Physiotherapie, präoperativ oder generell, da weiß ich jetzt nicht genau, wie das verrechnet wird. Meines Wissens gibt es da noch nichts, wo man jetzt das Präoperative separat extra auch noch verrechnen könnte im Sinn von, ich habe da jetzt noch eine zusätzliche Leistung gebracht, die noch separat sozusagen in das Budget als
Einnahme reinfließt."

*"As far as I know, we are not there yet in the Austrian healthcare system. It's like physiotherapy, preoperative or general, I'm not sure exactly how it's billed. To my knowledge, there is nothing yet where you could bill the preoperative separately in the sense that I have now provided an additional service that flows separately into the budget as*

*income, so to speak."*

Code: ● Wirtschaftliche Faktoren Land / Bund / Kasse	Weight score: 0

A1 210224, 142 - 149

Created: 09.05.2024 17:14 by jmlut, Modified: 09.05.2024 17:14 by jmlut

Area: 480	1.21%

24.

"Und jetzt müssen aus meiner Sicht, wie gesagt, noch die Strukturen geschaffen werden, um die Prähabilitation halt auch möglich zu machen."

*‘And now, in my opinion, as I said, the structures still need to be created to make prehabilitation possible.’*

Code: ● Wirtschaftliche Faktoren Land / Bund / Kasse	Weight score: 0

A1 210224, 156 - 157

Created: 09.05.2024 17:16 by jmlut, Modified: 09.05.2024 17:16 by jmlut

Area: 138	0.35%

25.

"Abgerechnet wird eigentlich noch gar nichts. Also es läuft jetzt mal als Pilot, wo man mal sozusagen das Ganze auch dann wissenschaftlich natürlich begleitet."

*‘Nothing is actually being billed yet. So it's running as a pilot project at the moment, where the whole thing is being scientifically monitored, so to speak.’*

Code: ● Wirtschaftliche Faktoren Land / Bund / Kasse	Weight score: 0

A1 210224, 162 - 163

Created: 09.05.2024 17:16 by jmlut, Modified: 09.05.2024 17:16 by jmlut

Area: 159	0.40%

26.

"Und wenn man so ein Projekt flächendeckend durchziehen will, wird man irgendwo belegen müssen, dass das wirtschaftlich interessant ist."

*‘And if you want to implement such a project across the board, you will have to prove somewhere that it is economically viable.’*

Code: ● Wirtschaftliche Faktoren Land / Bund / Kasse	Weight score: 0

A2 220422, 224 - 225

Created: 09.05.2024 19:41 by jmlut, Modified: 09.05.2024 19:41 by jmlut

Area: 136	0.38%

27.

"Selbstverständlich um Wirtschaftlichkeit, das ist schon ein Thema, aber nicht nur. Und Patienten optimal durch große Operationen durchzuführen mit einem sehr guten postoperativen Outcome, kann nur im Interesse der Politik liegen, beziehungsweise des Krankenhausbetreibers, wo es ja in Österreich sehr gesplittet ist. Wir haben ja viele verschiedene Betreiber, ja, öffentlich, Bund, Land, Stadt, ganz privat, je nachdem. Aber es liegt sicher im Sinne des Betreibers zu sagen, hört einmal, dass ist ein ganz wichtiger Aspekt, der wird, unsere Kosten, unseren Personalbedarf, den Pflegebedarf des Patienten reduzieren, wenn man vorher besser drauf schaut."

*"Of course, economic efficiency is an issue, but it's not the only one. And performing major operations on patients with optimal results and very good postoperative outcomes can only be in the interest of politicians and hospital operators, where there is a great deal of division in Austria. We have many different operators, yes, public, federal, state, city, completely private, depending on the situation. But it is certainly in the operator's interest to say, listen, this is a very important aspect, which will reduce our costs, our staffing requirements and the patient's care needs if we take a closer look at it beforehand."*

Code: ● Wirtschaftliche Faktoren Land / Bund / Kasse	Weight score: 0

A2 220422, 239 - 249

Created: 09.05.2024 19:42 by jmlut, Modified: 09.05.2024 19:42 by jmlut

Area: 678	1.90%

28.

"Dann kann ich mir vorstellen, wird das machbar sein und man muss sich einfach Dinge überlegen, was in Zukunft wie passieren soll, weil der Personalmangel eklatant werden wird, aber auch die gesammte Wirtschaftssituation sich verändern wird in den nächsten Jahren.
Und man muss überlegen, wir können wir das personalmäßig stemmen, wir können wir das wirtschaftlich stemmen und wo können wir Kosten einsparen."

*"Then I can imagine that it will be feasible and we simply have to think about what should happen in the future, because the shortage of personnel will become glaring, but also because the overall economic situation will change in the coming years.*

*And we have to consider how we can manage this in terms of personnel, how we can manage it economically and where we can save costs."*

Code: ● Wirtschaftliche Faktoren Land / Bund / Kasse	Weight score: 0

A2 220422, 251 - 257

Created: 09.05.2024 19:43 by jmlut, Modified: 09.05.2024 19:43 by jmlut

Area: 429	1.20%

29.

"Und man muss dafür auch finanzielle Mittel zur Verfügung stellen, weil es kann ja nicht sein, dass man das in den niedergelassenen Bereich auslagert, aber die Kollegen und Kolleginnen dafür auch nicht entlohnt werden."

*‘And you also have to provide the financial resources for this, because you can't outsource it to private practices without remunerating your colleagues for it.’*

Code: ● Wirtschaftliche Faktoren Land / Bund / Kasse	Weight score: 0

A2 220422, 350 - 352

Created: 09.05.2024 19:46 by jmlut, Modified: 09.05.2024 19:46 by jmlut

Area: 219	0.61%

30.

"Man kann Ressourcen generieren und die vorhandenen kann man vielleicht auch schonender damit behandeln, oder ja, weil sie... je weniger Komplikationen, umso besser operiert der Patient postoperativ ist, umso angenehmer, umso leichter ist er auch zu pflegen und zu handlen. Nicht immer, aber sehr oft, es geht letztendlich immer nur um die Kosten."

*‘You can generate resources and perhaps treat the existing ones more carefully, or yes, because they... the fewer complications, the better the patient's postoperative condition, the more comfortable, the easier they are to care for and handle. Not always, but very often, it ultimately always comes down to the costs.’*

Code: ● Wirtschaftliche Faktoren Land / Bund / Kasse	Weight score: 0

A2 220422, 544 - 549

Created: 09.05.2024 19:51 by jmlut, Modified: 09.05.2024 19:51 by jmlut

Area: 366	1.03%

31.

"Und ich hab auch keine Ahnung, muss ich ganz ehrlich sagen, ob der das überhaupt umsetzen könnte, ob es da überhaupt von der Vergütung gibt oder was auch immer. Also das muss ich gestehen, muss ich passen sozusagen."

*‘And I have no idea, to be honest, whether he could even implement it, whether there is any remuneration for it or whatever. So I have to admit, I have to pass, so to speak.’*

Code: ● Wirtschaftliche Faktoren Land / Bund / Kasse	Weight score: 0

C1 200224, 264 - 266

Created: 01.05.2024 16:33 by jmlut, Modified: 01.05.2024 16:33 by jmlut

Area: 217	0.49%

32.

"In Österreich ist ja das Spitalsystem und das Niedergelassenensystem getrennt, sind zwei unterschiedliche Töpfe und in dem Moment, und das ist, glaube ich, könnte meines Erachtens mit Teil des Problems sein, dass du als Spitalsarzt die Kasse, ich unterstelle das, was dann mal wenig Interesse hat, dass diese Leistungen, die quasi auch im Spital erbracht werden, könnte rein theoretisch um den Patienten zu verbessern."

*‘In Austria, the hospital system and the private practice system are separate, they are two different pots, and at the moment, and I think this could be part of the problem, as a hospital doctor, the health insurance fund, I assume, has little interest in the fact that these services, which are also provided in hospitals, could theoretically improve the patient's condition.’*

Code: ● Wirtschaftliche Faktoren Land / Bund / Kasse	Weight score: 0

C1 200224, 466 - 471

Created: 01.05.2024 21:30 by jmlut, Modified: 01.05.2024 21:30 by jmlut

Area: 423	0.96%

33.

"das hat glaube ich schon ein bisschen was damit zu tun eben, dass so in den Köpfen, wenn das Spital das will, das soll der Spital das auch bezahlen, sozusagen und sich darum kümmern und diese Vernetzung glaube ich ist in England wahrscheinlich, vermutlich besser, weil das
keine getrennten Töpfe sind, sondern wenn es halt das staatliche Gesundheitssystem ist, wo heute der niedergelassene Bereich und der Spitalsbereich ein Dach hat und bei uns geht das Match immer so hin und her, wer übernimmt das jetzt und wenn ich jetzt sage ich hätte gern Physiotherapie eben für den Patienten, dass der fit wird, dann sagt der Spital ja immer in dem Moment, wo er nicht im Spital ist, muss das der Niedergelassene bei uns machen und der Niedergelassene, vermute ich jetzt, das ist vielleicht eben, das warum es nicht so gut funktioniert, sagt dann halt, na ja, ich meine, ich übernehme jetzt nicht die Kosten für eine Physiotherapie, damit der dann im Spital fitter ist, also da soll doch das Spital schauen, dass die Strukturen passen, das ist jetzt so salopp, vielleicht eine Erklärungen dafür, aber diese Schnittstelle ist einfach furchtbar mühsam."

*"I think that has something to do with the fact that people think that if the hospital wants it, then the hospital should pay for it, so to speak, and take care of it, and I think this kind of networking is probably better in England because*

*there are no separate funds, but rather it's the state health system, where today the private practice sector and the hospital sector are under one roof, whereas here the match always goes back and forth, who is responsible for it now, and if I say I would like physiotherapy for the patient so that he gets fit, then the hospital always says that if he is not in the hospital, the private practice has to do it, and the private practice, I suppose, that's maybe why it doesn't work so well, then just says, well, I mean, I'm not going to cover the costs of physiotherapy so that he can get fitter in hospital, so the hospital should make sure that the structures are right. That's a bit blunt, perhaps, but it explains why this interface is so terribly tedious."*

Code: ● Wirtschaftliche Faktoren Land / Bund / Kasse	Weight score: 0

C1 200224, 489 - 504

Created: 01.05.2024 21:32 by jmlut, Modified: 01.05.2024 21:32 by jmlut

Area: 1157	2.63%

34.

"Und deswegen habe ich jetzt nicht das Gefühl, ohne es jemals ausprobiert zu haben, dass wenn ich jetzt zum Patienten sage und ihm einen Brief mitgebe, er sollte jetzt bis zur Operation, diese und jene Maßnahmen ergreifen, gehen Sie bitte zum Hausarzt, der soll sich darum kümmern. Und vielleicht hat er dann einen super Hausarzt, und ich weiß aber ehrlich
gesagt nicht, ob der die Möglichkeit hat, es überhaupt umzusetzen, ob das irgendjemand bezahlt."

*"And that's why I don't feel, without ever having tried it, that if I now tell the patient and give him a letter saying that he should take these and those measures until the operation, please go to your family doctor, who will take care of it. And maybe he has a great GP, but I honestly*

*don't know if he has the means to implement it at all, if anyone will pay for it."*

Code: ● Wirtschaftliche Faktoren Land / Bund / Kasse	Weight score: 0

C1 200224, 526 - 533

Created: 01.05.2024 21:33 by jmlut, Modified: 01.05.2024 21:33 by jmlut

Area: 475	1.08%

35.

"Also, ob der dann sagen muss, naja, die Kasse vergütet das gar nicht. Also, ich kann sie jetzt nicht zu einer Diätologin schicken, zum Physiotherapeuten schicken, dass die sie da präoperativ optimieren, weil ich wüsste es gar nicht, was ich da hinschreiben soll, als Diagnose, damit sie das bezahlt bekommen."

‘*So, whether he then has to say, well, the health insurance fund doesn't cover that at all. So, I can't send her to a dietitian or a physiotherapist to optimise her preoperatively, because I wouldn't know what to write as a diagnosis to get it paid for.’*

Code: ● Wirtschaftliche Faktoren Land / Bund / Kasse	Weight score: 0

C1 200224, 535 - 538

Created: 01.05.2024 21:34 by jmlut, Modified: 01.05.2024 21:34 by jmlut

Area: 311	0.71%

36.

"Vielleicht geht das, da kenne ich mich zu wenig aus, muss ich ganz ehrlich sagen. Aber das wäre natürlich eigentlich das Wünschenswerte. Weil hier im Spital funktioniert es offensichtlich nicht, weil die spielen den Ball immer weiter, wir sind dafür nicht zuständig.
Aber ob die Möglichkeit überhaupt, ob das abgebildet ist im System, das kann ich dir ehrlich gesagt gar nicht sagen. Also, das..."

*"Maybe that's possible, but I have to be honest and say that I don't know enough about it. But that would of course be desirable. Because here in the hospital it obviously doesn't work, because they keep passing the buck and say it's not their responsibility.*

*But whether that's even possible, whether it's reflected in the system, I honestly can't say. So, that..."*

Code: ● Wirtschaftliche Faktoren Land / Bund / Kasse	Weight score: 0

C1 200224, 540 - 546

Created: 01.05.2024 21:34 by jmlut, Modified: 01.05.2024 21:34 by jmlut

Area: 418	0.95%

37.

"Ideal ist es nicht, nein, und ja, ich glaube, da müsste man vielleicht einmal, wenn man das wirklich einführen müsste, dann ist eine Abteilung zu wenig, ein Spital zu wenig eben, sondern dann müsste man schon geschlossen sagen, Leute, ihr müsst das System umstellen."

*‘It's not ideal, no, and yes, I think that if you really had to introduce it, then one department would not be enough, one hospital would not be enough, but you would have to say to people, “Look, you have to change the system.”’*

Code: ● Wirtschaftliche Faktoren Land / Bund / Kasse	Weight score: 0

C1 200224, 579 - 582

Created: 01.05.2024 21:36 by jmlut, Modified: 01.05.2024 21:36 by jmlut

Area: 269	0.61%

38.

"Das Land und man müsste sagen wir machen ein Programm, sozusagen für unsere bevölkerung, wo wir das einführen. Und wo uns das bewusst ist, dass das zu einer Verbesserung führt. Aber da wäre der Träger oder die Politik dann gefragt, sage ich jetzt einmal so."

*‘The state and, one might say, we are creating a programme, so to speak, for our population, where we are introducing this. And where we are aware that this will lead to an improvement. But then the funding agency or politicians would be called upon, I would say.’*

Code: ● Wirtschaftliche Faktoren Land / Bund / Kasse	Weight score: 0

C1 200224, 586 - 589

Created: 01.05.2024 21:37 by jmlut, Modified: 01.05.2024 21:37 by jmlut

Area: 260	0.59%

39.

"Und ob das jetzt daran liegt, dass wirunterschiedliche Finanzierungssysteme haben zwischen stationären Bereich und niedergelassenen Bereich. Und woran das liegt, ob das einfach jetzt da in unseren Köpfen ist, Stichwort Kur und Reha..."

*"And whether that's because we have different financing systems for inpatient and outpatient care. And why that is, whether it's just in our heads, keyword spa and rehabilitation..."*

Code: ● Wirtschaftliche Faktoren Land / Bund / Kasse	Weight score: 0

C1 200224, 728 - 731

Created: 01.05.2024 21:43 by jmlut, Modified: 01.05.2024 21:43 by jmlut

Area: 237	0.54%

40.

"Da werden wir jetzt, glaube ich, eine Kosten -Nutzen -Rechnung brauchen. Ob man das so finanzieren kann, ob es vor allem auch zum Beispiel Förderung vom Land gibt, wie es es für das ERAS tatsächlich gibt, also da gibt es ja eine Förderung, aber die wird man sich, glaube ich, in der Größenordnung anschauen müssen, inwiefern es die Personal- und Zusatzkosten decken kann."

*"I think we'll need to do a cost-benefit analysis now. Whether it can be financed in this way, whether there is funding from the state, for example, as there is for ERAS, where there is funding, but I think we will have to look at the scale of this funding to see to what extent it can cover the personnel and additional costs."*

Code: ● Wirtschaftliche Faktoren Land / Bund / Kasse	Weight score: 0

C2 180324, 230 - 234

Created: 20.05.2024 21:22 by Jan, Modified: 20.05.2024 21:22 by Jan

Area: 375	1.10%

41.

"Und dann, glaube ich, wird es auch ganz relevant sein, wie das bezahlt wird und wer das bezahlt. Weil dann je extramuraler das Ganze wird, desto weniger kann es quasi eine Umwegsrentabilität erlangen über eine kürzere Liegedauer oder wenige Komplikationen. Desto mehr wird derjenige der die Leistung erbringt, fragen, was er dafür bekommt und dann glaube ich, wird man sich da auch Finanzierungsmodelle überlegen müssen."

*" And then, I think it will also be very relevant how it is paid for and who pays for it. Because the more extramural the whole thing becomes, the less it can achieve indirect profitability through shorter hospital stays or fewer complications. The more the person providing the service will ask what they will get in return, and then I think financing models will also have to be considered."*

Code: ● Wirtschaftliche Faktoren Land / Bund / Kasse	Weight score: 0

C2 180324, 323 - 328

Created: 20.05.2024 21:25 by Jan, Modified: 20.05.2024 21:25 by Jan

Area: 425	1.25%

42.

"ohne dass es da eine spezielle Art der Abrechnung gibt, ist es wahrscheinlich gar nicht so einfach umzusetzen, dass man sagt, okay gut, sie haben jetzt einen Operationstermin in fünf Wochen, wir brauchen innerhalb der nächsten 10 Tage einen Prähabilitationstermin für Sie,
das Sie da instruiert werden."

*"Without a specific billing method, it is probably not that easy to implement, to say, okay, you now have a surgery date in five weeks, we need a prehabilitation appointment for you within the next 10 days, so that you can be instructed."*

Code: ● Wirtschaftliche Faktoren Land / Bund / Kasse	Weight score: 0

C2 180324, 338 - 342

Created: 20.05.2024 21:25 by Jan, Modified: 20.05.2024 21:25 by Jan

Area: 306	0.90%

43.

"Ich glaube, dass es wahrscheinlich über einen entsprechenden Bonus leichter machbar wäre, wenn man die Leute darauf aufmerksam macht, dass es für den Patienten besser ist und dass es aber entsprechend mit einer finanziellen Unterstützung kommt, dann werden die Leute leichter darauf aufmerksam. In dem Moment, wo das eine eigene Initiative ist, und das ist es in Österreich de-facto noch, bedarf es sehr viel mehr einer persönlichen Begeisterung für das Projekt.
Je besser man es vielleicht zentral verankert, dass man sagt, okay, man bietet für die Erstellung einer Prähabilitationsambulanz als solches einen finanziellen Bonus an, an den Krankenhäusern, glaube ich, dann könnte das eine Motivation sein.
Und im Umkehrschluss, vielleicht ist es einfach die fehlende Finanzierung, Schrägstrich, das fehlende Anlocken quasi solcher Strukturen. Das was möglicherweise dazu führt, dass es einfach nicht so sehr umgesetzt wird, könnt ich mir vorstellen."

*"I believe that it would probably be easier to achieve this with a corresponding bonus, if people were made aware that it is better for the patient and that it comes with financial support, then people would be more receptive to it. When it is an individual initiative, which is still the case in Austria, it requires much more personal enthusiasm for the project.*

*The better it is anchored centrally, for example by offering a financial bonus to hospitals for setting up a prehabilitation outpatient clinic, the more motivating it could be.*

*And conversely, perhaps it is simply the lack of funding, slash, the lack of attraction of such structures. I could imagine that this is what might be leading to it simply not being implemented to such an extent."*

Code: ● Wirtschaftliche Faktoren Land / Bund / Kasse	Weight score: 0

C2 180324, 458 - 475

Created: 20.05.2024 21:36 by Jan, Modified: 20.05.2024 21:36 by Jan

Area: 1014	2.98%

4.2. Wirtschaftliche Faktoren KH

1.

"Oder, dass vielleicht ein Krankenanstaltenträger, ein größerer, sagt, wir machen so eine Studie, weil wir uns daraus erhoffen, dass eben die Liegedauer abnimmt und die Patienten
schneller wieder fit sind, dass wir die wieder auf die Straße rausschicken können."

*"Or perhaps a larger hospital operator says, we are conducting a study because we hope that it will reduce the length of stay and patients will recover more quickly, so that we can send them back out onto the streets."*

Code: ● Wirtschaftliche Faktoren KH	Weight score: 0

S1 080324, 155 - 159

Created: 20.05.2024 17:01 by Jan, Modified: 20.05.2024 17:01 by Jan

Area: 264	1.01%

2.

"nachdem man nicht nach Tagsatz mehr bezahlt wird, sondern nach... so ein LKF-Modell stellt ja grob gesprochen auf die DRGs ab, da müssen eigentlich die Krankenanstalten-Träger darauf bedacht sein, dass die Patienten schnell wieder rauskommen."

*‘Now that you are no longer paid by the day rate, but by... such an LKF model is roughly based on DRGs, so hospital operators actually have to make sure that patients are discharged quickly.’*

Code: ● Wirtschaftliche Faktoren KH	Weight score: 0

S1 080324, 166 - 169

Created: 20.05.2024 17:02 by Jan, Modified: 20.05.2024 17:02 by Jan

Area: 245	0.94%

3.

"das Payment System wäre schon so, dass der Träger von einer kürzeren Liegedauer profitieren würde."

*‘The payment system would be such that the operator would benefit from a shorter length of stay.’*

Code: ● Wirtschaftliche Faktoren KH	Weight score: 0

S1 080324, 174 - 175

Created: 20.05.2024 17:02 by Jan, Modified: 20.05.2024 17:02 by Jan

Area: 99	0.38%

4.

"Das gibt es ja im Spitalsetting auch, wobei das natürlich jetzt nicht so patientenfreundlich und auch nicht im Sinne des Kostenträgers ist, wahrscheinlich, dass die Patienten schon vor der OP sich dort heimisch einrichten."

*‘This also happens in hospitals, although it is of course not very patient-friendly and also not in the interests of the cost bearer, as patients are likely to settle in there before the operation.’*

Code: ● Wirtschaftliche Faktoren KH	Weight score: 0

S1 080324, 223 - 226

Created: 20.05.2024 17:10 by Jan, Modified: 20.05.2024 17:10 by Jan

Area: 225	0.86%

5.

"Das wäre eine Möglichkeit, dass man vielleicht eine Ambulanz einrichtet, unten
eine Prähabilitationsambulanz,"

*‘One possibility would be to set up an outpatient clinic, a prehabilitation outpatient clinic downstairs.’*

Code: ● Wirtschaftliche Faktoren KH	Weight score: 0

P1 010324, 234 - 235

Created: 20.05.2024 18:47 by Jan, Modified: 20.05.2024 18:47 by Jan

Area: 110	0.29%

6.

"Vielleicht Überforderung der personellen Kapazitäten, dass wir die nicht haben, dass vielleicht die [entfernt] sagt: "sorry, dafür haben wir kein Geld, kein Personal."

*‘Perhaps overburdening of personnel capacities, that we don't have them, that perhaps the [removed] says: “sorry, we don't have the money or the personnel for that.”’*

Code: ● Wirtschaftliche Faktoren KH	Weight score: 0

P1 010324, 244 - 246

Created: 20.05.2024 18:47 by Jan, Modified: 20.05.2024 18:47 by Jan

Area: 169	0.44%

7.

"Also meine Wahrnehmung ist wohl die, dass es derzeit, sozusagen, nachdem es etwas Neues ist und etwas Neues immer gewissen Hürden unterworfen ist, noch zu wenig Strukturen gibt für die Mitarbeiter, die derzeit sozusagen arbeiten, auch noch nicht zusätzlich Ressourcen geschaffen wurden, um diese neue Aufgabe zusätzlich auch noch zu übernehmen.
Man braucht dann auch noch Platz, natürlich, wo man das macht. Also diese Strukturen müssen erst einmal definiert werden. Ich spreche gar noch nicht davon, dass das jetzt eine Leistung ist, die auch abgegolten wird."

*"So my perception is that, at the moment, since it's something new and new things always face certain hurdles, there are still too few structures in place for the employees who are currently working, and no additional resources have been created to take on this new task.*

*Of course, you also need space to do this. So these structures need to be defined first. I'm not even talking about the fact that this is now a service that is also remunerated."*

Code: ● Wirtschaftliche Faktoren KH	Weight score: 0

A1 210224, 132 - 140

Created: 09.05.2024 17:14 by jmlut, Modified: 09.05.2024 17:14 by jmlut

Area: 584	1.47%

8.

"Abgerechnet wird eigentlich noch gar nichts. Also es läuft jetzt mal als Pilot, wo man mal sozusagen das Ganze auch dann wissenschaftlich natürlich begleitet."

*‘Nothing is actually being billed yet. So it's running as a pilot project at the moment, where the whole thing is being scientifically monitored, so to speak.’*

Code: ● Wirtschaftliche Faktoren KH	Weight score: 0

A1 210224, 162 - 163

Created: 09.05.2024 17:16 by jmlut, Modified: 09.05.2024 17:16 by jmlut

Area: 159	0.40%

9.

"Würde man das jetzt z. B. auf viele Patienten ausdehnen, wo es vielleicht Sinn machen könnte aus derzeitiger Sicht, dann würde das sicher derzeit über den Pilot hinaus unsere Ressourcen, überdehnen - das könnten wir gar nicht bringen."

*‘If we were to extend this to many patients, for example, where it might make sense from the current perspective, then that would certainly overstretch our resources beyond the pilot project – we couldn't manage that.’*

Code: ● Wirtschaftliche Faktoren KH	Weight score: 0

A1 210224, 180 - 182

Created: 09.05.2024 17:18 by jmlut, Modified: 09.05.2024 17:18 by jmlut

Area: 236	0.59%

10.

"Aus eigenen Reserven, mit den Reserven wie vor der Prähabilitation. Insofern, wir sprechen hier jetzt einmal von ungefähr 100 Patienten, die da eingeschlossen werden, über einen Zeitraum von über einen Jahr. Das heißt, es sind jetzt nicht so viele Patienten.
Wenn man aber mehr einschließen würde, sozusagen alle großen Operationen, dann wären wir deutlich über 1000. Das wäre mit den derzeitigen Strukturen gar nicht schaffbar."

*"From our own reserves, with the reserves as before prehabilitation. In this respect, we are now talking about approximately 100 patients who are included over a period of more than a year. That means there are not that many patients at the moment.*

*However, if we were to include more, i.e. all major operations, then we would be well over 1000. That would not be feasible with the current structures."*

Code: ● Wirtschaftliche Faktoren KH	Weight score: 0

A1 210224, 186 - 193

Created: 09.05.2024 17:19 by jmlut, Modified: 09.05.2024 17:19 by jmlut

Area: 451	1.14%

11.

"Und man muss ja auch sagen, es muss ja dann auch zum Haus dazu passen. Es muss ja auch lokal umsetzbar sein."

*‘And you have to say, it has to fit in with the house. It has to be locally feasible.’*

Code: ● Wirtschaftliche Faktoren KH	Weight score: 0

A1 210224, 205 - 206

Created: 09.05.2024 17:21 by jmlut, Modified: 09.05.2024 17:21 by jmlut

Area: 109	0.27%

12.

"Dann ein ganz wichtiger Punkt, auch der Arbeitsplatz, also wirklich, welche Ambulanzen können dafür freigestellt sein. Bei uns sind die Ambulanzen so dicht getacktet in der Regel, dass es in der Regel gar keinen freien Raum gibt, wo man noch zusätzlich etwas machen könnte."

*‘Then there is a very important point, namely the workplace, i.e. which ambulances can actually be made available for this purpose. In our case, the ambulances are usually so tightly scheduled that there is generally no free space where anything additional could be done.’*

Code: ● Wirtschaftliche Faktoren KH	Weight score: 0

A1 210224, 257 - 260

Created: 09.05.2024 17:24 by jmlut, Modified: 09.05.2024 17:24 by jmlut

Area: 276	0.69%

13.

"Also, dass es derzeit für viele Patienten umsetzbar ist, ist derzeit sicher sehr schwierig."

*‘So, it is certainly very difficult to implement for many patients at the moment.’*

Code: ● Wirtschaftliche Faktoren KH	Weight score: 0

A1 210224, 320 - 321

Created: 09.05.2024 17:27 by jmlut, Modified: 09.05.2024 17:27 by jmlut

Area: 92	0.23%

14.

"Da ist es tatsächlich so, dass man es einfach dann ausdehnt auf gewisse Gruppen, die am meisten davon profitieren, einfach ausdehnt. Also ich könnte mir vorstellen, dass wir es dann auf andere Patienten ausdehnt, also wir sprechen jetzt mal von den kolorektalen Patienten, dass man es vielleicht ausdehnt auf andere Gruppen, die auch davon profitieren könnten, könnten die Prostata-Patienten sein, zum Beispiel, von denen wir sehr viele haben, also
Prostatektomie-Patienten.
Das wäre eine Gruppe oder andere Patienten, die davon wirklich profitieren, wo man auch eine größere Zahl hat. Das gehört natürlich dann auch wissenschaftlich betreut, dass man sozusagen die Hälfte mit, die Hälfte ohne hat oder eine historische Kontrollgruppe hat, was weniger gut ist.
Und insofern kann man dann sozusagen mit den gewonnenen Zahlen besser argumentieren und dann auch Mitarbeiterressourcen rekrutieren, weil sozusagen von einer kleinen Gruppe in einen Piloten anschauen oder therapieren bis hin zu jetzt machen wir aber alle die Frage kommen.
So ein großer Sprung geht sich nicht aus, da muss man realistisch bleiben..."

*"It is actually the case that you simply extend it to certain groups who benefit most from it, simply extend it. So I could imagine that we would then extend it to other patients, i.e. we are now talking about colorectal patients, that you might extend it to other groups who could also benefit from it, such as prostate patients, for example, of whom we have a great many, i.e.*

*prostatectomy patients.*

*That would be one group, or other patients who would really benefit from it, where there are also larger numbers. Of course, this would then also have to be scientifically monitored, so that you have half with and half without, or a historical control group, which is less good.*

*And in this respect, you can then argue better with the figures obtained and also recruit staff resources, because we are looking at a small group in a pilot project or treating them, but now we are all asking the question.*

*Such a big leap is not possible, you have to remain realistic..."*

Code: ● Wirtschaftliche Faktoren KH	Weight score: 0

A1 210224, 325 - 343

Created: 09.05.2024 17:27 by jmlut, Modified: 09.05.2024 17:28 by jmlut

Area: 1176	2.96%

15.

"Unsere Erfahrungen mit den Niedergelassenen sind grundsätzlich sehr gut. Das funktioniert grundsätzlich wirklich sehr gut. So weit sind wir aber noch nicht gekommen. Es ist schon aber so, dass aus derzeitiger Sicht der Aufwand, der finanzielle Aufwand pro Patient, wenn er jetzt praktisch prähospital ist, wenn jetzt prähospital ist, deutlich geringer ist."

*‘Our experience with private practitioners has been very good overall. It works really well in principle. But we haven't gotten that far yet. However, from the current perspective, the financial expenditure per patient is significantly lower when they are in the pre-hospital phase, when they are in the pre-hospital phase.’*

Code: ● Wirtschaftliche Faktoren KH	Weight score: 0

A1 210224, 382 - 386

Created: 09.05.2024 17:31 by jmlut, Modified: 09.05.2024 17:31 by jmlut

Area: 360	0.91%

16.

"Nein. Was einfach in den letzten Jahren so stark in den Vordergrund getreten ist, dass man diese Dinge unter wirtschaftlichen Aspekten betrachtet und diese sehr, sehr in den Vordergrund gestellt hat. Wenn man so eine Person lukrieren kann, dann wahrscheinlich nur auf dem Weg um zu erläutern, dass eine präoperative Optimierung letztendlich einen wirtschaftlicher Vorteil bringt, in dem die Patienten postoperativ weniger aufwendig sind, komplikationsbehaftet sind, das Haus schneller verlassen.
Und was jetzt auch noch dazu kommen wird und in den nächsten Jahren verstärkt, ist Personalmangel und zwar in jeder Hinsicht, wo auch Menschen abgehen, gerade in der Pflege, die sehr nah am Patienten sind und da viel machen können, aber auch bei Ärzten und Physiotherapeuten.
Also wir haben - wir schlittern auf einen großen Personalmangel zu."

*"No. What has become so prominent in recent years is that these things are viewed from an economic perspective and have been given very, very high priority. If you can attract such a person, then probably only by explaining that preoperative optimisation ultimately brings an economic advantage in that patients require less post-operative care, have fewer complications and leave the hospital more quickly.*

*And what will now be added to this and intensify in the coming years is a shortage of personnel in every respect, with people leaving, especially in nursing, where they are very close to the patients and can do a lot, but also among doctors and physiotherapists.*

*So we are sliding towards a major shortage of personnel."*

Code: ● Wirtschaftliche Faktoren KH	Weight score: 0

A2 220422, 198 - 211

Created: 09.05.2024 19:40 by jmlut, Modified: 09.05.2024 19:41 by jmlut

Area: 884	2.48%

17.

"Nein, ich glaube, das kommt ungefähr hin, weil das ist das, was mir aufgefallen ist, die Wirtschaftlichkeit enorm in den Vordergrund getreten ist, für meinen Begriff zu Lasten des Wohles des Patienten."

*‘No, I think that's about right, because that's what I've noticed: economic efficiency has become extremely important, in my opinion at the expense of patient welfare.’*

Code: ● Wirtschaftliche Faktoren KH	Weight score: 0

A2 220422, 220 - 222

Created: 09.05.2024 19:41 by jmlut, Modified: 09.05.2024 19:41 by jmlut

Area: 203	0.57%

18.

"Und wenn man so ein Projekt flächendeckend durchziehen will, wird man irgendwo belegen müssen, dass das wirtschaftlich interessant ist."

*‘And if you want to implement such a project across the board, you will have to prove somewhere that it is economically viable.’*

Code: ● Wirtschaftliche Faktoren KH	Weight score: 0

A2 220422, 224 - 225

Created: 09.05.2024 19:41 by jmlut, Modified: 09.05.2024 19:41 by jmlut

Area: 136	0.38%

19.

"Ich kann natürlich nur das Bundesland beobachten, in dem ich jahrelang tätig war. Sozusagen, je kleiner der Betrieb, um so einfacher Maßnahmen umzusetzen, mit denen alle gut zurechtkommen, um so einfacher zu sagen, das kann uns helfen, auch Kosten zu sparen, das wird uns helfen, Patienten besser zu betreuen.
Je größer die Einheiten werden, um so schwieriger ist es umzusetzen, weil da sehr viele Interessen da mit hinein spielen."

*"Of course, I can only observe the federal state in which I worked for many years. The smaller the business, the easier it is to implement measures that everyone can cope with, the easier it is to say that this can help us save costs and provide better care for patients.*

*The larger the units become, the more difficult it is to implement, because there are so many interests at stake."*

Code: ● Wirtschaftliche Faktoren KH	Weight score: 0

A2 220422, 266 - 272

Created: 09.05.2024 19:43 by jmlut, Modified: 09.05.2024 19:43 by jmlut

Area: 453	1.27%

20.

"Die entscheidenden Faktoren waren wirklich wiederum wirtschaftliche. Wo können wir einsparen, also wo können wir dem Haus Kosten ersparen.
Was können wir in der präoperativen Routine, die bislang geherrscht hat anders machen, besser machen, vernünftiger machen. Aber der große Überbegriff war Kosten sparen."

*"The decisive factors were really economic again. Where can we save money, i.e. where can we save the hospital costs?*

*What can we do differently, better, more sensibly in the preoperative routine that has prevailed up to now? But the big overarching term was cost saving."*

Code: ● Wirtschaftliche Faktoren KH	Weight score: 0

A2 220422, 499 - 504

Created: 09.05.2024 19:48 by jmlut, Modified: 09.05.2024 19:49 by jmlut

Area: 330	0.93%

21.

"Absolut, Geld und Personalentlastung, also nicht Entlassung, sondern Entlastung des Personals, was ja letztendlich auch wieder mit Geld verbunden ist."

*‘Absolutely, money and staff relief, i.e. not redundancies, but relief for staff, which ultimately also involves money.’*

Code: ● Wirtschaftliche Faktoren KH	Weight score: 0

A2 220422, 539 - 540

Created: 09.05.2024 19:50 by jmlut, Modified: 09.05.2024 19:50 by jmlut

Area: 151	0.42%

22.

"Man kann Ressourcen generieren und die vorhandenen kann man vielleicht auch schonender damit behandeln, oder ja, weil sie... je weniger Komplikationen, umso besser operiert der Patient postoperativ ist, umso angenehmer, umso leichter ist er auch zu pflegen und zu handlen. Nicht immer, aber sehr oft, es geht letztendlich immer nur um die Kosten."

*"You can generate resources and perhaps treat the existing ones more gently, or yes, because they... the fewer complications, the better the patient's postoperative condition, the more comfortable, the easier they are to care for and handle.*

*Not always, but very often, it ultimately always comes down to the costs."*

Code: ● Wirtschaftliche Faktoren KH	Weight score: 0

A2 220422, 544 - 549

Created: 09.05.2024 19:51 by jmlut, Modified: 09.05.2024 19:51 by jmlut

Area: 366	1.03%

23.

"Ja, natürlich geht es ums Geld und es geht tatsächlich ja darum, dass man um neue Strukturen oder neue Projekte zu implementieren, motivierte und engagierte Mitarbeiter braucht. Und es ist sehr einfach, in der derzeitigen Situation ist es einfach so angespannt, dass die Kolleginnen und Kollegen nicht mehr so bereit sind, von ihrer Freizeit sehr viel in die Arbeit zu investieren, sagen wir es einmal ganz vorsichtig.
Nicht weil sie unendlich faul sind, sondern weil da irgendetwas fehlt an Anreiz."

*"Yes, of course it's about money, and it's actually about the fact that you need motivated and committed employees to implement new structures or new projects. And it's very easy, in the current situation it's just so tense that colleagues are no longer so willing to invest a lot of their free time in work, let's put it very cautiously.*

*Not because they are infinitely lazy, but because there is something missing in terms of incentive."*

Code: ● Wirtschaftliche Faktoren KH	Weight score: 0

A2 220422, 571 - 578

Created: 09.05.2024 19:52 by jmlut, Modified: 09.05.2024 19:52 by jmlut

Area: 522	1.46%

24.

"Da sind wir weit weg davon, dass die da irgendwie trainieren oder mit ihnen körperlich das verbessern und so weiter. Und auch da auf Nachfragen, warum, wieso geht das nicht: ja dafür haben wir nicht die Ressourcen, dafür sind wir nicht da."

*‘We are a long way from training them or improving their physical condition and so on. And when asked why this is not possible, the answer is: we don't have the resources for that, that's not what we're here for.’*

Code: ● Wirtschaftliche Faktoren KH	Weight score: 0

C1 200224, 185 - 188

Created: 01.05.2024 16:24 by jmlut, Modified: 01.05.2024 16:24 by jmlut

Area: 242	0.55%

25.

"Also ich kenne mich aus im stationären Bereich. Das heißt, ich kann, wenn ich der Meinung bin, dass der Physio und also präoperative Optimierung braucht, dann kann ich nur hier drinnen zuweisen. Und die hier drinnern sagen mir ich hab eigentlich auch nicht die Ressourcen für das."

*Well, I know my way around the inpatient sector. That means that if I think that the patient needs physiotherapy and preoperative optimisation, then I can only refer them here. And the people here tell me that they don't really have the resources for that either.’*

Code: ● Wirtschaftliche Faktoren KH	Weight score: 0

C1 200224, 268 - 271

Created: 01.05.2024 16:33 by jmlut, Modified: 01.05.2024 16:33 by jmlut

Area: 283	0.64%

26.

"Aber dann ist es so, dass das dann genau an den Dingen, die ich vorher schon gesagt habe, meistens scheitert eben. Du nimmst dann Kontakt auf mit all jenen Stellen, die da eigentlich dazugehören würden, und du kriegst dann immer die gleichen Antworten.
Ja, wer soll das machen eben? Ich habe weder die Leute dafür. Wir sind jetzt schon am Anschlag, wir können nicht mehr. Und diese Betreuung können wir hier im stationären Bereich nicht anbieten. Und ambulant machen wir nicht so irgendwie."

*"But then it's usually the things I mentioned earlier that cause it to fail. You contact all the relevant authorities, and you always get the same answers.*

*Yes, who's supposed to do that? I don't have the people for it. We're already at our limit, we can't do any more. And we can't offer this kind of care here in the inpatient sector. And we don't do it on an outpatient basis either."*

Code: ● Wirtschaftliche Faktoren KH	Weight score: 0

C1 200224, 385 - 392

Created: 01.05.2024 16:38 by jmlut, Modified: 01.05.2024 16:38 by jmlut

Area: 513	1.17%

27.

"Und das sind dann halt nur- dann gibt es ein paar zaghafte Schritte, und das funktioniert eine Zeit lang, und dann verläuft das wieder alles. Und ich meine, jetzt haben wir schon vor, sag ich jetzt einmal so, also das Projekt läuft, also wir wollen ERAS einführen auf der Chirurgie da, und nur wie du richtig sagst, wenn man sich das in Österreich im Umfeld anschaut, dann gibt es ein Nachbarspital eben, das hat das jetzt seit einem halben Jahr, Jahr - ERAS-Nurse,
aber das ist das erste ERAS-Zentrum in ganz Österreich, sonst gibt es da Nada, und das ist eigentlich, sag ich jetzt einmal recht traurig, aber die - woran das liegt, keine Ahnung, aber wahrscheinlich meistens eben daran, dass man halt nichts hat eben, wie man das abrechnen kann."

*“And then there are just a few tentative steps, and it works for a while, and then it all fizzles out again. And I mean, now we already have – let’s say – the project is running, we want to implement ERAS in surgery here, and just like you said, when you look at the environment in Austria, there’s a neighboring hospital that’s had it for half a year or a year now – an ERAS nurse – but that’s the first ERAS center in all of Austria. Otherwise, there’s nothing. And honestly, I’d say that’s quite sad. Why that is, I have no idea, but probably mostly because there’s no way to bill for it.”*

Code: ● Wirtschaftliche Faktoren KH	Weight score: 0

C1 200224, 555 - 564

Created: 01.05.2024 21:35 by jmlut, Modified: 01.05.2024 21:35 by jmlut

Area: 755	1.72%

28.

"Bei uns am Haus hat es de facto noch keine Rolle gespielt, weil wir einfach bisher das Personal dafür nicht gehabt haben"

*“At our institution, it hasn’t played any role so far, simply because we haven’t had the personnel for it until now.”*

Code: ● Wirtschaftliche Faktoren KH	Weight score: 0

C2 180324, 108 - 109

Created: 20.05.2024 21:17 by Jan, Modified: 20.05.2024 21:17 by Jan

Area: 121	0.36%

29.

"Ich glaube nicht, dass es was ist, was man jedem Patienten anbieten kann, weil das über die Kapazitäten geht, und zwar bei Weitem, die wir momentan für solche Sachen zur Verfügung haben.
Wenn man es wirklich ernsthaft betreibt, dann glaube ich, wird man es wahrscheinlich beschränken müssen, entweder auf Patienten, die einfach nicht so fit sind und dafür Potenzial haben oder auf größere Eingriffe. Auch da, glaube ich, wird man selektionieren müssen.
Sonst dass es kapazitätstechnisch einfach extrem viele Arbeitsstunden braucht und die glaube ich kann man momentan so mit unserer Personaldecke wahrscheinlich nicht umsetzen."

*“I don’t think it’s something you can offer to every patient because it exceeds – by far – the capacities we currently have for such things.If it’s taken seriously, I think it will probably have to be limited – either to patients who are not that fit and could benefit, or to major surgeries. Even then, I think some selection will be necessary.*

*Otherwise, it just requires an extreme number of work hours from a capacity standpoint, and I don’t think we can currently implement that with our existing staffing levels.”*

Code: ● Wirtschaftliche Faktoren KH	Weight score: 0

C2 180324, 163 - 174

Created: 20.05.2024 21:19 by Jan, Modified: 20.05.2024 21:19 by Jan

Area: 671	1.97%

30.

"Ich glaube, dass es wahrscheinlich über einen entsprechenden Bonus leichter machbar wäre, wenn man die Leute darauf aufmerksam macht, dass es für den Patienten besser ist und dass es aber entsprechend mit einer finanziellen Unterstützung kommt, dann werden die Leute leichter darauf aufmerksam.
In dem Moment, wo das eine eigene Initiative ist, und das ist es in Österreich de-facto noch, bedarf es sehr viel mehr einer persönlichen Begeisterung für das Projekt.
Je besser man es vielleicht zentral verankert, dass man sagt, okay, man bietet für die Erstellung einer Prähabilitationsambulanz als solches einen finanziellen Bonus an, an den Krankenhäusern, glaube ich, dann könnte das eine Motivation sein.
Und im Umkehrschluss, vielleicht ist es einfach die fehlende Finanzierung, Schrägstrich, das fehlende Anlocken quasi solcher Strukturen. Das was möglicherweise dazu führt, dass es einfach nicht so sehr umgesetzt wird, könnt ich mir vorstellen."

*“I think it would probably be more feasible with an appropriate bonus – if people are made aware that it benefits the patient and that there’s also financial support, then people will pay more attention to it.*

*At the moment, it’s still basically a personal initiative in Austria, and that means it takes a lot more personal enthusiasm for the project.*

*If it were better centrally anchored, like saying: ‘Okay, we offer a financial bonus for establishing a prehabilitation clinic,’ then I think that could be a motivation.*

*And conversely, maybe it’s simply the lack of funding or lack of incentives for these kinds of structures that’s the reason why it hasn’t really been implemented. That’s something I could imagine.”*

Code: ● Wirtschaftliche Faktoren KH	Weight score: 0

C2 180324, 458 - 475

Created: 20.05.2024 21:36 by Jan, Modified: 20.05.2024 21:36 by Jan

Area: 1014	2.98%

31.

"Ich glaube, dass es Unterschiede geben wird zwischen zentralen Krankenhäusern und zwischen sehr peripheren.
Da wird sicherlich ein deutlicher Unterschied da sein. Und auch hinsichtlich der Personaldecke, glaube ich, dass es wahrscheinlich ein Unterschied macht. Je mehr
Personal da ist, desto eher, glaube ich, kann man sich auch mit Dingen beschäftigen, die über die pure Patientenversorgung hinausgehen und dazu gehört ja die Prähabilitation im Wesentlichen."

*“I think there will be differences between central hospitals and more peripheral ones.*

*There will definitely be a noticeable difference. And regarding staffing, I also think it makes a difference. The more staff there is, the more likely it is you can deal with things that go beyond pure patient care – and prehabilitation essentially falls into that category.”*

Code: ● Wirtschaftliche Faktoren KH	Weight score: 0

C2 180324, 482 - 490

Created: 20.05.2024 21:39 by Jan, Modified: 20.05.2024 21:39 by Jan

Area: 484	1.42%

32.

"Das ist zwar ein super Projekt oder ein super Ansatz. Aber wenn das eigentliche Problem ist, dass man die Grundversorgung schon nicht hinkriegt, weil man mit den handelnden Personen quasi nicht zu Rande kommt, weil es zu wenige sind, dann wird es nicht funktionieren, dass man sich mit anderen Dingen beschäftigt.
Das kann vielleicht ein Grundproblem sein, wenn wir ja wissen, dass periphere Häuser immer schwerer besetzbar sind und z .T. einfach das Personal für gewisse Dinge nicht da ist."

*“It’s definitely a great project or a great approach.*

*But if the real problem is that you’re already struggling to provide basic care – because you can’t manage with the people involved, because there are simply too few – then it’s not going to work to focus on other things.*

*That might be a fundamental issue, especially since we know that peripheral hospitals are increasingly hard to staff, and sometimes there just isn’t the personnel for certain tasks.”*

Code: ● Wirtschaftliche Faktoren KH	Weight score: 0

C2 180324, 492 - 499

Created: 20.05.2024 21:39 by Jan, Modified: 20.05.2024 21:39 by Jan

Area: 515	1.51%

33.

"Ich glaube, dass das Wesentliche ist, dass man sich personell und strukturell überlegt, wie das effizient gestaltet werden kann, weil man dann, glaube ich, viele Patienten in das Programm bekommt, wenn man eine zeitlich und räumlich effiziente Lösung basteln kann."

*“I think the key is to carefully consider, in terms of staffing and structure, how it can be organized efficiently – because then, I believe, you can include many patients in the program, if you can create a solution that is efficient in terms of time and space.”*

Code: ● Wirtschaftliche Faktoren KH	Weight score: 0

C2 180324, 551 - 554

Created: 20.05.2024 21:41 by Jan, Modified: 20.05.2024 21:41 by Jan

Area: 267	0.78%

5. Kulturelle Faktoren

5.1. Innovationsklima

1.

"Aber wenn man es in Österreich einsetzen möchte und in die Regelversorgung
implementieren möchte, dann ist es, glaube ich, wirklich ein Weg, der steinig
ist. Solche Standards in die Regelversorgung, in die Fläche zu bekommen, weil sie ja
die Leute überzeugen müssen, dass das eingesetzt wird."

*"But if you want to use it in Austria and implement it in standard care, then I think it really is a rocky road. Getting such standards into standard care, into the area, because you have to convince people that it should be used."*

Code: ● Innovationsklima	Weight score: 0

S1 080324, 287 - 292

Created: 20.05.2024 17:14 by Jan, Modified: 20.05.2024 17:14 by Jan

Area: 314	1.21%

2.

"ich würde jetzt einmal sagen, dass in Österreich die Prävention sowieso total schlecht ist. Da müsste ein Umdenken anfangen"

*"I would say that prevention is totally bad in Austria anyway. There needs to be a rethink"*

Code: ● Innovationsklima	Weight score: 0

PT1 140324, 342 - 343

Created: 20.05.2024 20:09 by Jan, Modified: 20.05.2024 20:09 by Jan

Area: 124	0.35%

3.

"Naja, weil so lange es nicht um Geld geht, ist es uninteressant, glaube ich."

*‘Well, because as long as it's not about money, I don't think it's interesting.’*

Code: ● Innovationsklima	Weight score: 0

PT1 140324, 399 - 399

Created: 20.05.2024 20:10 by Jan, Modified: 20.05.2024 20:10 by Jan

Area: 76	0.21%

4.

"In Österreich ist es so, das haben wir die letzten 150 Jahre gemacht, also
machen wir es die nächsten 150 Jahre auch so."

*"In Österreich ist es so, das haben wir die letzten 150 Jahre gemacht, also machen wir es die nächsten 150 Jahre auch so."*

Code: ● Innovationsklima	Weight score: 0

PT1 140324, 444 - 445

Created: 20.05.2024 20:18 by Jan, Modified: 20.05.2024 20:18 by Jan

Area: 121	0.34%

5.

"mir fällt das halt so auf, weil, ich denke mir, das ist so, ja, wenn man nicht entweder mit wahnsinnigen Kostenersparnissen aufwarten kann, oder eben mit der Not, dann ist das System relativ starr, sagen wir so."

*‘It just strikes me that way because, I think to myself, yes, if you can't come up with either incredible cost savings or just the need, then the system is relatively rigid, let's put it that way.’*

Code: ● Innovationsklima	Weight score: 0

PT1 140324, 529 - 531

Created: 20.05.2024 20:19 by Jan, Modified: 20.05.2024 20:19 by Jan

Area: 213	0.60%

6.

"Deswegen muss das eben von der Ärztekammer oder vom Krankenhaus oder von irgendeiner Institution kommen, die das dann anbietet, weil sonst glaube ich nicht, dass das gut umgesetzt wird."

*‘That's why it has to come from the medical association or the hospital or some institution that then offers it, because otherwise I don't think it will be implemented well.’*

Code: ● Innovationsklima	Weight score: 0

PT1 140324, 535 - 537

Created: 20.05.2024 20:19 by Jan, Modified: 20.05.2024 20:19 by Jan

Area: 187	0.52%

7.

"Wie gesagt, du brauchst irgendein Sprachrohr oder irgendwas, was gewichtig ist, weil sonst, glaube ich, kommst du nicht weit. Also du machst dein eigenes Ding und stellst das selber auf die Beine, als Institution oder Privatperson oder was auch immer.
Und wenn das nachher ein positives Outcome hat und die anderen sehen das, dann kann es schon sein, dass das übernommen wird, aber sonst glaube ich, wenn du keinen Ansprechpartner hast oder jemanden, der das forciert und der Einfluss hat, wie zum Beispiel Ärztekammer sonst irgendwas, dann glaube ich, ist es recht schwierig."

*"As I said, you need some kind of mouthpiece or something that carries weight, because otherwise I don't think you'll get very far. So you do your own thing and set it up yourself, as an institution or a private individual or whatever.*

*And if it has a positive outcome afterwards and the others see it, then it's possible that it will be adopted, but otherwise I think if you don't have a contact person or someone who pushes it and has influence, such as the medical association or something else, then I think it's quite difficult."*

Code: ● Innovationsklima	Weight score: 0

PT1 140324, 604 - 613

Created: 20.05.2024 20:20 by Jan, Modified: 20.05.2024 20:20 by Jan

Area: 601	1.69%

8.

"Der Rahmen gehört geklärt. Der organisatorische finanzielle, administrative Rahmen gehört geklärt. Ein Statement, ja, wir wollen das. Und dann, Bewusstsein oder Einladen und zuweisen."

*"The framework needs to be clarified. The organisational, financial and administrative framework needs to be clarified. A statement, yes, we want this. And then, awareness or invitation and assignment."*

Code: ● Innovationsklima	Weight score: 0

P1 010324, 642 - 644

Created: 20.05.2024 18:58 by Jan, Modified: 20.05.2024 18:58 by Jan

Area: 185	0.48%

9.

"Ja, schon dann Patientenunzufriedenheit und Patientenzufriedenheit, beides. Also Patienten zu aktivieren, dass sie sich da auch dafür engagieren und das auch in Zeitschriften bringen, dass man darüber öffentlich diskutiert, dass man Diskussionsveranstaltungen dazu veranstaltet, dass man in Zeitungen oder jetzt sind sicher die Medien, egal nicht Zeitungen, werden nicht mehr so gelesen, aber Medienlandschaften, ja, eventuell auch im Krankenhaus dieses Wartezimmer-TV, dass man dort auch das einfach schon anbietet und den Patienten zeigt, diese Möglichkeit gibt es, also die Patienten zu informieren, die Niedergelassenen zu
informieren, Krankenhausärzte dafür zu gewinnen, dass das eine gute Sache ist."

*"Yes, patient dissatisfaction and patient satisfaction, both. So activating patients, getting them to get involved in this and getting it into magazines, discussing it in public, organising discussion events about it, publishing it in newspapers or now it's certainly the media, not newspapers, they're not read like that anymore, but media landscapes, yes, possibly also in hospitals, this waiting room TV, simply offering it there and showing patients that this option is available, i.e. informing patients, informing practitioners, getting hospital doctors to believe that this is a good thing."*

Code: ● Innovationsklima	Weight score: 0

K1 280224, 553 - 561

Created: 09.05.2024 20:42 by jmlut, Modified: 09.05.2024 20:42 by jmlut

Area: 713	1.92%

10.

"Also unsere Wahrnehmung auch jetzt in Hinsicht auf dieses PhD -Projekt, wo ich auch involviert bin, ist, dass die Prähabilitation in vielen Bereichen noch nicht läuft, also in vielen Krankenhäusern nicht läuft, auch in vielen operativen Bereichen nicht läuft."

*‘So our perception now with regard to this PhD project, in which I am also involved, is that prehabilitation is not yet running in many areas, i.e. not running in many hospitals, and not running in many surgical areas either.’*

Code: ● Innovationsklima	Weight score: 0

A1 210224, 64 - 67

Created: 09.05.2024 17:10 by jmlut, Modified: 09.05.2024 17:10 by jmlut

Area: 262	0.66%

11.

"Also, glaube ich, es hat ganz viel damit zu tun, dass es aufgeschlossene Menschen braucht, Menschen, die Interesse haben, Menschen, die innovativ sind.
Und das hat sicher mit der Unternehmenskultur zu tun, dass sozusagen unser Krankenhaus jetzt im speziellen Leiter anzieht, also bewusst Leiter sucht, beziehungsweise dann in der Folge, wenn die Leiter erst einmal da sind und die Unternehmenskultur passt, Mitarbeiter anzieht, die motiviert und innovativ sind."

*"Well, I think it has a lot to do with the fact that we need open-minded people, people who are interested, people who are innovative.*

*And it certainly has to do with the corporate culture, so to speak, that our hospital is now specifically attracting managers, i.e. consciously looking for managers, and then, once the managers are in place and the corporate culture is right, attracting employees who are motivated and innovative."*

Code: ● Innovationsklima	Weight score: 0

A1 210224, 215 - 220

Created: 09.05.2024 17:22 by jmlut, Modified: 09.05.2024 17:22 by jmlut

Area: 466	1.17%

12.

"Und nachdem in unserem Haus in anderen Bereichen auch sehr viel Forschung läuft, wie das auch sozusagen nachvollziehbar und auch ganz schlüssig, logisch, dass wir auch in dem Bereich in diesem Hotspot -Prähabilitation - natürlich aufgesprungen sind, weil wir als Haus, wie wir halt sozusagen mit unserer, sozusagen Linie von minimalinvasiver Therapie, verbessertem Outcome, auch eben geringere Wunden, schnelleres Erholen, da natürlich die Prähabilitation schon auch als Möglichkeit sehen, die Patienten schneller wieder in den Alltag zurück zu bekommen."

*‘And since a lot of research is also being carried out in other areas at our hospital, it's understandable and logical that we naturally jumped into this hotspot - prehabilitation - because as a hospital, we see prehabilitation as an opportunity to get patients back into everyday life more quickly with our line of minimally invasive therapy, improved outcome, fewer wounds and faster recovery, so to speak.’*

Code: ● Innovationsklima	Weight score: 0

A1 210224, 222 - 229

Created: 09.05.2024 17:22 by jmlut, Modified: 09.05.2024 17:22 by jmlut

Area: 561	1.41%

13.

"Also was ganz generell ein Thema ist, ist, dass alles was Neu ist einem gewissen Widerstand unterliegt. Das ist einfach der Gedanke hinter dem Change Management. Alles Neue hat einmal eine Bremse, die dem entgegen steht."

*"So what is generally an issue is that everything that is new is subject to a certain amount of resistance. That's simply the idea behind change management. Everything new has a brake that stands in the way."*

Code: ● Innovationsklima	Weight score: 0

A1 210224, 248 - 250

Created: 09.05.2024 17:23 by jmlut, Modified: 09.05.2024 17:23 by jmlut

Area: 222	0.56%

14.

"Es gibt einige Köpfe, also wirklich nur ganz Einzelne, in zum Teil auch leitenden Positionen, die das immer alles sehr schwierig finden. Gerade bei so einem Projekt muss man
natürlich auch wissen, dass nach Möglichkeit diese Köpfe nicht in das Team gezogen werden sollen, weil es sonst nochmal extra schwierig wird."

*"There are some people, really just individuals, sometimes in management positions, who always find it very difficult. With a project like this in particular, you also need to know*

*that these people should not be brought into the team if possible, because otherwise it will be even more difficult."*

Code: ● Innovationsklima	Weight score: 0

A1 210224, 301 - 305

Created: 09.05.2024 17:26 by jmlut, Modified: 09.05.2024 17:26 by jmlut

Area: 319	0.80%

15.

"Dann gibt es natürlich zahlreiche kommunale Träger nach wie vor und Landeshäuser, Organisationen, Träger, also jedes Bundesland hat einen eigenen Krankenhausträger, also im Landesbesitz. Die haben, die sind irgendwie so eine Mischung zwischen ein bisschen Forschung, ein bisschen Innovation, die tun sich zum Teil ganz schwer und da hängt es dann häufig an einzelnen Köpfen in jeweiligen Kliniken, ob da jetzt noch was speziell gepusht wird oder nicht, aber ich glaube, damit hast du schon ein bisschen Eindruck bekommen, dass das eine sehr zerklüftete Krankenhauslandschaft ist, wo man nicht ein Konzept für alle
ausholen kann."

*"Then, of course, there are still numerous municipal providers and state hospitals, organisations, providers, so each federal state has its own hospital provider, i.e. state-owned. They are somehow a mixture of a bit of research, a bit of innovation, some of them are struggling and it often depends on individual heads in the respective hospitals whether something special is pushed or not, but I think that gives you a bit of an impression that this is a very fragmented hospital landscape, where you can't come up with one concept for all."*

Code: ● Innovationsklima	Weight score: 0

A1 210224, 486 - 494

Created: 09.05.2024 17:59 by jmlut, Modified: 09.05.2024 17:59 by jmlut

Area: 636	1.60%

16.

"Und bei den Chirurgien ist es tatsächlich auch so, das sind wirklich auch nur, aber es sind nicht nur Chirurgen, sondern bei den Klinikern in Österreich sind das halt einzige Macher, die das halt für ihren Bereich als sinnvolle erachten und halt umsetzen möchten.
Aber da ist jetzt, da steht keine, wie ich vorhin gesagt habe, keine Gesellschaft oder Struktur dahinter."

*"And in the case of the surgeons, it really is the same, they are really just surgeons, but they are not just surgeons, but among the clinicians in Austria, they are just the only ones who consider this to be useful for their field and want to implement it.*

*But, as I said earlier, there is no organisation or structure behind it."*

Code: ● Innovationsklima	Weight score: 0

A1 210224, 533 - 539

Created: 09.05.2024 18:01 by jmlut, Modified: 09.05.2024 18:01 by jmlut

Area: 391	0.98%

17.

"Auf deinem speziellen Punkt jetzt, wie kann man die Krankenhauskultur verändern? Kann man sagen, es gibt zwei Ansätze, entweder Top -Down oder Bottom -Up. Bottom -Up passiert schon sehr viel, weil sehr viele junge Menschen reindrängern in diese großen Systeme, die Interesse haben, Neues zu machen.
Also da passiert schon wirklich sehr viel. Bottom -Up braucht es natürlich länger, weil die Spitze ist natürlich älter, als die Jungen, die von unten reinkommen. Auch hier habe ich den Eindruck, dass es einige Träger gibt, die wirklich sehr, sehr gut sind."

*"On your specific point now, how can you change the hospital culture? You could say there are two approaches, either top-down or bottom-up. A lot is already happening at the bottom up, because a lot of young people are pushing their way into these large systems who are interested in doing something new.*

*So there really is a lot happening there. Bottom-up takes longer, of course, because the top is naturally older than the young people coming in from below. Here, too, I have the impression that there are some organisations that are really, really good."*

Code: ● Innovationsklima	Weight score: 0

A1 210224, 574 - 582

Created: 09.05.2024 18:02 by jmlut, Modified: 09.05.2024 18:02 by jmlut

Area: 579	1.46%

18.

"Naja, ich glaube, das ist aber jetzt eine persönliche Meinung, die ist wissenschaftlich nicht untermauert. Das ist ein Charakterzug des Österreichers. Wird schon gehen, das ist immer schon so gewesen, warum soll man das jetzt ändern, bis jetzt ist immer gut gegangen, so wie wir da waren."

*"Well, I think that's just a personal opinion, it's not scientifically substantiated. It's an Austrian character trait. It'll be fine, it's always been like that, why should we change it now, it's always been fine the way we've been."*

Code: ● Innovationsklima	Weight score: 0

A2 220422, 291 - 294

Created: 09.05.2024 19:44 by jmlut, Modified: 09.05.2024 19:44 by jmlut

Area: 291	0.82%

19.

"Und eine gewisse Bequemlichkeit, sich aus gewachsenen Strukturen und auch bewährten Strukturen herauszubewegen in irgendwas, wo man nicht genau weiß, was da jetzt rauskommt. Und um Gottes Willen mit mehr Aufwand ist es auch verbunden, wie?"

*"And a certain comfort in moving out of established structures and proven structures into something where you don't know exactly what will come out of it. And for God's sake, it also involves more effort, doesn't it?"*

Code: ● Innovationsklima	Weight score: 0

A2 220422, 296 - 299

Created: 09.05.2024 19:44 by jmlut, Modified: 09.05.2024 19:44 by jmlut

Area: 242	0.68%

20.

"Und dann soll ich da vier Stunden länger bleiben und dann danach? Also ich glaube, das ist ein bisschen kulturell, dass der Österreicher schon dazu neigt, Dinge, die er kennt und die einigermaßen gut funktionieren, so zu lassen. Und ja nicht ändern, bitte schön."

*"And then I'm supposed to stay there four hours longer and then after that? I think it's a bit cultural that Austrians tend to leave things that they know and that work reasonably well as they are. And don't change them, please."*

Code: ● Innovationsklima	Weight score: 0

A2 220422, 301 - 304

Created: 09.05.2024 19:44 by jmlut, Modified: 09.05.2024 19:44 by jmlut

Area: 265	0.74%

21.

"Digitalisierung glaube ich ist sexy und also das funktioniert glaube ich schon"

*‘I think digitalisation is sexy and I think it works,’*

Code: ● Innovationsklima	Weight score: 0

C1 200224, 613 - 613

Created: 01.05.2024 21:37 by jmlut, Modified: 01.05.2024 21:37 by jmlut

Area: 79	0.18%

22.

"es sind so immer die, glaube ich, wenn gewisse Interessen da sind, eben die gut bei der Bevölkerung rüber kommen dann und die Erfolge quasi so präsentierbar sind, dann funktionieren die Umsetzungen ganz gut und ich glaube das, was ich das Gefühl habe, was in diesem Bundesland sehr gut funktioniert, das ist natürlich ein bisschen provokativ, aber es sind bauliche Veränderungen, also es wird extrem gerne gebaut, wir bauen jetzt ein neues Krankenhaus, wir bauen jetzt in [entfernt] einen neuen OP, den kein Mensch braucht, und wir bauen in [entfernt] des Krankenhauses um und wenn man sieht, wir bauen jetzt ein neues Gebäude für dieses und jenes, also diese Dinge sind ja so schöne Erfolge, die man als
Politiker der Bevölkerung immer gut verkaufen kann.
Also in jedem Tal steht ein Krankenhaus und das wird auch noch renoviert und auf den neuesten Stand gebracht. Im Gegensatz dazu ist es vielleicht nicht so politisch toll verkaufbar und für die Bevölkerung messbar, wenn ich sage, ich habe auch in diesem Tal aber ein mobiles Team des Patienten rund um die Operation betreut, eben damit ich auch dort die Möglichkeit habe, dass der Patient eben und das ist ja mit ein Problem des Ganzen, dass wenn ich einen Patienten habe, der eine Anreise nach [entfernt] hat von 1,5 Stunden oder in der Dimension, dass ich zu dem sage, kommen sie her zu uns ins Krankenhaus, dann machen wir schön die Physiotherapie damit sie fit zur OP kommen, dann sagt er dann, wie soll ich das schaffen?"

*"I think it's always the case that if there are certain interests that come across well to the population and the successes can be presented, then the realisations work quite well and I think what I have the feeling works very well in this federal state is, of course, a bit provocative, but it's structural changes, So people are extremely keen to build, we are now building a new hospital, we are now building a new operating theatre in [removed], which nobody needs, and we are rebuilding the hospital in [removed] and when you see that we are now building a new building for this and that, so these things are such great successes that you can always sell well to the population as a*

*politician.*

*There is a hospital in every valley and it is being renovated and brought up to date. In contrast, it is perhaps not so politically sellable and measurable for the population if I say that I have a mobile team in this valley to look after the patient around the operation, so that I also have the opportunity to ensure that the patient - and that is one of the problems with the whole thing, that if I have a patient who has a journey of 1.5 hours or more to [far away], that I say to them, ‘Come here to our hospital and we'll do the physiotherapy so that they are fit for the operation, then they say, ’How am I supposed to manage that? "*

Code: ● Innovationsklima	Weight score: 0

C1 200224, 620 - 641

Created: 01.05.2024 21:38 by jmlut, Modified: 01.05.2024 21:38 by jmlut

Area: 1518	3.45%

23.

"Und ob das einfach eine Mentalitätssache ist und man das nicht rausbringen wird, und ein Skandinavier das einfach gar nicht in seinem Kopf hat und dass das selbstverständlich ist, dass das funktioniert."

*"And whether it's simply a matter of mentality and you won't get that out, and a Scandinavian simply doesn't have that in their head and that it's a matter of course that it works."*

Code: ● Innovationsklima	Weight score: 0

C1 200224, 733 - 735

Created: 01.05.2024 21:45 by jmlut, Modified: 01.05.2024 21:45 by jmlut

Area: 204	0.46%

5.2. Politik

1.

"Naja, weil so lange es nicht um Geld geht, ist es uninteressant, glaube ich."

*‘Well, because as long as it's not about money, I don't think it's interesting.’*

Code: ● Politik	Weight score: 0

PT1 140324, 399 - 399

Created: 20.05.2024 20:10 by Jan, Modified: 20.05.2024 20:10 by Jan

Area: 76	0.21%

2.

"Wie gesagt, du brauchst irgendein Sprachrohr oder irgendwas, was gewichtig ist, weil sonst, glaube ich, kommst du nicht weit. Also du machst dein eigenes Ding und stellst das selber auf die Beine, als Institution oder Privatperson oder was auch immer.
Und wenn das nachher ein positives Outcome hat und die anderen sehen das, dann kann es schon sein, dass das übernommen wird, aber sonst glaube ich, wenn du keinen Ansprechpartner hast oder jemanden, der das forciert und der Einfluss hat, wie zum Beispiel Ärztekammer sonst irgendwas, dann glaube ich, ist es recht schwierig."

*"As I said, you need some kind of mouthpiece or something that carries weight, because otherwise I don't think you'll get very far. So you do your own thing and set it up yourself, as an institution or a private individual or whatever.*

*And if it has a positive outcome afterwards and the others see it, then it's possible that it will be adopted, but otherwise I think if you don't have a contact person or someone who pushes it and has influence, such as the medical association or something else, then I think it's quite difficult."*

Code: ● Politik	Weight score: 0

PT1 140324, 604 - 613

Created: 20.05.2024 20:20 by Jan, Modified: 20.05.2024 20:20 by Jan

Area: 601	1.69%

3.

"das Wichtigste ist die politische Vernetzung."

*‘the most important thing is political networking.’*

Code: ● Politik	Weight score: 0

K1 280224, 338 - 339

Created: 09.05.2024 20:32 by jmlut, Modified: 09.05.2024 20:32 by jmlut

Area: 46	0.12%

4.

"Was haben wir noch uns überlegt? Aber vor allem ist es über die Patienten gegangen, die das dann auch eingefordert haben. Es ist wichtig, Pensionisten zu gewinnen. Pensionisten, das darf man nicht unterschätzen, sind eine große Wählerklientel. Und wenn die Pensionistenvertreter auf die Barikaden gehen, und die haben in allen den politischen Colouers gute und kräftige Vertreter drinnen, dann wird das auch diskutiert und dann wird der Druck auch auf die Versicherungen erhöht."

*"What else did we think about? But above all, it was the patients who demanded it. It's important to win over pensioners. Pensioners, and we shouldn't underestimate this, are a large electorate. And if the pensioners' representatives go on the barricades, and they have good and strong representatives in all the political parties, then this will also be discussed and the pressure on the insurance companies will also be increased."*

Code: ● Politik	Weight score: 0

K1 280224, 563 - 569

Created: 09.05.2024 20:42 by jmlut, Modified: 09.05.2024 20:42 by jmlut

Area: 484	1.30%

5.

"Also die Politik will immer Zahlen haben oder die Beamten wollen Zahlen haben. Das heißt, wenn man den Leitenden zeigen kann, dem Gesundheitswesen zeigen kann, dass es hier zum Beispiel trotz anfänglicher Investitionen, Mehrausgaben, zu verbesserten Outcomes führt.
Also etwas, wo die Landesregierung, Bundesregierung immer aufspringen ist, verkürzte Liegedauern im Krankenhaus, wenn man so was zeigen kann, dann hat man da sicher einen großen Stein im Brett, um noch weiter Mittel freizuspielen.
Das heißt aber auch, man muss einmal zuerst zeigen, dass es tatsächlich einen Benefit bringt mit dem vorgegebenen Konzept."

*"So politicians always want figures or civil servants want figures. In other words, if you can show the managers, show the healthcare system, for example, that it leads to improved outcomes despite initial investments and additional expenditure.*

*In other words, something that the state government and the federal government always jump on - shorter hospital stays - if you can show something like that, then you will certainly have a big stone in your court to free up more funds.*

*But that also means that you first have to show that there is actually a benefit with the given concept."*

Code: ● Politik	Weight score: 0

A1 210224, 352 - 362

Created: 09.05.2024 17:29 by jmlut, Modified: 09.05.2024 17:29 by jmlut

Area: 661	1.66%

6.

"Und wie gesagt, also für Österreich grundsätzlich sehe ich die Möglichkeiten, dass man über Fachgesellschaften geht, weil dann kommt der Druck von der Fachgesellschaftsseite oder eben von der Regierungsseite, wobei wenn es ein politischer Prozess sein muss, also der, wo dann die Beamten irgendwas aussprechen muss, die Politik entschieden haben, dann ist es meistens erst spät.
Und in der Regel folgt ja die Politik häufig auch den Meinungen der Bevölkerung oder Strömungen, die sich in der Bevölkerung eh schon sozusagen am durchsetzen sind. Das heißt, das Beste wäre wirklich, wenn hier von der Fachgesellschaft aus Empfehlungen entstehen."

*"And as I said, for Austria I basically see the possibility of going through professional associations, because then the pressure comes from the professional society side or from the government side, whereby if it has to be a political process, i.e. the one where the officials have to pronounce something, the politicians have decided, then it is usually only late.*

*And as a rule, politicians often follow the opinions of the population or currents that are already gaining ground in the population anyway, so to speak. In other words, the best thing would really be if recommendations were made by the specialist society."*

Code: ● Politik	Weight score: 0

A1 210224, 588 - 597

Created: 09.05.2024 18:03 by jmlut, Modified: 09.05.2024 18:03 by jmlut

Area: 668	1.68%

7.

"achgesellschaften auf jeden Fall, weil man ja für alles Personal braucht, Politik sehe ich unbedingt gefordert, weil da geht es um das Gemeinwohl, da geht es um Strukturen, die wir in der Zukunft schaffen müssen, um das alles zu bewältigen."

*‘In any case, I see politics as an absolute must, because it's about the common good, it's about structures that we need to create in the future in order to manage all of this.’*

Code: ● Politik	Weight score: 0

A2 220422, 234 - 237

Created: 09.05.2024 19:42 by jmlut, Modified: 09.05.2024 19:42 by jmlut

Area: 243	0.68%

8.

"Das Land und man müsste sagen wir machen ein Programm, sozusagen für unsere Bevölkerung, wo wir das einführen. Und wo uns das bewusst ist, dass das zu einer Verbesserung führt. Aber da wäre der Träger oder die Politik dann gefragt, sage ich jetzt einmal so."

*"The country and you would have to say we are making a programme, so to speak, for our population, where we introduce this. And where we are aware that this will lead to an improvement. But then it would be up to the funding body or the politicians, I'll put it that way."*

Code: ● Politik	Weight score: 0

C1 200224, 586 - 589

Created: 01.05.2024 21:37 by jmlut, Modified: 01.05.2024 21:37 by jmlut

Area: 260	0.59%

9.

"es sind so immer die, glaube ich, wenn gewisse Interessen da sind, eben die gut bei der Bevölkerung rüber kommen dann und die Erfolge quasi so präsentierbar sind, dann
funktionieren die Umsetzungen ganz gut und ich glaube das, was ich das Gefühl habe, was in diesem Bundesland sehr gut funktioniert, das ist natürlich ein bisschen provokativ, aber es sind bauliche Veränderungen, also es wird extrem gerne gebaut, wir bauen jetzt ein neues Krankenhaus, wir bauen jetzt in [entfernt] einen neuen OP, den kein Mensch braucht, und wir bauen in [entfernt] des Krankenhauses um und wenn man sieht, wir bauen jetzt ein neues Gebäude für dieses und jenes, also diese Dinge sind ja so schöne Erfolge, die man als
Politiker der Bevölkerung immer gut verkaufen kann.
Also in jedem Tal steht ein Krankenhaus und das wird auch noch renoviert und auf den neuesten Stand gebracht. Im Gegensatz dazu ist es vielleicht nicht so politisch toll verkaufbar und für die Bevölkerung messbar, wenn ich sage, ich habe auch in diesem Tal aber ein mobiles Team des Patienten rund um die Operation betreut, eben damit ich auch dort die Möglichkeit habe, dass der Patient eben und das ist ja mit ein Problem des Ganzen, dass wenn ich einen Patienten habe, der eine Anreise nach [entfernt] hat von 1,5 Stunden oder in der Dimension, dass ich zu dem sage, kommen sie her zu uns ins Krankenhaus, dann machen wir schön die Physiotherapie damit sie fit zur OP kommen, dann sagt er dann, wie soll ich das schaffen?"

*"I think that if there are certain interests that come across well to the population and the successes can be presented, then*

*the realisations work quite well and I think that what I feel works very well in this federal state is of course a bit provocative, but it's structural changes, So people are extremely keen to build, we are now building a new hospital, we are now building a new operating theatre in [removed], which nobody needs, and we are rebuilding the hospital in [removed] and when you see that we are now building a new building for this and that, so these things are such great successes that you can always sell well to the population as a*

*politician.*

*There's a hospital in every valley and it's being renovated and brought up to date. In contrast, it is perhaps not so politically sellable and measurable for the population if I say that I also have a mobile team in this valley to look after the patient around the operation, so that I also have the possibility there that the patient - and that is one of the problems with the whole thing - can be treated in the same way, that if I have a patient who has a journey of 1.5 hours or more to [far away], that I say to them, come to our hospital and we'll do the physiotherapy so that they're fit for the operation, then they say, "How am I supposed to manage that? "*

Code: ● Politik	Weight score: 0

C1 200224, 620 - 641

Created: 01.05.2024 21:38 by jmlut, Modified: 01.05.2024 21:38 by jmlut

Area: 1518	3.45%

5.3. Einstellung Bevölkerung / Patienten

1.

"es ist halt auch das große Problem ist, dass die Leute immer nur dann was machen, wenn der Hut brennt."

*"The big problem is that people only do something when their hats are on fire."*

Code: ● Einstellung Bevölkerung / Patienten	Weight score: 0

PT1 140324, 131 - 132

Created: 20.05.2024 20:01 by Jan, Modified: 20.05.2024 20:01 by Jan

Area: 103	0.29%

2.

"Wenn es mir so schlecht geht, dass ich was tun musst, dann geh ich zum Therapeuten und sonst alles was was kostet, wird eigentlich negiert und wird halt einfach nicht gern angenommen."

*‘If I'm feeling so bad that I need to do something, then I go to a therapist and anything else that costs money is actually negated and simply not accepted.’*

Code: ● Einstellung Bevölkerung / Patienten	Weight score: 0

PT1 140324, 136 - 138

Created: 20.05.2024 20:02 by Jan, Modified: 20.05.2024 20:02 by Jan

Area: 185	0.52%

3.

"Wie gesagt, ich glaube, das es ganz gut wäre, wenn du da irgendein Zentrum machen würdest oder so, weil im Krankenhaus bist du ja immer schon wieder in dieser Krankenhausanstalt. Das heißt, du musst immer wieder ins Krankenaus hinein, und ich weiß nicht, ob das so angenehm ist für die Leute.
Also mit dem Krankenhaus wird bei uns ja nicht das absolut Positive verbunden, sondern ins Krankenhaus gehst du nur, wenn es dir schlecht geht. Wenn du dann dreimal die Woche hingehen soll, weiß ich nicht, ob die Compliance nachher so ..."

*"As I said, I think it would be quite good if you did some kind of centre or something, because in hospital you're always back in this hospital. That means you always have to go back to the hospital, and I don't know if that's so pleasant for people.*

*Hospital isn't associated with anything positive in our country, you only go to hospital when you're unwell. If you have to go there three times a week, I don't know whether the compliance afterwards is so ..."*

Code: ● Einstellung Bevölkerung / Patienten	Weight score: 0

PT1 140324, 208 - 215

Created: 20.05.2024 20:02 by Jan, Modified: 20.05.2024 20:02 by Jan

Area: 554	1.55%

4.

"Da müssen sie da jedes Mal hin und her fahren. Gerade wenn du von [entfernt] ausgehst und die sind aus der Stadt, dann kannst du jedes Mal rechtnen, dreiviertel Stunden hin, dreiviertel Stunde zurück und dann hast du dreiviertel Stunde Therapie.
Das heißt, du hast immer den ganzen Vormittag oder Nachmittag, kannst du eigentlich abschreiben."

*"They have to drive back and forth every time. Especially if you go out from [far away] and they are from the city, then you can calculate every time, three quarters of an hour there, three quarters of an hour back and then you have three quarters of an hour of therapy.*

*That means you always have the whole morning or afternoon, you can actually write it off."*

Code: ● Einstellung Bevölkerung / Patienten	Weight score: 0

PT1 140324, 221 - 227

Created: 20.05.2024 20:03 by Jan, Modified: 20.05.2024 20:03 by Jan

Area: 364	1.02%

5.

"Also wenn du überall hinfahren musst, glaube ich, dass das einfach sehr zeitintensiv ist. Wenn du eh schon weißt, dass du eine schwere Operation hast oder sonstwas, dann weiß ich nicht, ob das jetzt vor der Psyche oder halt von der Compliance nachher da ist, dass die sagen, ja was, ich fahre jetzt dann dreimal pro Woche alle drei Stationen ab.
Ja. Also das glaube ich nicht, einfach, wie gesagt, wenn der Hut total brennt, Ja, aber wenn sie sagen mir geht es eh gut und ich fühle mich einigermaßen wohl, dann glaube ich das nicht, dass das funktioniert."

*"So if you have to travel everywhere, I think it's simply very time-consuming. If you already know that you're having a serious operation or something else, then I don't know whether it's because of the psyche or because of the compliance afterwards, that they say, yes, what, I'm going to travel to all three wards three times a week.*

*Yes. So I don't believe that, simply, as I said, if the hat is totally on fire, yes, but if they say I'm fine anyway and I feel reasonably well, then I don't think that will work."*

Code: ● Einstellung Bevölkerung / Patienten	Weight score: 0

PT1 140324, 247 - 255

Created: 20.05.2024 20:04 by Jan, Modified: 20.05.2024 20:04 by Jan

Area: 579	1.62%

6.

"ich würde jetzt einmal sagen, dass in Österreich die Prävention sowieso total schlecht ist. Da müsste ein Umdenken anfangen"

*"I would say that prevention is totally bad in Austria anyway. There needs to be a rethink"*

Code: ● Einstellung Bevölkerung / Patienten	Weight score: 0

PT1 140324, 342 - 343

Created: 20.05.2024 20:09 by Jan, Modified: 20.05.2024 20:09 by Jan

Area: 124	0.35%

7.

"das du halt sowohl die Politik als auch halt diese Sozialversicherungsträger auf deine Seite bringst, so dass das nachher bezahlt wird oder dass die Leute das lukrieren können, weil sonst glaube ich selbst zahlen, wie gesagt, ist in Österreich ganz mau, finde ich."

*‘That you get both the politicians and the social insurance providers on your side, so that it is paid for afterwards or that people can earn it, because otherwise I think paying for it yourself, as I said, is very poor in Austria, I think.’*

Code: ● Einstellung Bevölkerung / Patienten	Weight score: 0

PT1 140324, 411 - 414

Created: 20.05.2024 20:11 by Jan, Modified: 20.05.2024 20:11 by Jan

Area: 267	0.75%

8.

"In Österreich ist es so, das haben wir die letzten 150 Jahre gemacht, also machen wir es die nächsten 150 Jahre auch so."

*‘In Austria, we've done it this way for the last 150 years, so we'll do it the same way for the next 150 years.’*

Code: ● Einstellung Bevölkerung / Patienten	Weight score: 0

PT1 140324, 444 - 445

Created: 20.05.2024 20:18 by Jan, Modified: 20.05.2024 20:18 by Jan

Area: 121	0.34%

9.

"Ja, der größte Punkt ist Bewusstseinsbildung. Also wenn ich jetzt gar nicht weiß, wie soll ich dann überhaupt jemand das raten?
Weil, ich sage jetzt mal, man könnte ja vielleicht dann auch sagen, aha, Sie haben eine Operation, wollen Sie sich da im Vorfeld mal genau informieren? Mit wem, oder auch, dass man es mal im Haus bespricht?
Mit wem könnten wir denn das organisieren? Also ich glaube, das Bewusstsein, bilden. Ich fände es gut, wenn du mal einen Vortrag halten würdest, im internen Ärzte-Jour-Fix oder auch überhaupt, einmal öffentlich ausgeschrieben in unserem Haus, vielleicht sogar auch überhaupt öffentlich für die ganze Region einen Vortrag halten, dass man überhaupt die Leute informiert, was ist das, wie wirkt das, was bringt das. Also Bewusstsein."

*"Yes, the biggest point is awareness-raising. So if I don't know at all, how am I supposed to advise someone?*

*Because, I'm just saying, you could perhaps say, aha, you're having an operation, do you want to find out more in advance? With whom, or that it should be discussed in-house?*

*Who could we organise this with? Well, I think we need to raise awareness. I think it would be good if you were to give a talk, in the internal doctors' meeting or even publicly advertised in our organisation, perhaps even give a public talk for the whole region, so that people are informed about what it is, how it works and what the benefits are. So awareness."*

*Code: ● Einstellung Bevölkerung / Patienten	Weight score: 0*

P1 010324, 200 - 212

Created: 20.05.2024 18:28 by Jan, Modified: 20.05.2024 18:28 by Jan

Area: 809	2.11%

10.

"Das tun wir schön verdrängen. Wer will schon gern dieses Tabu rund um Tod und Zwischenfälle? Das wollen wir doch lieber mal verdrängen. Das lassen wir alles auf uns zukommen. Also so nach dem Motto ich setze mich in ein Flugzeug als Pilot und bin nicht vorbereitet.
Ja, und dann kommt ein Gewitter und ein Triebwerksausfall, aber ich habe keine Ahnung, keine Checkliste, keinen Plan. Also bis auf diese Vogelstraußpolitik, glaube ich, hat auch was damit zu tun.
Auch der Umgang mit Ängsten in unserer Gesellschaft. Also das ändert sich zwar jetzt ordentlich. Also ich habe das Gefühl, der Heldenmythos stirbt eh schon langsam, musst der starke Mann sein und die starke Frau und darf keine Ängste haben.
Aber schon, ich denke, das ist auch noch ein Grund, dass jetzt wenn im Alltag jemand sagt: "ja, ich habe da einen Magenbruch, Darmbruch, Leistenbruch - sagen wir mal Leistenbruch, muss ja nicht was Riesiges sein. Ja, Leistenbruch-OP, ja, na, ist eine Kleinigkeit, sehr klar erledigt. Ja, und so reden die in der Familie oder unter Freunden. Und das war es dann. Da fragt dann keiner, hast du Angst oder wie läuft denn das genau ab?
Ja und und dass das vielleicht auch ein Grund ist, dass wir doch dazu neigen, alles, was Krankheit, Operationen betrifft, zu verdrängen."

*"We do a good job of suppressing that. Who wants this taboo around death and incidents? We'd rather suppress that. We'll let it all come to us. In other words, I get on an aeroplane as a pilot and I'm not prepared.*

*Yes, and then there's a thunderstorm and an engine failure, but I have no idea, no checklist, no plan. So apart from this bird's-eye policy, I think that has something to do with it.*

*Also the way we deal with fears in our society. So that's changing quite a bit now. I have the feeling that the hero myth is slowly dying anyway, that you have to be the strong man and the strong woman and not have any fears.*

*But yes, I think that's another reason that now when someone says in everyday life: "Yes, I have a stomach hernia, bowel hernia, inguinal hernia - let's say a hernia, it doesn't have to be something huge. Yes, inguinal hernia surgery, yes, well, it's a small thing, very clearly done. Yes, and that's how they talk in the family or among friends. And that's it. Nobody asks, are you scared or how exactly does it work?*

*Yes, and that's perhaps also a reason why we tend to suppress everything that concerns illness and operations."*

Code: ● Einstellung Bevölkerung / Patienten	Weight score: 0

P1 010324, 253 - 276

Created: 20.05.2024 18:48 by Jan, Modified: 20.05.2024 18:48 by Jan

Area: 1375	3.59%

11.

"ich glaube, dass es Patienten gibt, die sagen, es reicht mir eine medizinische Info, das finde ich gut, das hole ich mir. Dann glaube ich, dass es Patienten gibt, die sagen, je mehr ich darüber weiss, desto mehr rege ich mich auf, gescheiter: gehe dahin, erledigt."

*"I believe that there are patients who say that medical information is enough for me, I think it's good, I'll get it. Then I believe that there are patients who say that the more I know about it, the more upset I get, the better: go there, done."*

Code: ● Einstellung Bevölkerung / Patienten	Weight score: 0

P1 010324, 301 - 304

Created: 20.05.2024 18:51 by Jan, Modified: 20.05.2024 18:51 by Jan

Area: 267	0.70%

12.

"Zu wenig Öffentlichkeitsarbeit. Mir fällt sofort Radio ein, Radio [entfernt] ein Beitrag zur Prähabilitation. Einen öffentlichen Vortrag, Berichte in den Gemeindezeitungen"

*"Too little public relations work. I immediately think of radio, radio [removed] a contribution to prehabilitation. A public lecture, reports in the community newspapers"*

Code: ● Einstellung Bevölkerung / Patienten	Weight score: 0

P1 010324, 395 - 398

Created: 20.05.2024 18:53 by Jan, Modified: 20.05.2024 18:53 by Jan

Area: 189	0.49%

13.

"Das würde erst mal das Bewusstsein erweitern. Ach so, gar vor der Operation, da um mich zu informieren. Ich sage jetzt mal wie ein Geburtsvorbereitungskurs."

*"That would first increase awareness. Oh, even before the operation, to inform myself. I'm going to say it's like a antenatal course."*

Code: ● Einstellung Bevölkerung / Patienten	Weight score: 0

P1 010324, 402 - 403

Created: 20.05.2024 18:54 by Jan, Modified: 20.05.2024 18:54 by Jan

Area: 157	0.41%

14.

"Der größte Grund psychologisch ist sicher der Verdrängungsmechanismus."

*‘The biggest psychological reason is certainly the repression mechanism.’*

Code: ● Einstellung Bevölkerung / Patienten	Weight score: 0

P1 010324, 594 - 594

Created: 20.05.2024 18:57 by Jan, Modified: 20.05.2024 18:57 by Jan

Area: 70	0.18%

15.

"Wenn der Rahmen nicht geschaffen wird, weil sie es nicht wollen, dann gehört Medienarbeit gemacht, damit der Druck steigt.
Denke ich, Öffentlichkeitsarbeit gemacht, damit mal Patienten sagen, wieso gibt es das nicht und so, dass der Druck steigt. Und dann gehört der Rahmen geschaffen."

*"If the framework is not created because they don't want it, then media work needs to be done to increase the pressure.*

*I think public relations work needs to be done so that patients say why isn't this available and so that the pressure increases. And then the framework needs to be created."*

Code: ● Einstellung Bevölkerung / Patienten	Weight score: 0

P1 010324, 646 - 651

Created: 20.05.2024 18:58 by Jan, Modified: 20.05.2024 18:58 by Jan

Area: 306	0.80%

16.

"Und da glaube ich, dass die Patienten, wenn sie diese Diagnose bekommen und der Schock da so groß ist, sei es sie müssen hüfttoperiert werden, sei es sie brauchen eine Niere, sei es sie müssen jetzt eine Bypass-Operation haben. Dass sie in dieser Zeit, die Patienten, wenn der allererste Schreck vorbei ist und sogar den könnte man ihnen nehmen, indem man, das ist jetzt meine Idee dazu, als erstes schon einen Psychologen, der dahingehend auch gut mit - oder ein Sozialarbeiter - der dahingehend gut geschult ist, und den Patienten in diese Richtung leitet und ihm sagt, dort und dort und dort können sie sich diese Hilfe holen und so eine Prähabilitation wäre eben eine gute Hilfe für Patienten, dass sie sich vorher erstens... kommen da dann Fragen auf, die sie vielleicht vorher nicht gestellt haben."

*"And I believe that when patients receive this diagnosis and the shock is so great, whether they need a hip operation, a kidney transplant or a bypass operation, they should be given a psychologist to help them during this time. I think it would be a good idea for patients, once the initial shock is over, to have a psychologist who is well trained in this area - or a social worker - who can guide the patient in this direction and tell them that they can get help there and there and there, and that prehabilitation would be a good way of helping patients to firstly... questions come up that they may not have asked before."*

Code: ● Einstellung Bevölkerung / Patienten	Weight score: 0

K1 280224, 488 - 498

Created: 09.05.2024 20:40 by jmlut, Modified: 09.05.2024 20:40 by jmlut

Area: 814	2.19%

17.

"Ja, schon dann Patientenunzufriedenheit und Patientenzufriedenheit, beides. Also Patienten zu aktivieren, dass sie sich da auch dafür engagieren und das auch in Zeitschriften bringen, dass man darüber öffentlich diskutiert, dass man Diskussionsveranstaltungen dazu veranstaltet, dass man in Zeitungen oder jetzt sind sicher die Medien, egal nicht Zeitungen, werden nicht mehr so gelesen, aber Medienlandschaften, ja, eventuell auch im Krankenhaus dieses Wartezimmer-TV, dass man dort auch das einfach schon anbietet und den Patienten zeigt, diese Möglichkeit gibt es, also die Patienten zu informieren, die Niedergelassenen zu informieren, Krankenhausärzte dafür zu gewinnen, dass das eine gute Sache ist."

*"Yes, patient dissatisfaction and patient satisfaction, both. So activating patients, getting them to get involved in this and getting it into magazines, discussing it in public, organising discussion events about it, publishing it in newspapers or now it's certainly the media, not newspapers, they're not read like that anymore, but media landscapes, yes, possibly also in hospitals, this waiting room TV, simply offering it there and showing patients that there is this possibility, i.e. informing patients, informing practitioners, winning over hospital doctors to the fact that this is a good thing."*

Code: ● Einstellung Bevölkerung / Patienten	Weight score: 0

K1 280224, 553 - 561

Created: 09.05.2024 20:42 by jmlut, Modified: 09.05.2024 20:42 by jmlut

Area: 713	1.92%

18.

"Was haben wir noch uns überlegt? Aber vor allem ist es über die Patienten gegangen, die das dann auch eingefordert haben. Es ist wichtig, Pensionisten zu gewinnen. Pensionisten, das darf man nicht unterschätzen, sind eine große Wählerklientel. Und wenn die Pensionistenvertreter auf die Barikaden gehen, und die haben in allen den politischen Colouers gute und kräftige Vertreter drinnen, dann wird das auch diskutiert und dann wird der Druck auch auf die Versicherungen erhöht."

*"What else did we think about? But above all, it was the patients who demanded it. It's important to win over pensioners. Pensioners, and we shouldn't underestimate this, are a large electorate. And if the pensioners' representatives go on the barricades, and they have good and strong representatives in all the political parties, then this will also be discussed and the pressure on the insurance companies will also be increased."*

Code: ● Einstellung Bevölkerung / Patienten	Weight score: 0

K1 280224, 563 - 569

Created: 09.05.2024 20:42 by jmlut, Modified: 09.05.2024 20:42 by jmlut

Area: 484	1.30%

19.

"Und das können wir auch in Informationsveranstaltungen... bzw. ein wichtiger Faktor ist immer auch die Mundpropaganda. Also positive Mundpropaganda ist immer etwas, was sehr viele Patienten weiter bringt."

*"And we can also do this at information events... and word of mouth is always an important factor. So positive word of mouth is always something that helps a lot of patients."*

Code: ● Einstellung Bevölkerung / Patienten	Weight score: 0

K1 280224, 597 - 599

Created: 09.05.2024 20:42 by jmlut, Modified: 09.05.2024 20:42 by jmlut

Area: 206	0.56%

20.

"Ich nehme wahr, dass die Compliance eine gewisse Herausforderung ist und da auch versucht wird, mit Apps zu arbeiten, die sozusagen Feedback geben, also erinnern bzw. dann auch Feedback geben lassen, ob man das Ganze dann gemacht hat oder nicht. Ich glaube, da gibt es die unterschiedlichsten Patienten,"

*"I realise that compliance is a certain challenge and that attempts are also being made to work with apps that provide feedback, so to speak, i.e. reminders or feedback as to whether or not the whole thing has been done. I think there are all kinds of different patients."*

Code: ● Einstellung Bevölkerung / Patienten	Weight score: 0

A1 210224, 546 - 549

Created: 09.05.2024 18:01 by jmlut, Modified: 09.05.2024 18:01 by jmlut

Area: 306	0.77%

21.

"Und die Patienten selber sind schon deutlich aufgeklärter, als sie es noch vor einigen Jahren waren. Aber auch immer noch zu einem guten Teil sagen, der Arzt soll mal machen, der wird schon wissen, was gut ist. Ich selber bitte nein."

*"And the patients themselves are much more informed than they were a few years ago. But a large proportion of them still say, let the doctor do it, he'll know what's good. I myself say no, please."*

Code: ● Einstellung Bevölkerung / Patienten	Weight score: 0

A2 220422, 306 - 308

Created: 09.05.2024 19:45 by jmlut, Modified: 09.05.2024 19:45 by jmlut

Area: 235	0.66%

22.

"Ja, ich tu da jetzt einmal nicht wirklich was, oder sehe gar überhaupt nicht ein, warum muss ich das jetzt machen? Ja, ich bin zwar sehr gesund, ich nehme nur 17 Tabletten pro Tag und ich bin topfit.
Und das ist so ein, vielleicht ist es tatsächlich etwas Österreich spezifisch und kulturell auch das Abgeben von Verantwortung. Das muss jemand anderes für mich machen, ich tu da nichts machen."

*“Yes, I'm not really doing anything now, or I don't even realise why I have to do it now? Yes, I'm very healthy, I only take 17 tablets a day and I'm in great shape.*

*And that's such a, maybe it's actually a bit Austria-specific and culturally also the relinquishing of responsibility. Someone else has to do that for me, I don't do anything."*

Code: ● Einstellung Bevölkerung / Patienten	Weight score: 0

A2 220422, 312 - 318

Created: 09.05.2024 19:45 by jmlut, Modified: 09.05.2024 19:45 by jmlut

Area: 415	1.16%

23.

"Dazu müsste man der Bevölkerung ja noch viel intensiver beibringen, was sie bei großen Operationen erwartet. Die kommen ja manchmal, ist mir aufgefallen in der präoperativen Ambulanz, für wirklich große Operationen, die auch so bezeichnet werden, und haben den Eindruck, das ist was, da komm ich rein, halbe Stunde OP und alles ist gut, also eine völlig falsche Vorstellung, was das bedeutet, auch für den Körper, dass es große Operationen sind.
Also die sind einfach nicht aufgeklärt, was auf sie zukommt, oder sind sich dessen gar nicht bewusst, was das für den Körper bedeutet und daher natürlich auch überhaupt nicht ingestellt oder bereit, ihre wirkliche körperliche Fitness einmal kritisch zu hinterfragen."

*"You would have to teach the population much more intensively about what to expect during major operations. Sometimes, I've noticed in the pre-operative outpatient clinic, they come for really major operations, which are also referred to as such, and have the impression that this is something, I come in, half an hour of surgery and everything is fine, so they have a completely wrong idea of what this means, also for the body, that these are major operations.*

*So they simply don't know what they're in for, or they're not even aware of what it means for the body and are therefore naturally not at all prepared or willing to critically scrutinise their actual physical fitness."*

Code: ● Einstellung Bevölkerung / Patienten	Weight score: 0

A2 220422, 323 - 333

Created: 09.05.2024 19:45 by jmlut, Modified: 09.05.2024 19:45 by jmlut

Area: 739	2.07%

24.

"Im Gegensatz dazu ist es vielleicht nicht so politisch toll verkaufbar und für die Bevölkerung messbar, wenn ich sage, ich habe auch in diesem Tal aber ein mobiles Team des Patienten rund um die Operation betreut, eben damit ich auch dort die Möglichkeit habe, dass der Patient eben und das ist ja mit ein Problem des Ganzen, dass wenn ich einen Patienten habe, der eine Anreise nach [entfernt] hat von 1,5 Stunden oder in der Dimension, dass ich zu dem sage, kommen sie her zu uns ins Krankenhaus, dann machen wir schön die Physiotherapie damit sie fit zur OP kommen, dann sagt er dann, wie soll ich das schaffen?
Ich schaffe es ja kaum sozusagen, einkaufen ist für mich schon beschwerlich, wie soll da quasi herkommen und schauen, dass ich da Physio und etc. mache, damit ich besser in die OP hinein gehe. Und da glaube ich, ist es auch so, dass es vielleicht der Grund ist, dass man das nicht so gut verkaufen kann, dass das gemacht wird, weil das halt nichts Sichtbares ist oder so, aber das ist immer so ein Gedankenspiel."

*"In contrast, it's perhaps not so politically sellable and measurable for the population if I say that I also have a mobile team in this valley to look after the patient around the operation, so that I also have the opportunity there for the patient - and that's one of the problems with the whole thing, that if I have a patient who has a journey of 1.5 hours or more to [far away], that I say to them, come here to our hospital and we'll do the physiotherapy so that they're fit for the operation, then they say, how am I supposed to manage that?*

*I can hardly manage it, so to speak, shopping is already difficult for me, how am I supposed to come here and make sure that I do physiotherapy and so on so that I can go into the operation better? And I think that's perhaps the reason why it's not so easy to sell that it's being done, because it's not something visible or anything, but that's always a mind game."*

Code: ● Einstellung Bevölkerung / Patienten	Weight score: 0

C1 200224, 633 - 648

Created: 01.05.2024 21:39 by jmlut, Modified: 01.05.2024 21:39 by jmlut

Area: 1058	2.41%

25.

"Und ob das einfach eine Mentalitätssache ist und man das nicht rausbringen wird, und ein Skandinavier das einfach gar nicht in seinem Kopf hat und dass das selbstverständlich ist, dass das funktioniert."

*‘And whether it's simply a matter of mentality and you won't get that out, and a Scandinavian simply doesn't have that in their head and that it's a matter of course that it works.’*

Code: ● Einstellung Bevölkerung / Patienten	Weight score: 0

C1 200224, 733 - 735

Created: 01.05.2024 21:44 by jmlut, Modified: 01.05.2024 21:44 by jmlut

Area: 204	0.46%

26.

"Es kann schwierig sein, wenn die verschiedenen Ambulanzzeiten sich entweder überschneiden oder nicht überschneiden, dass es für den Patienten sehr aufwendig wird und damit die Patientenmotivation leidet."

*‘It can be difficult if the different outpatient clinic times either overlap or don't overlap, making it very time-consuming for the patient and thus reducing patient motivation.’*

Code: ● Einstellung Bevölkerung / Patienten	Weight score: 0

C2 180324, 297 - 299

Created: 20.05.2024 21:23 by Jan, Modified: 20.05.2024 21:23 by Jan

Area: 205	0.60%

27.

"So dass es für den Patienten im Haus möglichst wenig Mehraufwand bedeutet, dann glaube ich, kann man mehr Patienten dafür motivieren."

*‘So that it means as little extra work as possible for the patient in the clinic, then I think you can motivate more patients to do it.’*

Code: ● Einstellung Bevölkerung / Patienten	Weight score: 0

C2 180324, 556 - 557

Created: 20.05.2024 21:45 by Jan, Modified: 20.05.2024 21:45 by Jan

Area: 134	0.39%

28.

"natürlich denke ich an die, die von weit her kommen, die kann man wohl schwer dazu zwingen, dass sie eine halbe Stunde oder Dreiviertelstunde lang fahren, das geht glaube ich nicht. Das ist halt dann eine Frage, ob sie dann trotzdem motiviert sein werden."

*"Of course I'm thinking of those who come from far away, it's difficult to force them to drive for half an hour or three quarters of an hour, I don't think that's possible. It's just a question of whether they will still be motivated."*

Code: ● Einstellung Bevölkerung / Patienten	Weight score: 0

C2 180324, 563 - 567

Created: 20.05.2024 21:46 by Jan, Modified: 20.05.2024 21:46 by Jan

Area: 275	0.81%

6. Einstellung zur Prähabilitation

6.1. Sinnhaftigkeit der Prähabilitation

1.

"Und da glaube ich, dass die Patienten, wenn sie diese Diagnose bekommen und der Schock da so groß ist, sei es sie müssen hüfttoperiert werden, sei es sie brauchen eine Niere, sei es sie müssen jetzt eine Bypass-Operation haben. Dass sie in dieser Zeit, die Patienten, wenn der allererste Schreck vorbei ist und sogar den könnte man ihnen nehmen, indem man, das ist jetzt meine Idee dazu, als erstes schon einen Psychologen, der dahingehend auch gut mit - oder ein Sozialarbeiter - der dahingehend gut geschult ist, und den Patienten in diese Richtung leitet und ihm sagt, dort und dort und dort können sie sich diese Hilfe holen und so eine Prähabilitation wäre eben eine gute Hilfe für Patienten, dass sie sich vorher erstens... kommen da dann Fragen auf, die sie vielleicht vorher nicht gestellt haben."

*"And I believe that when patients receive this diagnosis and the shock is so great, whether they need a hip operation, a kidney transplant or a bypass operation, they should be given a psychologist to help them during this time. I think it would be a good idea for patients, once the initial shock is over, to have a psychologist who is well trained in this area - or a social worker - who can guide the patient in this direction and tell them that they can get help there and there and there, and that prehabilitation would be a good way of helping patients to firstly... questions come up that they may not have asked before."*

Code: ● Sinnhaftigkeit der Prähabilitation	Weight score: 0

K1 280224, 488 - 498

Created: 09.05.2024 20:39 by jmlut, Modified: 09.05.2024 20:39 by jmlut

Area: 814	2.19%

2.

"Sie haben das Gefühl, okay, ich bin jetzt in der richtigen Schleuse und dort, mein Ziel ist dann die bestmögliche Versorgung in der Operation, in der Intervention, was auch immer. Und ich glaube, dass das wirklich sogar der richtige Zeitpunkt ist, wo ein Patient am allermeisten motiviert ist, sich Hilfe zu holen, und dort auch etwas zu machen, weil niemand, der sich operieren lässt - ist nicht so, dass ihm das ganz egal ist, sondern jeder hat ein bisschen Angst davor. Es ist eine Unsicherheit, es ist ein organisatorischer Aufwand."

*"They have the feeling, okay, I'm now in the right lock and there, my goal is then the best possible care in the operation, in the intervention, whatever. And I believe that this is actually the right time when a patient is most motivated to get help and to do something there, because nobody who has an operation - it's not that they don't care at all, but everyone is a bit scared of it. It's an uncertainty, it's an organisational effort."*

Code: ● Sinnhaftigkeit der Prähabilitation	Weight score: 0

K1 280224, 500 - 506

Created: 09.05.2024 20:40 by jmlut, Modified: 09.05.2024 20:40 by jmlut

Area: 542	1.46%

3.

"Alle diese Dinge, denke ich, sind in der Peähabilitation auch dann - das ist auch das nächste, was mir als Idee kommt: ich glaube, die Krankenhausärzte wären damit sogar entlastet, weil sie einen besser vorbereiteten Patienten bekommen."

*“I think all of these things are also possible in prehabilitation - and that's the next thing that comes to mind: I think it would even take the pressure off the hospital doctors because they would have a better-prepared patient."*

Code: ● Sinnhaftigkeit der Prähabilitation	Weight score: 0

K1 280224, 528 - 530

Created: 09.05.2024 20:41 by jmlut, Modified: 09.05.2024 20:41 by jmlut

Area: 238	0.64%

4.

"Alle diese Dinge, denke ich, sind durchaus, was gute Faktoren wären, um die Patienten optimal vorzubereiten, aber auch den Ärzten im Krankenhaus eine Entlastung und nicht eine Belastung zu schaffen."

*‘All of these things, I think, are definitely good factors for preparing patients in the best possible way, but also for relieving the burden on doctors in the hospital rather than creating a burden.’*

Code: ● Sinnhaftigkeit der Prähabilitation	Weight score: 0

K1 280224, 542 - 544

Created: 09.05.2024 20:41 by jmlut, Modified: 09.05.2024 20:41 by jmlut

Area: 200	0.54%

5.

"Und die Prähabilitation ist dann natürlich ein extra wichtiger Schritt dazu zur Optimierung des Patienten-Outcomes postoperativ."

*‘And prehabilitation is of course an extra important step in optimising the patient outcome postoperatively.’*

Code: ● Sinnhaftigkeit der Prähabilitation	Weight score: 0

A1 210224, 56 - 57

Created: 22.06.2024 16:02 by Jan, Modified: 22.06.2024 16:02 by Jan

Area: 129	0.32%

6.

"Anstrengung es regelhaft umzusetzen, hat es gerade von der Narkoseambulanz sehr wohl gegeben, auch immer wieder Gespräche mit den operierend tätigen Kollegen, wobei da auch die Erfahrung war, dass zu einem höheren Prozent, die Kollegen, das als nicht wesentlich erachtet haben, eine Optimierung anzustreben und von der Seite her die Bemühungen nicht wirklich intensiv waren, das zu verbessern oder das im Vorfeld herauszufiltern."

*‘Efforts to implement it on a regular basis have certainly been made by the anaesthesia outpatient department in particular, and there have been repeated discussions with colleagues working in surgery, whereby the experience has also been that a higher percentage of colleagues have not considered it essential to strive for optimisation and from that side the efforts were not really intensive to improve it or to filter it out in advance.’*

Code: ● Sinnhaftigkeit der Prähabilitation	Weight score: 0

A2 220422, 78 - 83

Created: 09.05.2024 19:36 by jmlut, Modified: 09.05.2024 19:36 by jmlut

Area: 434	1.22%

7.

"Beide Aspekte, glaube ich, sind da unterschiedlich zum Tragen gekommen, aber in erster Linie ist unsinnig, ist nicht unbedingt notwendig, das geht schon."

*‘Both aspects, I think, have come into play in different ways, but first and foremost it's nonsensical, it's not absolutely necessary, it's possible.’*

Code: ● Sinnhaftigkeit der Prähabilitation	Weight score: 0

A2 220422, 95 - 96

Created: 09.05.2024 19:36 by jmlut, Modified: 09.05.2024 19:36 by jmlut

Area: 154	0.43%

8.

"Nein, also, offensichtlich besprechen wir immer wieder Teile davon. Also ich hätte gerne sozusagen mit meinen Patienten eben - weil das ja auch eines meiner Spezialgebiete ist, der obere Gastrointestinaltrakt, dann ist es ja so, dass die meistens aufgrund der Tumorstenose im schlechten Allgemeinzustand und Ernährungszustand sind, dann kriegen sie ja auch noch Chemotherapie und werden dann eigentlich von der Diagnosestellung bis dann unter der Chemotherapie oder der neoadjuvanten Therapie dann eigentlich schlechter.
Und wir sind da subjektiv nicht gut. Und wir reden dann immer darüber, was könnten wir denn darüber verbessern und da sind dann immer so kurze Initiativen mit, ja wir machen bei jedem von diesen Patienten ein Diätkonsil, ein Ernährungskonsil."

*"No, well, obviously we always discuss parts of it. So I would have liked to discuss with my patients - because this is also one of my specialities, the upper gastrointestinal tract - that they are usually in a poor general condition and nutritional status due to the tumour stenosis, then they also receive chemotherapy and then actually get worse from the time of diagnosis until they receive chemotherapy or neoadjuvant therapy.*

*And we are subjectively not good at that. And then we always talk about what we could improve and there are always such short initiatives with, yes, we do a diet consultation, a nutrition consultation with each of these patients."*

Code: ● Sinnhaftigkeit der Prähabilitation	Weight score: 0

C1 200224, 118 - 129

Created: 01.05.2024 16:17 by jmlut, Modified: 01.05.2024 16:17 by jmlut

Area: 790	1.80%

9.

"Also, ich glaube schon, dass es Unterstützung gibt. Zumindest in dem Moment wo ich es - jetzt angenommen, das ist jetzt eh so, dass habe ich jetzt mit dem ERAS, wenn man das jetzt umlegen würde, Prähabilitation, und ich gehe zu meinem Chef und sage, das sollen wir machen, sagt er gut super Idee, machen Sie das. Gut mache ich."

*"Well, I do believe that there is support. At least at the moment when I do it - assuming that's the case now anyway, that's what I have now with the ERAS, if you were to change it now, prehabilitation, and I go to my boss and say we should do that, he says well great idea, do it. I'll do it."*

Code: ● Sinnhaftigkeit der Prähabilitation	Weight score: 0

C1 200224, 379 - 383

Created: 01.05.2024 16:37 by jmlut, Modified: 01.05.2024 16:37 by jmlut

Area: 331	0.75%

10.

"Gedanken eben, dass das eigentlich wichtig wäre und ich hoffe, dass dein Projekt dann - vielleicht kommt da was raus, was dann zu einer Verbesserung führt, weil ich persönlich davon überzeugt bin, dass wir da die paar Prozent mehr rausholen können, um das Outcome für unsere Patienten zu verbessern."

*‘I'm just thinking that this would actually be important and I hope that your project - maybe something will come out of it that will lead to an improvement, because I'm personally convinced that we can get a few per cent more out of it to improve the outcome for our patients.’*

Code: ● Sinnhaftigkeit der Prähabilitation	Weight score: 0

C1 200224, 723 - 726

Created: 01.05.2024 21:43 by jmlut, Modified: 01.05.2024 21:43 by jmlut

Area: 302	0.69%

11.

"Und da haben wir Nachholbedarf."

*‘And we have some catching up to do.’*

Code: ● Sinnhaftigkeit der Prähabilitation	Weight score: 0

C1 200224, 728 - 728

Created: 01.05.2024 21:43 by jmlut, Modified: 01.05.2024 21:43 by jmlut

Area: 31	0.07%

12.

"Je größer der Kreis wird, und bei uns betrifft es insbesondere die Pflege, desto schwieriger ist es dann, die so mitzunehmen und denen zu erklären, warum ein gewisser Mehraufwand notwendig ist um dieses Ziel zu erreichen, dass die Komplikationsrate sinkt, wenn man es jetzt einmal ganz salopp formuliert."

*The larger the circle becomes, and in our case it particularly affects the nursing staff, the more difficult it is to take them along and explain to them why a certain amount of additional work is necessary to achieve this goal of reducing the complication rate, to put it bluntly."*

Code: ● Sinnhaftigkeit der Prähabilitation	Weight score: 0

C2 180324, 266 - 269

Created: 20.05.2024 21:23 by Jan, Modified: 20.05.2024 21:23 by Jan

Area: 307	0.90%

6.2. Motivation

1.

"Also die Bereitschaft ist grundsätzlich natürlich da, aus meiner Sicht, weil das System entwickelt sich täglich weiter. Also ich habe täglich am Tisch irgendwelche Sachen, die wir neu machen und die neu kommen.
Also das ist dem System immanent, dass laufende neue Dinge reinkommen. Also das ist jetzt nicht, es ist kein starrer Block, wo nichts mehr Neues dazukommt,"

*"So the willingness is basically there, of course, from my point of view, because the system continues to develop every day. So I have new things at the table every day that we do and that are new.*

*So it's inherent to the system that new things come in all the time. So it's not, it's not a rigid block where nothing new is added."*

Code: ● Motivation	Weight score: 0

S1 080324, 327 - 332

Created: 20.05.2024 17:19 by Jan, Modified: 20.05.2024 17:19 by Jan

Area: 387	1.49%

2.

"Das verdrängen wir ja global. Also, das ist ja lästig. Patient, der Angst hat und der viel Fragen stellt."

*"We suppress that globally. Well, that's annoying. Patients who are afraid and ask a lot of questions."*

Code: ● Motivation	Weight score: 0

P1 010324, 282 - 283

Created: 20.05.2024 18:50 by Jan, Modified: 20.05.2024 18:50 by Jan

Area: 106	0.28%

3.

"wenn man - weiß ich jetzt nicht, wie einfühlsam Aufklärung stattfindet, aber schnell, schnell wahrscheinlich. Und dann hat man halt für dieses tiefergehende Emotionale halt keinen Raum. Und das kommt dann nicht nachher."

*"If you - I don't know how sensitively education takes place, but quickly, quickly probably. And then there's no room for this deeper emotional aspect. And that doesn't come afterwards."*

Code: ● Motivation	Weight score: 0

P1 010324, 290 - 292

Created: 20.05.2024 18:51 by Jan, Modified: 20.05.2024 18:51 by Jan

Area: 221	0.58%

4.

"Und nachdem in unserem Haus in anderen Bereichen auch sehr viel Forschung läuft, wie das auch sozusagen nachvollziehbar und auch ganz schlüssig, logisch, dass wir auch in dem Bereich in diesem Hotspot -Prähabilitation - natürlich aufgesprungen sind, weil wir als Haus, wie wir halt sozusagen mit unserer, sozusagen Linie von minimalinvasiver Therapie, verbessertem Outcome, auch eben geringere Wunden, schnelleres Erholen, da natürlich die Prähabilitation schon auch als Möglichkeit sehen, die Patienten schneller wieder in den Alltag zurück zu bekommen."

*‘And since a lot of research is also being carried out in other areas at our hospital, it's understandable and logical that we naturally jumped into this hotspot - prehabilitation - because as a hospital, we see prehabilitation as an opportunity to get patients back into everyday life more quickly with our line of minimally invasive therapy, improved outcome, fewer wounds and faster recovery, so to speak.’*

Code: ● Motivation	Weight score: 0

A1 210224, 222 - 229

Created: 09.05.2024 17:22 by jmlut, Modified: 09.05.2024 17:22 by jmlut

Area: 561	1.41%

5.

"Also was ganz generell ein Thema ist, ist, dass alles was Neu ist einem gewissen Widerstand unterliegt. Das ist einfach der Gedanke hinter dem Change Management. Alles Neue hat einmal eine Bremse, die dem entgegen steht.
Die Bremse ist konkret hier einmal, welche Mitarbeiter sollen das zusätzlich machen, dann auch die Mitarbeiter dafür zu motivieren, überhaupt einmal dafür zu gewinnen, was bringt es überhaupt? Also diese Awareness überhaupt zu schaffen, die Daten zu bringen, das die Menschen auch mitarbeiten wollen."

*"So what is generally an issue is that everything that is new is subject to a certain amount of resistance. That's simply the idea behind change management. Everything new has a brake that stands in the way.*

*In concrete terms, the brake here is which employees should also do it, and then motivating employees to do it in the first place, what is the point? In other words, creating this awareness in the first place, providing the data so that people also want to work with it."*

Code: ● Motivation	Weight score: 0

A1 210224, 248 - 255

Created: 09.05.2024 17:23 by jmlut, Modified: 09.05.2024 17:23 by jmlut

Area: 544	1.37%

6.

"Dann gibt es natürlich zahlreiche kommunale Träger nach wie vor und Landeshäuser, Organisationen, Träger, also jedes Bundesland hat einen eigenen Krankenhausträger, also im Landesbesitz. Die haben, die sind irgendwie so eine Mischung zwischen ein bisschen Forschung, ein bisschen Innovation, die tun sich zum Teil ganz schwer und da hängt es dann häufig an einzelnen Köpfen in jeweiligen Kliniken, ob da jetzt noch was speziell gepusht wird oder nicht, aber ich glaube, damit hast du schon ein bisschen Eindruck bekommen, dass das eine sehr zerklüftete Krankenhauslandschaft ist, wo man nicht ein Konzept für alle
ausholen kann."

*"Then, of course, there are still numerous municipal providers and state hospitals, organisations, providers, so each federal state has its own hospital provider, i.e. state-owned. They are somehow a mixture of a bit of research, a bit of innovation, some of them are struggling and it often depends on individual heads in the respective hospitals whether something special is pushed or not, but I think that gives you a bit of an impression that this is a very fragmented hospital landscape, where you can't come up with one concept for all."*

Code: ● Motivation	Weight score: 0

A1 210224, 486 - 494

Created: 09.05.2024 17:59 by jmlut, Modified: 09.05.2024 17:59 by jmlut

Area: 636	1.60%

7.

"Und bei den Chirurgien ist es tatsächlich auch so, das sind wirklich auch nur, aber es sind nicht nur Chirurgen, sondern bei den Klinikern in Österreich sind das halt einzige Macher, die das halt für ihren Bereich als sinnvolle erachten und halt umsetzen möchten.
Aber da ist jetzt, da steht keine, wie ich vorhin gesagt habe, keine Gesellschaft oder Struktur dahinter."

*"And in the case of the surgeons, it really is the same, they are really just surgeons, but they are not just surgeons, but among the clinicians in Austria, they are just the only ones who consider it useful for their field and want to implement it.*

*But, as I said earlier, there is no organisation or structure behind it."*

Code: ● Motivation	Weight score: 0

A1 210224, 533 - 539

Created: 09.05.2024 18:01 by jmlut, Modified: 09.05.2024 18:01 by jmlut

Area: 391	0.98%

8.

"Also bei den Strukturen, in denen ich noch gearbeitet habe, hätte das ein enormer Zeit- und Kraftaufwand bedurft, um da auch die nötige Motivation bei den chirurgischen Partnern hervorzurufen."

*‘With the structures I was still working in, it would have taken an enormous amount of time and effort to generate the necessary motivation among the surgical partners.’*

Code: ● Motivation	Weight score: 0

A2 220422, 138 - 140

Created: 09.05.2024 19:38 by jmlut, Modified: 09.05.2024 19:38 by jmlut

Area: 194	0.54%

9.

"Ja, natürlich geht es ums Geld und es geht tatsächlich ja darum, dass man um neue Strukturen oder neue Projekte zu implementieren, motivierte und engagierte Mitarbeiter braucht. Und es ist sehr einfach, in der derzeitigen Situation ist es einfach so angespannt, dass die Kolleginnen und Kollegen nicht mehr so bereit sind, von ihrer Freizeit sehr viel in die Arbeit zu investieren, sagen wir es einmal ganz vorsichtig.
Nicht weil sie unendlich faul sind, sondern weil da irgendetwas fehlt an Anreiz."

*"Yes, of course it's about money and it's really about the fact that you need motivated and committed employees to implement new structures or new projects. And it's very simple, in the current situation it's just so tense that colleagues are no longer so willing to invest a lot of their free time in work, let's put it mildly.*

*Not because they are infinitely lazy, but because there is a lack of incentive."*

Code: ● Motivation	Weight score: 0

A2 220422, 571 - 578

Created: 09.05.2024 19:52 by jmlut, Modified: 09.05.2024 19:52 by jmlut

Area: 522	1.46%

10.

"Ja, das sehe ich schon so. Dass die dies erwarten, dass man alles, was jetzt nicht in der gewohnten Versorgungsroutine drinnen ist oder in unsere Neue zu projektieren ist, bitte schön nicht in der regulären Arbeitszeit machen. Und da, das ist sicher eine Riesenschere, weil die Kolleginnen und Kollegen und aus dem pflegenden Berufen, das ist alles miteinander, die sind nicht mehr bereit, da 300 Überstunden zu leisten im Monat."

"*Yes, I can see that. They expect that everything that is not part of the usual care routine or that needs to be planned into our new routine should not be done during regular working hours. And that's certainly a huge gap, because colleagues in the caring professions, they're all together, they're no longer prepared to put in 300 hours of overtime a month."*

Code: ● Motivation	Weight score: 0

A2 220422, 587 - 593

Created: 09.05.2024 19:53 by jmlut, Modified: 09.05.2024 19:53 by jmlut

Area: 451	1.26%

11.

"Weil die Menschen irgendwie nicht mehr zufrieden sind, wie sie die Arbeit leisten. Das ist mein Eindruck, aber da kann es ja subjektiv überlagert sein, weil ich nicht mehr zufrieden war, wie von uns erwartet wird, dass man arbeitet und dass da interdisziplinär was ist oder eine Kommunikation erfolgt und zwar altmodische, entweder Face-to-Face oder mittels Telefonhörer und nicht digital über irgendwelche E -Mails."

*"Because people are somehow no longer satisfied with how they do their work. That's my impression, but it may be subjectively superimposed because I was no longer satisfied with how we are expected to work and that there is interdisciplinary work or communication, either face-to-face or by telephone and not digitally via e-mails."*

Code: ● Motivation	Weight score: 0

A2 220422, 626 - 631

Created: 09.05.2024 19:54 by jmlut, Modified: 09.05.2024 19:54 by jmlut

Area: 421	1.18%

12.

"Oder die perioperative Betreuung des Patienten - mit dem befassen wir uns intensiv und es ist sogar so eben, dass wir das eigentlich jetzt auch umsetzen wollen, nur nicht unter der Namen Prähabilitation, sondern unter der Namen perioperative Optimierung, beziehungsweise ERAS. Wobei ERAS der Name ja geschützt ist und das würden wir das wahrscheinlich präoperative Optimierung nennen.
Da bin ich in Kontakt mit unseren Intensivmedizinern, wenn wir das bei großen Operationen, Ösophagus Magen, Leber, Pankreas, da würden wir das gerne machen, weil wir glauben, dass da Verbesserungspotenzial da ist."

*"Or the perioperative care of the patient - we are working intensively on this and it's actually the case that we want to implement this now, but not under the name of prehabilitation, but under the name of perioperative optimisation, or ERAS. Whereby ERAS is a protected name and we would probably call it preoperative optimisation.*

*I'm in contact with our intensive care physicians about doing this for major operations, oesophagus, stomach, liver, pancreas, because we believe that there is potential for improvement."*

Code: ● Motivation	Weight score: 0

C1 200224, 67 - 76

Created: 01.05.2024 16:13 by jmlut, Modified: 01.05.2024 16:13 by jmlut

Area: 623	1.42%

13.

"Also, ich glaube schon, dass es Unterstützung gibt. Zumindest in dem Moment wo ich es - jetzt angenommen, das ist jetzt eh so, dass habe ich jetzt mit dem ERAS, wenn man das jetzt umlegen würde, Prähabilitation, und ich gehe zu meinem Chef und sage, das sollen wir machen, sagt er gut super Idee, machen Sie das. Gut mache ich."

"*Well, I do believe that there is support. At least at the moment when I - assuming that's the case now anyway, that I now have with the ERAS, if you were to transfer it now, prehabilitation, and I go to my boss and say we should do that, he says well great idea, do that. I'll do it."*

Code: ● Motivation	Weight score: 0

C1 200224, 379 - 383

Created: 01.05.2024 16:37 by jmlut, Modified: 01.05.2024 16:37 by jmlut

Area: 331	0.75%

14.

"Ich glaube, das ist... aber grundsätzlich glaube ich schon, dass von der Führung her ein Verständnis da ist, aber halt die Probleme oder Ausreden, wie man es halt nennen möchte, sind immer die gleichen und wir schauen."

*"I think that's... but basically I believe that there is an understanding from the management, but the problems or excuses, as you like to call them, are always the same and we look at them."*

Code: ● Motivation	Weight score: 0

C1 200224, 413 - 415

Created: 01.05.2024 16:40 by jmlut, Modified: 01.05.2024 16:40 by jmlut

Area: 220	0.50%

15.

"Also ich glaube, das Grundproblem bei diesem ganzen Konzept an sich ist die Tatsache, dass es verschiedene Player braucht, die alle miteinander arbeiten müssen. Und je kleiner der Kreis ist, desto leichter wird es, persönliche Beweggründe rüberzubringen und damit die Leute zu motivieren."

*"So I think the basic problem with this whole concept is the fact that it needs different players who all have to work together. And the smaller the circle, the easier it is to convey personal motivations and motivate people."*

Code: ● Motivation	Weight score: 0

C2 180324, 261 - 264

Created: 20.05.2024 21:22 by Jan, Modified: 20.05.2024 21:22 by Jan

Area: 291	0.86%

16.

"Je größer der Kreis wird, und bei uns betrifft es insbesondere die Pflege, desto schwieriger ist es dann, die so mitzunehmen und denen zu erklären, warum ein gewisser Mehraufwand notwendig ist um dieses Ziel zu erreichen, dass die Komplikationsrate sinkt, wenn man es jetzt einmal ganz salopp formuliert."

*‘The larger the circle becomes, and in our case it particularly affects the nursing staff, the more difficult it is to take them along and explain to them why a certain amount of additional work is necessary to achieve this goal of reducing the complication rate, to put it bluntly.’*

Code: ● Motivation	Weight score: 0

C2 180324, 266 - 269

Created: 20.05.2024 21:23 by Jan, Modified: 20.05.2024 21:23 by Jan

Area: 307	0.90%

17.

"Ich glaube, es braucht jemanden, der das koordiniert mit viel Liebe fürs Werk, vielleicht so auf die Art, aber dann auch viele Kollegen, die sich davon anstecken lassen und dann damit machen und das entsprechend auch an den Patienten übertragen können."

*"I think it needs someone who coordinates it with a lot of love for the work, perhaps in this way, but then also many colleagues who are infected by it and can then do it and transfer it to the patient accordingly."*

Code: ● Motivation	Weight score: 0

C2 180324, 370 - 373

Created: 20.05.2024 21:26 by Jan, Modified: 20.05.2024 21:26 by Jan

Area: 255	0.75%

6.3. Kenntnis der Prähabilitation

1.

"Das stimmt, ich habe mit diesem Thema bisher noch nichts zu tun gehabt,"

*“That's right, I haven't had anything to do with this topic yet."*

Code: ● Kenntnis der Prähabilitation	Weight score: 0

S1 080324, 46 - 46

Created: 22.06.2024 14:50 by Jan, Modified: 22.06.2024 14:50 by Jan

Area: 71	0.27%

2.

"das klingt ja total einleuchtend, aber ich habe das jetzt nicht wirklich am Schirm gehabt, ehrlich gesagt, dass das in Europa schon so gehandhabt wird."

*‘That sounds totally plausible, but I didn't really have it on my radar, to be honest, that that's how it's done in Europe.’*

Code: ● Kenntnis der Prähabilitation	Weight score: 0

PT1 140324, 123 - 125

Created: 20.05.2024 20:01 by Jan, Modified: 20.05.2024 20:01 by Jan

Area: 153	0.43%

3.

"Noch nie in meiner beruflichen Karriere bin ich der Prähabilitation begegnet."

*‘Never before in my professional career have I encountered prehabilitation.’*

Code: ● Kenntnis der Prähabilitation	Weight score: 0

P1 010324, 32 - 32

Created: 20.05.2024 18:16 by Jan, Modified: 20.05.2024 18:17 by Jan

Area: 77	0.20%

4.

"Ja, ich finde das eine super Idee. Muss gestehen, ich bin auf die Idee noch nicht gekommen. Es ist traurig. Ja. allenfalls mal im Krankenhaus, dass es eine Zuweisung gibt, dass sich jemand vor einer Operation fürchtet.
Aber das kommt meiner Meinung nach auch zu selten vor."

*"Yes, I think that's a great idea. I have to admit, I hadn't thought of it before. It's sad. Yes, at most sometimes in hospital, there's a referral that someone is afraid of an operation.*

*But in my opinion, that happens too rarely."*

Code: ● Kenntnis der Prähabilitation	Weight score: 0

P1 010324, 47 - 51

Created: 20.05.2024 18:16 by Jan, Modified: 20.05.2024 18:17 by Jan

Area: 293	0.76%

5.

"Bisher natürlich noch wenig, weil es nicht bezahlt wurde, wir haben natürlich im Rahmen der ambulanten Rehabilitation immer wieder auch davon gesprochen, dass das ein guter Ansatz wäre um die Patienten vorzeitig auch schon fit für operative Eingriffe, bei uns vor allem kardiologische Eingriffe, die ja doch immer intensiver, größer oft einmal mit Herzlungenmaschine und Nierenproblematik verbunden sind, Anämieproblematik verbunden sind, oft multimorbide Patienten. Das wurde besprochen, aber meistens noch nicht in der Realität umgesetzt, sondern ich selbst, wir haben keine Präehabilitation gemacht, sondern vor allem Rehabilitation."

*"So far, of course, very little, because it hasn't been paid for. Of course, in the context of outpatient rehabilitation, we have repeatedly talked about the fact that this would be a good approach to getting patients fit for surgical interventions early on, especially cardiological interventions, which are becoming more and more intensive, often involving a heart-lung machine and kidney problems, anaemia problems, often multimorbid patients. This was discussed, but mostly not yet implemented in reality, but I myself, we didn't do prehabilitation, but mainly rehabilitation."*

Code: ● Kenntnis der Prähabilitation	Weight score: 0

K1 280224, 132 - 140

Created: 09.05.2024 20:26 by jmlut, Modified: 09.05.2024 20:26 by jmlut

Area: 644	1.74%

6.

"Also wir haben zum Thema Prähabilitation tatsächlich schon Wochenfortbildungen auch in unserer Abteilung gehalten, wo wir das Team in diese Richtung sensibilisiert haben, dass die Prähabilitation neben zum Beispiel der optimalen Nahrungseinstellung präoperativ auch der Einstellung der internistischen Vorerkrankungen eine Homöostase praktisch herzustellen, sozusagen vorteilhaft ist für das postoperative Outcome.
Natürlich kenne ich es auch von Kongressen und von Artikeln. Da haben wir einiges dazu auch wie gesagt gelesen. Tatsächlich ist es so, dass wir jetzt momentan Prähabilitation bei uns starten als Pilotprojekt.
Ein Kollege wird seinen PhD in diesem Bereich auch durchführen und das heißt ausgewählte Patientengruppen im Bereich kolorektaler Chirurgie werden jetzt in diesen Monaten erstmalig in unserem Haus einer Prähabilitation zugeführt."

*"We have actually already held weekly training sessions on the subject of prehabilitation in our department, where we have sensitised the team to the fact that prehabilitation is beneficial for the post-operative outcome, in addition to, for example, the optimal preoperative nutritional adjustment and the adjustment of pre-existing internal diseases to create a practical homeostasis.*

*Of course, I also know this from congresses and articles. As I said, we have read a lot about this. In fact, we are currently starting prehabilitation as a pilot project. A colleague will also be doing his PhD in this area, which means that selected patient groups in the field of colorectal surgery will be undergoing prehabilitation at our hospital for the first time in the next few months."*

Code: ● Kenntnis der Prähabilitation	Weight score: 0

A1 210224, 34 - 47

Created: 09.05.2024 17:10 by jmlut, Modified: 09.05.2024 17:10 by jmlut

Area: 898	2.26%

7.

"Das ist einmal sicher das große Thema. Die Eindringung von der Prähabilitation generell bei den Mitarbeitern ist unterschiedlich gut meines Erachtens. Also die Aufgeschlossenen haben es natürlich schon wahrgenommen, sowohl in der Physiotherapie als auch in der medizinischen Schiene."

*"That is certainly the big issue. In my opinion, the penetration of prehabilitation in general among employees varies. Of course, those who are open-minded have already noticed it, both in physiotherapy and in the medical track."*

Code: ● Kenntnis der Prähabilitation	Weight score: 0

A1 210224, 151 - 154

Created: 09.05.2024 17:15 by jmlut, Modified: 09.05.2024 17:15 by jmlut

Area: 286	0.72%

8.

"In diesem Ausmaß der drei Säulen betreffend nicht, wo es mal doch etwas angefangen hat, in unserem Schwerpunkthaus war eine Optimierung grad der kardiorespiratorischen Seite wenn notwendig. Ich kenne eine Abteilung, die auch auf den Ernährungsaspekt intensiv eingegangen ist. Ich kenne keine Abteilung, die im präoperativer Setting auf psychologische
Aspekte eingegangen ist."

*“Not to this extent in terms of the three pillars, although it did start a little at one point, in our centre of gravity an optimisation of the cardiorespiratory side was necessary. I know of one department that also focussed intensively on the nutritional aspect. I don't know of any department that dealt with psychological aspects in the pre-operative setting."*

Code: ● Kenntnis der Prähabilitation	Weight score: 0

A2 220422, 39 - 45

Created: 09.05.2024 19:34 by jmlut, Modified: 09.05.2024 19:34 by jmlut

Area: 397	1.11%

9.

"Also ich kenne ja nur im engeren Umfeld Krankenhäuser und da habe ich den Eindruck, dass der Stellenwert noch nicht so ganz durchgedrungen ist, wo er sein sollte und dass es gibt ja inzwischen Daten, dass eine Optimierung präoperativ, sehr wohl das Outcome des Patienten verbessert, dass wir da noch einen enormen Handlungsbedarf haben, um das wirklich präoperativ optimieren zu können und flächendeckend umzusetzen."

*‘Well, I only know hospitals in the immediate vicinity and I have the impression that the importance of this has not yet fully penetrated where it should be and that there is now data that optimisation preoperatively does indeed improve the patient's outcome, that we still have an enormous need for action in order to really be able to optimise this preoperatively and implement it across the board.’*

Code: ● Kenntnis der Prähabilitation	Weight score: 0

A2 220422, 65 - 70

Created: 09.05.2024 19:35 by jmlut, Modified: 09.05.2024 19:35 by jmlut

Area: 421	1.18%

10.

"Also von Eras Plus sind wir weit entfernt, weil wir nicht einmal Eras haben, sagen wir mal so."

*‘So we're a long way from Eras Plus, because we don't even have Eras, let's put it that way.’*

Code: ● Kenntnis der Prähabilitation	Weight score: 0

C1 200224, 100 - 101

Created: 01.05.2024 16:14 by jmlut, Modified: 01.05.2024 16:14 by jmlut

Area: 95	0.22%

11.

"Also wir - über Prähabilitation muss ich gestehen, dezidiert, wenn dann nur so ein bisschen unterbewusst, aber so intensiv, dass jemand mich darauf angesprochen hätte, ob wir das machen wollen und dass das mit Psychologie ist und so weiter, so wirklich, dass ich damit konfrontiert worden bin, eigentlich nicht, nein. Also wenn, dann halt nur mit der perioperativen Optimierung und mit ERAS, aber mit Prähabilitation eigentlich so in dem Sinn, dass mich da jemand angesprochen hätte, ob ich so ein Programm mitmachen möchte oder so, ganz ehrlich nein."

*"So we - about prehabilitation, I have to admit, decidedly, if then only a bit subconsciously, but so intensively that someone would have asked me if we wanted to do it and that it was with psychology and so on, so really that I was confronted with it, not really, no. So if I had, then only with perioperative optimisation and ERAS, but with prehabilitation in the sense that someone would have asked me if I wanted to take part in a programme like that or something, honestly no."*

Code: ● Kenntnis der Prähabilitation	Weight score: 0

C1 200224, 106 - 113

Created: 01.05.2024 16:15 by jmlut, Modified: 01.05.2024 16:15 by jmlut

Area: 558	1.27%

7. Guidelines, Empfehlungen

7.1. Einheitliches Konzept, Evidenz

1.

"Ich glaube, das, was Sie sagen, dass man irgendwo eine vernünftige Evidenz generiert, ja, mit einem guten Studiendesign, das dürfte wahrscheinlich mal das um und auf sein.
Um das überhaupt jemandem schmachhaft zu machen im Gesundheitssystem, und dann bedarf es, das habe ich so aus Ihrer Problembeschreibung, die Sie mir da mitgeschickt hatten, herausgelesen, natürlich aus meiner Sicht schon auch einen einheitlichen Standard für einfach das Vorhaben, also unter anderem, welche Berufsgruppen sind drin, was sollte man überhaupt machen, also dass man dort einen Standard auf die Reihe kriegt und eine vernünftige Leitlinie vielleicht dazu erstellt, wäre ja aus meiner Sicht auch sicher sehr, sehr notwendig."

*"I believe that what you say about generating reasonable evidence somewhere, yes, with a good study design, that should probably be the be-all and end-all.*

*In order to make this at all shameful for someone in the healthcare system, and then, as I read from your description of the problem that you sent me, in my view there is of course also a need for a uniform standard for the project, i.e. among other things, which professional groups are involved, what should be done at all, so that a standard can be established and a sensible guideline perhaps drawn up, would certainly be very, very necessary in my view."*

Code: ● Einheitliches Konzept, Evidenz	Weight score: 0

S1 080324, 120 - 130

Created: 20.05.2024 16:58 by Jan, Modified: 20.05.2024 16:59 by Jan

Area: 734	2.82%

2.

"Evidenz generieren ist wichtig. Auch eine strukturierte Leitlinie oder ein Standard für das Ganze ist aus meiner Sicht wichtig."

*"Generating evidence is important. A structured guideline or a standard for the whole thing is also important in my view."*

Code: ● Einheitliches Konzept, Evidenz	Weight score: 0

S1 080324, 145 - 146

Created: 20.05.2024 17:00 by Jan, Modified: 20.05.2024 17:00 by Jan

Area: 128	0.49%

3.

"Aber wenn man es denn haben möchte, dann wäre das wahrscheinlich irgendwo, wo man ansetzt und mal ein Modellprojekt, also Evidenz generiert und daraus aufstellend, ein Modellprojekt macht und schaut, dass man das mal in zwei, drei Regionen pilotiert."

*‘But if you want to have it, then it would probably be somewhere where you start a model project, i.e. generate evidence and set up a model project and see if you can pilot it in two or three regions.’*

Code: ● Einheitliches Konzept, Evidenz	Weight score: 0

S1 080324, 305 - 308

Created: 20.05.2024 17:15 by Jan, Modified: 20.05.2024 17:15 by Jan

Area: 253	0.97%

4.

"Die müssen ein gewisses Programm haben, sonst kriegen ja gar nicht die Zertifizierung dafür."

*‘They have to have a certain programme, otherwise they won't get the certification for it.’*

Code: ● Einheitliches Konzept, Evidenz	Weight score: 0

PT1 140324, 336 - 337

Created: 20.05.2024 20:08 by Jan, Modified: 20.05.2024 20:08 by Jan

Area: 93	0.26%

5.

"Und wenn man da vorrechnen kann, das bringt so viel, weil du nachher einen weniger langen Aufenthalt in der Intensiv hast, also das müsste man nachher anders aufschlüsseln und so in der Schiene erklären, weil sonst interessiert das keinen Menschen."

*‘And if you can calculate that it's worth so much because you'll have a shorter stay in intensive care afterwards, then you'd have to break it down differently and explain it in this way, because otherwise nobody would be interested.’*

Code: ● Einheitliches Konzept, Evidenz	Weight score: 0

PT1 140324, 392 - 395

Created: 20.05.2024 20:10 by Jan, Modified: 20.05.2024 20:10 by Jan

Area: 251	0.70%

6.

"Aber wenn du eine gescheite Studie machst und andere Studien hernimmst und mit denen an die Sozialversicherungsträger rangehst und denen sagst: Schaut hier, ihr spart euch Geld, wenn ihr das vorher macht mit nachher, dann wäre das doch eine Zukunft."

*‘But if you do a proper study and take other studies and approach the social insurance providers with them and tell them: Look here, you'll save money if you do this before and after, then that would be the future.’*

Code: ● Einheitliches Konzept, Evidenz	Weight score: 0

P1 010324, 578 - 581

Created: 20.05.2024 18:56 by Jan, Modified: 20.05.2024 18:56 by Jan

Area: 252	0.66%

7.

"Für die Prähabilitation hat es immer nur Anzeichen, aber keine guten Daten gegeben, dass davon der Patient so stark profitieren kann."

*‘For prehabilitation, there have only ever been indications, but no good data, that the patient can benefit so much from it.’*

Code: ● Einheitliches Konzept, Evidenz	Weight score: 0

K1 280224, 165 - 167

Created: 09.05.2024 20:27 by jmlut, Modified: 09.05.2024 20:27 by jmlut

Area: 135	0.36%

8.

"Also ich glaube, dass das auch sehr wichtig ist, dass man wirklich auch gute Daten dafür hat, dass die Patienten davon profitieren, dass sie kürzere Liegedauern im Krankenhaus haben, dass sie länger besser ambulant geführt werden können, dass sie einfach gut mitmachen - ist die Frage, ob man das schafft, aber wenigstens eben weniger Hospitalisierung und weniger lange, weniger Rehospitalisierungen und weniger lange Krankenhausaufenthalte und vor allem weniger Intensivtage."

*‘So I believe that it is also very important to have really good data to show that patients benefit from shorter hospital stays, that they can be managed better on an outpatient basis for longer, that they simply participate well - the question is whether you can achieve this, but at least fewer hospitalisations and fewer long stays, fewer rehospitalisations and fewer long stays in hospital and, above all, fewer intensive care days.’*

Code: ● Einheitliches Konzept, Evidenz	Weight score: 0

K1 280224, 345 - 351

Created: 09.05.2024 20:33 by jmlut, Modified: 09.05.2024 20:33 by jmlut

Area: 482	1.30%

9.

"Genau. Und dann ist ja immer die Frage, was heißt auch etabliert und standardisiert, weil es gibt sehr viele unterschiedliche publizierte Konzepte. Grundsätzlich muss es ja ganz ein eher einfaches Konzept sein, damit es die Struktur nicht überfordert, den Patient nicht berfordert, die Compliance aber trotzdem passt.
Dass Patienten, dass er drei, viermal die Woche gewisse Maßnahmen sozusagen durchführt. Und hier gibt es noch nicht wirklich das Verfahren, dass sich jetzt international durchgesetzt hat, dass scheint sich erst herauszukristallisieren."

*"Exactly. And then there's always the question of what established and standardised means, because there are so many different published concepts. Basically, it has to be a rather simple concept so that it doesn't overburden the structure, doesn't overwhelm the patient, but still ensures compliance.*

*That patients carry out certain measures three or four times a week, so to speak. And the procedure that has now become established internationally doesn't really exist here yet, it seems to be just beginning to crystallise."*

Code: ● Einheitliches Konzept, Evidenz	Weight score: 0

A1 210224, 90 - 98

Created: 09.05.2024 17:13 by jmlut, Modified: 09.05.2024 17:13 by jmlut

Area: 579	1.46%

10.

"Es ist ja auch noch die Frage, und wir haben das ja vorher gerade gesagt, unterschiedliche Konzepte für Prähabilitation. Es ist noch gar nicht klar, wie viel Prähabilitation überhaupt bringt und welches, ob es vielleicht ein Konzept gibt für Prähabilitation, das besser ist als wie das andere. Also da weiß man noch nicht so ganz wirklich viel."

*"There is also the question, and we have just said this before, of different concepts for prehabilitation. It's not even clear yet how much prehabilitation is actually useful and which one, whether there is perhaps a concept for prehabilitation that is better than the others. So we don't really know that much yet."*

Code: ● Einheitliches Konzept, Evidenz	Weight score: 0

A1 210224, 193 - 197

Created: 09.05.2024 17:19 by jmlut, Modified: 09.05.2024 17:19 by jmlut

Area: 348	0.88%

11.

"Insofern ist das, was wir jetzt machen, einmal ganz gut und auch als Pilot einmal sicher ein ganz wesentlicher Punkt. Aber mehr kann es derzeit gar nicht, also wird es aus meiner Sicht auch aus unserer Sicht gar nicht wirklich Sinn machen, wenn man sagt, wir unterziehen jetzt alle Patienten sofort der Prähabilitation, weil es noch gar nicht so klar ist, was jetzt das Richtige ist."

*"In this respect, what we are doing now is quite good and, as a pilot, certainly a very important point. But that's all it can do at the moment, so I don't think it really makes sense for us to say that we're going to subject all patients to prehabilitation immediately because it's not yet clear what the right thing to do is."*

Code: ● Einheitliches Konzept, Evidenz	Weight score: 0

A1 210224, 199 - 203

Created: 09.05.2024 17:21 by jmlut, Modified: 09.05.2024 17:21 by jmlut

Area: 387	0.97%

12.

"Das heißt aber auch, man muss einmal zuerst zeigen, dass es tatsächlich einen Benefit bringt mit dem vorgegebenen Konzept."

*‘But that also means that you first have to show that there is actually a benefit with the given concept.’*

Code: ● Einheitliches Konzept, Evidenz	Weight score: 0

A1 210224, 361 - 362

Created: 09.05.2024 17:29 by jmlut, Modified: 09.05.2024 17:29 by jmlut

Area: 123	0.31%

13.

"Das Thema ist grundsätzlich sehr gut. Du hast es schon angesprochen, andere Länder sind da viel weiter. Ich möchte wirklich sehen und die Daten kenne ich noch zu wenig, wie viel es jetzt wirklich outcome-relevant ist, also im Sinne auch von früherer Entlassung, früherer Rückkehr in den Alltag.
Wenn das nämlich wirklich so der große, wenn es wirklich ein relevanter Punkt ist, dann wird man es natürlich mehr implementieren, das ist ganz klar. Ich glaube schon, dass wir in vielen Bereichen unserer perioperativen Medizin noch deutlich besser werden können."

*"The topic is basically very good. You've already mentioned it, other countries are much further ahead. I would really like to see, and I don't know enough about the data yet, how much it is really outcome-relevant, in the sense of earlier discharge, earlier return to everyday life.*

*If that really is the big thing, if it really is a relevant point, then of course it will be implemented more, that's quite clear. I do believe that we can improve significantly in many areas of our perioperative medicine."*

Code: ● Einheitliches Konzept, Evidenz	Weight score: 0

A1 210224, 619 - 627

Created: 09.05.2024 18:04 by jmlut, Modified: 09.05.2024 18:04 by jmlut

Area: 582	1.47%

14.

"Und wenn man so ein Projekt flächendeckend durchziehen will, wird man irgendwo belegen müssen, dass das wirtschaftlich interessant ist."

*‘And if you want to implement a project like this across the board, you will have to prove somewhere that it is economically viable.’*

Code: ● Einheitliches Konzept, Evidenz	Weight score: 0

A2 220422, 224 - 225

Created: 09.05.2024 19:41 by jmlut, Modified: 09.05.2024 19:41 by jmlut

Area: 136	0.38%

15.

"größere randomisiert kontrollierte Studien, insbesondere jetzt für meinen Teilbereich, die Kolonchirurgie, fehlen einfach, die gibt es de facto noch nicht."

*‘Larger randomised controlled studies, especially now for my sub-area, colon surgery, are simply lacking, they don't actually exist yet.’*

Code: ● Einheitliches Konzept, Evidenz	Weight score: 0

C2 180324, 87 - 89

Created: 20.05.2024 21:17 by Jan, Modified: 20.05.2024 21:17 by Jan

Area: 157	0.46%

7.2. Fachgesellschaften, Organisationen

1.

"das kann auch die österreichische Fachgesellschaft aufstellen, wenn sie genug mit Mittel dazu hat, dass sie sich so ein Leitlinienprozess gönnen kann."

*‘The Austrian specialist society can also set this up if it has enough resources to allow itself such a guideline process.’*

Code: ● Fachgesellschaften, Organisationen	Weight score: 0

S1 080324, 183 - 185

Created: 20.05.2024 17:03 by Jan, Modified: 20.05.2024 17:03 by Jan

Area: 152	0.58%

2.

"aber weiß natürlich, dass es mit Kosten verbunden ist, so eine Leitlinienrecherche und das Aufstellen von Leitlinien. Also ganz einfach ist es nicht."

*"But of course I know that there are costs involved in researching guidelines and drawing them up. So it's not that simple."*

Code: ● Fachgesellschaften, Organisationen	Weight score: 0

S1 080324, 187 - 190

Created: 20.05.2024 17:03 by Jan, Modified: 20.05.2024 17:03 by Jan

Area: 167	0.64%

3.

"Und dann bräuchte es aus meiner Sicht auch noch, aber das ist Gegenstand von so einem Standard oder von einer Richtlinie, irgendeinen patientenführenden Arzt auch für das Ganze."

*‘And then, from my point of view, there would also need to be, but this is the subject of a standard or guideline, a patient-leading doctor for the whole thing.’*

Code: ● Fachgesellschaften, Organisationen	Weight score: 0

S1 080324, 204 - 206

Created: 20.05.2024 17:05 by Jan, Modified: 20.05.2024 17:05 by Jan

Area: 179	0.69%

4.

"Möglicherweise, wenn ich mir das so mit anderen Ländern anschaue, gerade jetzt im angloamerikanischen Sprachraum, vielleicht auch im skandinavischen, in der skandinavischen Gegend, dann kann es sein, dass uns so übergeordnete, weiß es nicht, Gremien, Organisationen fehlen, die erstens einmal was uns sicher fehlt, große Studien machen, die dann sozusagen national sowie zum Beispiel in Großbritannien große Studien lancieren, die oder auch große Bewegungen lancieren, die dann sozusagen über das ganze Land schwappen."

‘*Possibly, if I look at other countries, especially in the Anglo-American language area, perhaps also in Scandinavia, in the Scandinavian region, then it could be that we lack superordinate, I don't know, committees, organisations, which first of all, what we certainly lack, are large studies, which then launch large studies nationally, as well as in Great Britain, for example, which or also launch large movements, which then spill over the whole country, so to speak.’*

Code: ● Fachgesellschaften, Organisationen	Weight score: 0

A1 210224, 405 - 411

Created: 09.05.2024 17:32 by jmlut, Modified: 09.05.2024 17:32 by jmlut

Area: 524	1.32%

5.

"Das machen Italiener auch schon deutlich besser. So was fehlt, wir haben zwar Fachgesellschaften, für unsere Bereiche, aber nicht so nationale Gremien, die nationale Studien pushen oder so nationale Bewegungen, wie das die Prähabilitation pushen.
Das war ja bei Covid im Prinzip das gleiche, auch da gibt es jetzt kein wirklich gutes nationales Medizingremium, das da nochmal vielleicht Pläne besser ausgearbeitet hat, als wie in anderen Ländern zum Beispiel."

*"The Italians are already doing that much better. We don't have anything like that, we do have specialist societies for our areas, but we don't have national committees that push national studies or national movements that push prehabilitation.*

*It was basically the same with Covid, there's no really good national medical committee that has perhaps worked out plans better than in other countries, for example."*

Code: ● Fachgesellschaften, Organisationen	Weight score: 0

A1 210224, 419 - 426

Created: 09.05.2024 17:33 by jmlut, Modified: 09.05.2024 17:33 by jmlut

Area: 482	1.21%

6.

"Also Österreich ist sicher grundsätzlich einmal, also grundsätzlich muss man sagen, es gibt ja viele Guidelines in anderen Ländern, also vieles, was ich so aus meiner Perspektive sehe, ist ja dann, dass man dann das kopieren könnte, was Größere schon gemacht haben und andere, die schneller sind.
Häufig werden in Österreich dann Richtlinien angewendet, die in Deutschland sozusagen gemacht wurden, oder auf europäischer Ebene oder sonst so gemacht wurden, die kommen dann halt hier auch zur Anwendung."

*"Well, Austria is certainly fundamentally, so basically you have to say, there are many guidelines in other countries, so a lot of what I see from my perspective is that you could then copy what larger countries have already done and others that are faster.*

*In Austria, guidelines are often applied that have been created in Germany, so to speak, or at European level or elsewhere, which are then also applied here."*

Code: ● Fachgesellschaften, Organisationen	Weight score: 0

A1 210224, 431 - 438

Created: 09.05.2024 17:34 by jmlut, Modified: 09.05.2024 17:34 by jmlut

Area: 525	1.32%

7.

"Wenn dann wie zum Beispiel bei der Fachgesellschaft ÖGARI dann jetzt ein neuer Punkt auftaucht, also neues Thema auftaucht, wie zum Beispiel digitale Medizin, Big Data, KI, dann wird es jetzt von, zum Beispiel wird es jetzt im Rahmen der Fachgesellschaft aufgenommen und dort neue Projekte lanciert.
Das sind aber häufig, weil es eine Fachgesellschaft ist, eine wissenschaftliche, sind es halt häufig auch Projekte, die halt dann einen wissenschaftlichen Charakter haben und weniger einen klinisch orientierten sozusagen Ansatz haben."

*"If, for example, a new issue arises at the ÖGARI professional association, a new topic such as digital medicine, big data, AI, then it is now taken up by, for example, the professional association and new projects are launched there.*

*However, because it is a professional society, a scientific one, these are often projects that have a scientific character and less of a clinically orientated approach, so to speak."*

Code: ● Fachgesellschaften, Organisationen	Weight score: 0

A1 210224, 496 - 503

Created: 09.05.2024 18:00 by jmlut, Modified: 09.05.2024 18:00 by jmlut

Area: 557	1.40%

8.

"Wir sehen dann von den einzelnen Menschen ab, wer pusht es. Wenn du jetzt sagst, in UK geht das viel von den Anästhesisten aus, vermutlich wird da auch das Royal College oder sonst ein Anästhesistengesellschaft das grundsätzlich auch gepusht haben und dann über das ganze Land wurde das dann von den Anästhesisten aufgenommen. Bei uns ist etwas derartiges, jetzt im Bereich der Anästhesie- und Intensivmedizingesellschaft der Nationalen nicht passiert, sonst wäre wahrscheinlich auch der Call von sozusagen von der Anästhesieseite viel größer gewesen, könnte ich mir vorstellen."

*"We then look at the individual people, who is pushing it. If you now say that in the UK a lot of this comes from the anaesthetists, presumably the Royal College or another anaesthetic society will also have pushed this in principle and then it was taken up by the anaesthetists across the whole country. Something like this hasn't happened here, now in the area of the National Anaesthesia and Intensive Care Society, otherwise the call from the anaesthesia side, so to speak, would probably have been much greater, I would imagine."*

Code: ● Fachgesellschaften, Organisationen	Weight score: 0

A1 210224, 522 - 529

Created: 09.05.2024 18:00 by jmlut, Modified: 09.05.2024 18:00 by jmlut

Area: 585	1.47%

9.

"Und bei den Chirurgien ist es tatsächlich auch so, das sind wirklich auch nur, aber es sind nicht nur Chirurgen, sondern bei den Klinikern in Österreich sind das halt einzige Macher, die das halt für ihren Bereich als sinnvolle erachten und halt umsetzen möchten.
Aber da ist jetzt, da steht keine, wie ich vorhin gesagt habe, keine Gesellschaft oder Struktur dahinter."

*“And in the case of the surgeons, it's really only surgeons, but it's not just surgeons, it's just that the clinicians in Austria are the only ones who think it makes sense for their field and want to implement it. But, as I said earlier, there is no organisation or structure behind it."*

Code: ● Fachgesellschaften, Organisationen	Weight score: 0

A1 210224, 533 - 539

Created: 09.05.2024 18:01 by jmlut, Modified: 09.05.2024 18:01 by jmlut

Area: 391	0.98%

10.

"Und wie gesagt, also für Österreich grundsätzlich sehe ich die Möglichkeiten, dass man über Fachgesellschaften geht, weil dann kommt der Druck von der Fachgesellschaftsseite oder eben von der Regierungsseite, wobei wenn es ein politischer Prozess sein muss, also der, wo dann die Beamten irgendwas aussprechen muss, die Politik entschieden haben, dann ist es meistens erst spät.
Und in der Regel folgt ja die Politik häufig auch den Meinungen der Bevölkerung oder Strömungen, die sich in der Bevölkerung eh schon sozusagen am durchsetzen sind. Das heißt, das Beste wäre wirklich, wenn hier von der Fachgesellschaft aus Empfehlungen entstehen."

*"And as I said, for Austria I basically see the possibility of going through professional associations, because then the pressure comes from the professional society side or from the government side, whereby if it has to be a political process, i.e. the one where the officials have to pronounce something, the politicians have decided, then it is usually only late.*

*And as a rule, politicians often follow the opinions of the population or currents that are already gaining ground in the population anyway, so to speak. In other words, the best thing would really be if recommendations were made by the specialist society."*

Code: ● Fachgesellschaften, Organisationen	Weight score: 0

A1 210224, 588 - 597

Created: 09.05.2024 18:03 by jmlut, Modified: 09.05.2024 18:03 by jmlut

Area: 668	1.68%

11.

"Ich könnte mir vorstellen, weil du vorhin angesprochen hast, das kommt sonst von den Anästhesisten. Wenn es hier eine Gruppe gäbe zu präoperativer, nicht nur Evaluierung, sondern auch, die gibt das nämlich schon, sondern Optimierung.
Vielleicht gibt es die auch schon. Also vielleicht heißt die auch schon so bei unserer Fachgesellschaft, der Nationalen. Dann könnte die dann eben Empfehlungen abgeben zu Prähabilitation und vielleicht auch zu optimaler Postrehabilitation."

*"I could imagine, because you mentioned earlier, that this would otherwise come from the anaesthetists. If there was a group here for preoperative, not just evaluation, but also, because that already exists, but optimisation.*

*Perhaps it already exists. Perhaps it's already called that by our professional association, the National Association. It could then make recommendations on prehabilitation and perhaps also on optimised post-rehabilitation."*

Code: ● Fachgesellschaften, Organisationen	Weight score: 0

A1 210224, 599 - 605

Created: 09.05.2024 18:04 by jmlut, Modified: 09.05.2024 18:04 by jmlut

Area: 495	1.25%

12.

"Und ich glaube, das ist, glaube ich, das Wichtige. Und da würde ich mir schon wünschen, wenn man sozusagen eine Arge hätte, zum Beispiel die Prähabilitation als Teil von ERAS österreichweit einfach pusht."

*"And I think that's the important thing. And I would like to see a consortium, so to speak, for example, to simply push prehabilitation as part of ERAS throughout Austria."*

Code: ● Fachgesellschaften, Organisationen	Weight score: 0

A1 210224, 650 - 652

Created: 09.05.2024 18:05 by jmlut, Modified: 09.05.2024 18:05 by jmlut

Area: 206	0.52%

13.

"achgesellschaften auf jeden Fall, weil man ja für alles Personal braucht, Politik sehe ich unbedingt gefordert, weil da geht es um das Gemeinwohl, da geht es um Strukturen, die wir in der Zukunft schaffen müssen, um das alles zu bewältigen."

*‘In any case, I see politics as an absolute must, because it's about the common good, it's about structures that we need to create in the future in order to manage all of this.’*

Code: ● Fachgesellschaften, Organisationen	Weight score: 0

A2 220422, 234 - 237

Created: 09.05.2024 19:42 by jmlut, Modified: 09.05.2024 19:42 by jmlut

Area: 243	0.68%

14.

"Ja, und Psychologie eben, weil du das angesprochen hast, habe ich eigentlich so in diesem Haus nicht so gesehen. In einem Haus, wo ich vorher gearbeitet habe, war das bei allen onkologischen Operationen eine Standardzuweisung, weil die zertifiziert wurden."

*"Yes, and psychology, because you mentioned that, I didn't actually see it that way in this hospital. In a hospital where I previously worked, this was a standard assignment for all oncological operations because they were certified."*

Code: ● Fachgesellschaften, Organisationen	Weight score: 0

C1 200224, 351 - 354

Created: 01.05.2024 16:36 by jmlut, Modified: 01.05.2024 16:36 by jmlut

Area: 259	0.59%

15.

"Onkozentrum, also Onkocert, also das ist so ein deutsches Krebszentrum. DKG, glaube ich, also deutsche Krebs Gesellschaft wahrscheinlich ..."

*"Onkozentrum, so Onkocert, that's a German cancer centre. DKG, I think, probably the German Cancer Society ..."*

Code: ● Fachgesellschaften, Organisationen	Weight score: 0

C1 200224, 360 - 361

Created: 01.05.2024 16:36 by jmlut, Modified: 01.05.2024 16:36 by jmlut

Area: 141	0.32%

16.

"Auf alle Fälle, wenn du da dein Zertifikat nicht verlieren willst im Rahmen des Audits, musst du nachweisen, dass du alle onkologischen Patienten auch einem Psychologen zugewiesen hast. Und dadurch ist es halt mitgelaufen.
Also da war das mehr oder weniger Standard, sozusagen, weil das Teil des Zentrum-Zertifikats war, das du das nicht verlierst."

*"In any case, if you don't want to lose your certificate as part of the audit, you have to prove that you have also assigned all oncological patients to a psychologist. And that's how it came about. So that was more or less standard, so to speak, because it was part of the centre certificate that you wouldn't lose."*

Code: ● Fachgesellschaften, Organisationen	Weight score: 0

C1 200224, 363 - 368

Created: 01.05.2024 16:36 by jmlut, Modified: 01.05.2024 16:36 by jmlut

Area: 369	0.84%

17.

"aber ich weiß ehrlich gesagt gar nicht, ob es so Zertifizierungen für Prähabilitation... gibt es sowas?"

*"but I honestly don't know if there are any certifications for prehabilitation... is there such a thing?"*

Code: ● Fachgesellschaften, Organisationen	Weight score: 0

C1 200224, 437 - 438

Created: 01.05.2024 16:48 by jmlut, Modified: 01.05.2024 16:48 by jmlut

Area: 104	0.24%

18.

"Aber das, glaube ich, ist wahrscheinlich ein Schlüssel, dass man schaut, dass man die
Fachgesellschaften zusammenbringt.
Das betrifft einfach mehrere Gesellschaften. Rein am Chirurgiekongress ist es ein, würde ich mal sagen, mittelmäßig unterrepräsentiertes Thema in den letzten Jahren. Es kommt zwar immer - zumindest was Österreich anbelangt - aber gibt es einzelne Beiträge dazu, aber in meinen Augen sehr wenige, muss man sagen, letztlich. Kann aber sein, dass sich das in den nächsten Jahren ändert."

*"But I think that's probably a key, that you have to make sure that you bring the specialist societies together. This simply concerns several societies. I would say that it has been a moderately underrepresented topic at the Surgical Congress in recent years. There are always - at least as far as Austria is concerned - but there are individual contributions, but in my opinion very few, it has to be said. But that may change in the next few years."*

Code: ● Fachgesellschaften, Organisationen	Weight score: 0

C2 180324, 591 - 599

Created: 20.05.2024 21:59 by Jan, Modified: 20.05.2024 21:59 by Jan

Area: 528	1.55%
