# Supplementary material for: Exploring the barriers to prehabilitation integration in Austrian healthcare: a qualitative analysis
Source: BJA Open. 2026 Oct 6;20:100868. doi: 10.1016/j.bjao.2026.100868 (PMC13675892; doi:10.1016/j.bjao.2026.100868)
Supplement: Supplementary file 4 [file mmc4.docx]

**Supplemental Digital Content 4**

*Lutz et al. — Exploring the Barriers to Prehabilitation Integration in Austrian Healthcare: A Qualitative Analysis*

**Figure 1.** Coverage (%) of prehabilitation-related thematic codes across interviews/cases. Each line represents one thematic category; the x-axis shows individual interview codes (S1 = health insurance representative; PT1 = physiotherapist; P1 = psychologist; K1 = cardiologist; A1, A2 = anaesthesiologists; C1, C2 = surgeons); the y-axis shows code coverage as a percentage of total coded text per case. The figure illustrates profession-specific emphases: S1 predominantly discussed economic factors; PT1 and P1 emphasised cultural and patient-related factors; hospital-based physicians (A1, A2, C1, C2) focused on organisational and structural barriers. Despite these professional differences, economic factors and healthcare system structure remained substantially represented across most informants, indicating shared systemic concerns.
